# Supplementary material for: An empirical analysis of the impact of Chinese government investment on high-quality economic development——A study based on spatial Dubin model
Source: PLoS One. 2023 Mar 15;18(3):e0283073. doi: 10.1371/journal.pone.0283073 (PMC10016692; doi:10.1371/journal.pone.0283073)
Supplement: S1 File — This file is mainly the base data used in this paper. (PDF) [file pone.0283073.s001.pdf]

Regional GDP(Unit: 100 million yuan) (Data from China Statistical Yearbook)												
Region	2005	2006	2007	2008	2009	2010	2011	2012	2013	2014	2015	2016
Beijing	6969.52	8117.78	9846.81	11115.00	12153.03	14113.58	16251.93	17879.4	19800.81	21330.83	23014.59	25669.13
Tianjin	3905.64	4462.74	5252.76	6719.01	7521.85	9224.46	11307.28	12893.88	14442.01	15726.93	16538.19	17885.39
Hebei	10012.11	11467.60	13607.32	16011.97	17235.48	20394.26	24515.76	26575.01	28442.95	29421.15	29806.11	32070.45
Shanxi	4230.53	4878.61	6024.45	7315.40	7358.31	9200.86	11237.55	12112.83	12665.25	12761.49	12766.49	13050.41
nner Mongoli	3905.03	4944.25	6423.18	8496.20	9740.25	11672.00	14359.88	15880.58	16916.5	17770.19	17831.51	18128.1
Liaoning	8047.26	9304.52	11164.30	13668.58	15212.49	18457.27	22226.7	24846.43	27213.22	28626.58	28669.02	22246.9
Jilin	3620.27	4275.12	5284.69	6426.10	7278.75	8667.58	10568.83	11939.24	13046.4	13803.14	14063.13	14776.8
Heilongjiang	5513.7	6211.80	7104.00	8314.37	8587.00	10368.60	12582	13691.58	14454.91	15039.38	15083.67	15386.09
Shanghai	9247.66	10572.24	12494.01	14069.86	15046.45	17165.98	19195.69	20181.72	21818.15	23567.7	25123.45	28178.65
Jiangsu	18598.69	21742.05	26018.48	30981.98	34457.30	41425.48	49110.27	54058.22	59753.37	65088.32	70116.38	77388.28
Zhejiang	13417.68	15718.47	18753.73	21462.69	22990.35	27722.31	32318.85	34665.33	37756.58	40173.03	42886.49	47251.36
Anhui	5350.17	6112.50	7360.92	8851.66	10062.82	12359.33	15300.65	17212.05	19229.34	20848.75	22005.63	24407.62
Fujian	6554.69	7583.85	9248.53	10823.01	12236.53	14737.12	17560.18	19701.78	21868.49	24055.76	25979.82	28810.58
Jiangxi	4056.76	4820.53	5800.25	6971.05	7655.18	9451.26	11702.82	12948.88	14410.19	15714.63	16723.78	18499
Shandong	18366.87	21900.19	25776.91	30933.28	33896.65	39169.92	45361.85	50013.24	55230.32	59426.59	63002.33	68024.49
Henan	10587.42	12362.79	15012.46	18018.53	19480.46	23092.36	26931.03	29599.31	32191.3	34938.24	37002.16	40471.79
Hubei	6590.19	7617.47	9333.40	11328.89	12961.10	15967.61	19632.26	22250.45	24791.83	27379.22	29550.19	32665.38
Hunan	6596.1	7688.67	9439.60	11555.00	13059.69	16037.96	19669.56	22154.23	24621.67	27037.32	28902.21	31551.37
Guangdong	22557.37	26587.76	31777.01	36796.71	39482.56	46013.06	53210.28	57067.92	62474.79	67809.85	72812.55	80854.91
Guangxi	3984.1	4746.16	5823.41	7021.00	7759.16	9569.85	11720.87	13035.1	14449.9	15672.89	16803.12	18317.64
Hainan	897.99	1044.91	1254.17	1503.06	1654.21	2064.50	2522.66	2855.54	3177.56	3500.72	3702.76	4053.2
Chongqing	3467.72	3907.23	4676.13	5793.66	6530.01	7925.58	10011.37	11409.6	12783.26	14262.6	15717.27	17740.59
Sichuan	7385.1	8690.24	10562.39	12601.23	14151.28	17185.48	21026.68	23872.8	26392.07	28536.66	30053.1	32934.54
Guizhou	2005.42	2338.98	2884.11	3561.56	3912.68	4602.16	5701.84	6852.2	8086.86	9266.39	10502.56	11776.73
Yunnan	3461.73	3988.14	4772.52	5692.12	6169.75	7224.18	8893.12	10309.47	11832.31	12814.59	13619.17	14788.42
Shaanxi	3933.72	4743.61	5757.29	7314.58	8169.80	10123.48	12512.3	14453.68	16205.45	17689.94	18021.86	19399.59
Gansu	1933.98	2276.70	2702.40	3166.82	3387.56	4120.75	5020.37	5650.2	6330.69	6836.82	6790.32	7200.37
Qinghai	543.32	648.50	797.35	1018.62	1081.27	1350.43	1670.44	1893.54	2122.06	2303.32	2417.05	2572.49
Ningxia	612.61	725.90	919.11	1203.92	1353.31	1689.65	2102.21	2341.29	2577.57	2752.1	2911.77	3168.59
Xinjiang	2604.19	3045.26	3523.16	4183.21	4277.05	5437.47	6610.05	7505.31	8443.84	9273.46	9324.8	9649.7

Number of patent applications(Unit: piece) (Data from China Statistical Yearbook)												
Region	2005	2006	2007	2008	2009	2010	2011	2012	2013	2014	2015	2016
Beijing	22572	26555	31680	43508	50236	57296	77955	92305	123336	138111	156312	189129
Tianjin	11657	13299	15744	18230	19624	25973	38489	41009	60915	63422	79963	106514
Hebei	6401	7220	7853	9128	11361	12295	17595	23241	27619	30000	44060	54838
Shanxi	1985	2824	3333	5386	6822	7927	12769	16786	18859	15687	14948	20031
nner Mongoli	1455	1946	2015	2221	2484	2912	3841	4732	6388	6359	8876	10672
Liaoning	15672	17052	19518	20893	25803	34216	37102	41152	45996	37860	42153	52603
Jilin	4101	4578	5251	5536	5934	6445	8196	9171	10751	11933	14800	18922
Heilongjiang	6050	6535	7242	7974	9014	10269	23432	30610	32264	31856	34611	35293
Shanghai	32741	36042	47205	52835	62241	71196	80215	82682	86450	81664	100006	119937
Jiangsu	34811	53267	88950	128002	174329	235873	348381	472656	504500	421907	428337	512429
Zhejiang	43221	52980	68933	89931	108482	120742	177066	249373	294014	261435	307264	393147
Anhui	3516	4679	6070	10409	16386	47128	48556	74888	93353	99160	127709	172552
Fujian	9460	10351	11341	13181	17559	21994	32325	42773	53701	58075	83146	130376
Jiangxi	2815	3171	3548	3746	5224	6307	9673	12458	16938	25594	36936	60494
Shandong	28835	38284	46849	60247	66857	80856	109599	128614	155170	158619	193220	212911
Henan	8981	11538	14916	19090	19589	25149	34076	43442	55920	62434	74373	94669
Hubei	11534	14576	17376	21147	27206	31311	42510	51316	50816	59050	74240	95157
Hunan	8763	10249	11233	14016	15948	22381	29516	35709	41336	44194	54501	67779
Guangdong	72220	90886	102449	103883	125673	152907	196272	229514	264265	278358	355939	505667
Guangxi	2379	2784	3480	3884	4277	5117	8106	13610	23251	32298	43696	59239
Hainan	498	538	632	873	1040	1019	1489	1824	2359	2416	3127	3658
Chongqing	6260	6471	6715	8324	13482	22825	32039	38924	49036	55298	82791	59518
Sichuan	10567	13109	19165	24335	33047	40230	49734	66312	82453	91167	110746	142522
Guizhou	2226	2674	2759	2943	3709	4414	8351	11296	17405	22467	18295	25315
Yunnan	2556	3085	3108	4089	4633	5645	7150	9260	11512	13343	17603	23709
Shaanxi	4166	5717	8499	11898	15570	22949	32227	43608	57287	56235	74904	69611
Gansu	1759	1460	1608	2178	2676	3558	5287	8261	10976	12020	14584	20276
Qinghai	216	325	387	431	499	602	732	844	1099	1534	2590	3284
Ningxia	516	671	838	1087	1277	739	1079	1985	3230	3532	4394	6149
Xinjiang	1851	2256	2270	2412	2872	3560	4736	7044	8224	10210	12250	14105

Permanent population(Unit: 10,000 people) (Data from China Statistical Yearbook)												
Region	2005	2006	2007	2008	2009	2010	2011	2012	2013	2014	2015	2016
Beijing	1538	1601	1676	1771	1860	1962	2019	2069	2115	2152	2171	2173
Tianjin	1043	1075	1115	1176	1228	1299	1355	1413	1472	1517	1547	1562
Hebei	6851	6898	6943	6989	7034	7194	7241	7288	7333	7384	7425	7470
Shanxi	3355	3375	3393	3411	3427	3574	3593	3611	3630	3648	3664	3682
nner Mongoli	2403	2415	2429	2444	2458	2472	2482	2490	2498	2505	2511	2520
Liaoning	4221	4271	4298	4315	4341	4375	4383	4389	4390	4391	4382	4378
Jilin	2716	2723	2730	2734	2740	2747	2749	2750	2751	2752	2753	2733
Heilongjiang	3820	3823	3824	3825	3826	3833	3834	3834	3835	3833	3812	3799
Shanghai	1890	1964	2064	2141	2210	2303	2347	2380	2415	2426	2415	2420
Jiangsu	7588	7656	7723	7762	7810	7869	7899	7920	7939	7960	7976	7999
Zhejiang	4991	5072	5155	5212	5276	5447	5463	5477	5498	5508	5539	5590
Anhui	6120	6110	6118	6135	6131	5957	5968	5988	6030	6083	6144	6196
Fujian	3557	3585	3612	3639	3666	3693	3720	3748	3774	3806	3839	3874

Jiangxi	4311	4339	4368	4400	4432	4462	4488	4504	4522	4542	4566	4592
Shandong	9248	9309	9367	9417	9470	9588	9637	9685	9733	9789	9847	9947
Henan	9380	9392	9360	9429	9487	9405	9388	9406	9413	9436	9480	9532
Hubei	5710	5693	5699	5711	5720	5728	5758	5779	5799	5816	5852	5885
Hunan	6326	6342	6355	6380	6406	6570	6596	6639	6691	6737	6783	6822
Guangdong	9194	9442	9660	9893	10130	10441	10505	10594	10644	10724	10849	10999
Guangxi	4660	4719	4768	4816	4856	4610	4645	4682	4719	4754	4796	4838
Hainan	828	836	845	854	864	869	877	887	895	903	911	917
Chongqing	2798	2808	2816	2839	2859	2885	2919	2945	2970	2991	3017	3048
Sichuan	8212	8169	8127	8138	8185	8045	8050	8076	8107	8140	8204	8262
Guizhou	3730	3690	3632	3596	3537	3479	3469	3484	3502	3508	3530	3555
Yunnan	4450	4483	4514	4543	4571	4602	4631	4659	4687	4714	4742	4771
Shaanxi	3690	3699	3708	3718	3727	3735	3743	3753	3764	3775	3793	3813
Gansu	2545	2547	2548	2551	2555	2560	2564	2578	2582	2591	2600	2610
Qinghai	543	548	552	554	557	563	568	573	578	583	588	593
Ningxia	596	604	610	618	625	633	639	647	654	662	668	675
Xinjiang	2010	2050	2095	2131	2159	2185	2209	2233	2264	2298	2360	2398

Internal expenditure on R&D expenses(Unit: 10000 yuan) (Data from China Science and Technology Statistical Yearbook)												
Region	2005	2006	2007	2008	2009	2010	2011	2012	2013	2014	2015	2016
Beijing	3820683	4329877	5053870	5503499	6686351	8218234.2	9366439	10633640	11850469	12687953	13840231	14845762
Tianjin	725659	952370	1146921	1557166	1784661	2295643.8	2977580	3604866	4280921	4646868	5101839	5373223
Hebei	589320	766640	900165	1091113	1348446	1554491.9	2013377	2457670	2818551	3130881	3508708	3834274
Shanxi	262814	363388	492506	625574	808563	898835	1133926	1323458	1549799	1521871	1325268	1326237
nner Mongol	116956	164860	241982	338950	520726	637205	851685	1014468	1171877	1221346	1360617	1475124
Liaoning	1247086	1357857	1653989	1900662	2323687	2874702.6	3638348	3908680	4459322	4351851	3633971	3727165
Jilin	393039	409212	508658	528364	813602	758004.8	891337	1098010	1196882	1307243	1414089	1396668
Heilongjiang	489073	570294	660437	866999	1091704	1230433.8	1287788	1459588	1647838	1613469	1576677	1525048
Shanghai	2083538	2588386	3074569	3553868	4233774	4817031.4	5977131	6794636	7767847	8619549	9361439	10493187
Jiangsu	2698292	3460695	4301988	5809124	7019529	8579490.7	10655109	12878616	14874466	16528208	18012271	20268734
Zhejiang	1632921	2240315	2816032	3445714	3988367	4942348.5	5980824	7225867	8172675	9078500	10111792	11306297
Anhui	458994	593365	717914	983208	1359535	1637219.2	2146439	2817953	3520833	3936070	4317511	4751329
Fujian	536186	674333	821721	1019288	1353819	1708982.3	2215151	2709891	3140589	3550325	3929298	4542920
Jiangxi	285314	377619	487867	631468	758936	871527.1	967529	1136552	1354972	1531114	1731820	2073091
Shandong	1951449	2341299	3123081	4337171	5195920	6720045	8443667	10203266	11758027	13040695	14271890	15660904
Henan	555824	798419	1011299	1222763	1747599	2111675.1	2644923	3107802	3553246	4000099	4350430	4941880
Hubei	749531	944297	1113179	1489859	2134490	2641180.3	3230129	3845239	4462043	5108973	5617415	6000423
Hunan	445235	536174	735536	1127040	1534995	1865583.7	2332181	2876780	3270253	3679345	4126692	4688418
Guangdong	2437605	3130433	4042910	5025577	6529820	8087477.6	10454872	12361501	14434527	16054458	17981679	20351440
Guangxi	145947	182403	220030	328306	472028	628696.2	810205	971539	1076790	1119033	1059124	1177487
Hainan	15950	21044	26020	33479	57806	70203.5	103717	137244	148357	169151	169685	217095
Chongqing	319586	369140	469876	601525	794599	1002663.3	1283560	1597973	1764911	2018528	2470012	3021830
Sichuan	965760	1078405	1391401	1602595	2144590	2642695.3	2941009	3508589	3999702	4493285	5028761	5614193
Guizhou	110349	145113	137434	189298	264134	299664.6	363089	417261	471850	554795	623196	734006
Yunnan	213233	209187	258776	309909	372304	441671.8	560797	687548	798371	859297	1093570	1327616
Shaanxi	924462	1013558	1217106	1432726	1895063	2175042.2	2493548	2872035	3427454	3667730	3931727	4195554
Gansu	196136	239530	257220	318014	372612	419384.6	485261	604762	669194	768739	827203	869850
Qinghai	29554	33412	38093	39092	75938	99437.9	125756	131228	137541	143235	115843	139977
Ningxia	31681	49749	74724	75490	104422	115101.3	153183	182304	209042	238580	254842	299269
Xinjiang	64087	84760	100169	160113	218043	266545.4	330031	397289	454598	491587	520010	566301

Total population over 6 years old(Unit: 10000 yuan) (Data from China Statistical Yearbook, China Population and Employment Statistical Yearbook)												
Region	2005	2006	2007	2008	2009	2010	2011	2012	2013	2014	2015	2016
Beijing	196019	13769	13984	14174	14406	18813279	16491	16447	16645	16828	316773	17001
Tianjin	132069	9296	9517	9710	10068	12388491	11019	11175	11582	11935	228196	12401
Hebei	846198	59514	59018	58608	57559	66150575	56753	55844	55688	55991	1066111	57884
Shanxi	416531	29315	29277	29058	28680	33521349	28941	28388	28116	28424	536025	29052
Inner Mongolia	298416	21009	20920	20742	20356	23362679	20015	19598	19503	19516	368717	19913
Liaoning	532275	37610	37666	37511	36994	41873047	35906	35238	34925	34931	652806	35135
Jilin	341975	24073	23898	23694	23274	26136514	22363	21799	21702	21697	409572	21860
Heilongjiang	481167	33944	33610	33148	32556	36619463	31272	30608	30284	30313	572233	30868
Shanghai	227240	16014	16199	16358	16296	22085668	19183	19034	19046	19008	355996	19274
Jiangsu	939782	66239	65988	65610	64329	74119475	63465	62230	61632	61601	1163203	62953
Zhejiang	610761	43128	43378	43524	43157	51484414	44170	43285	43066	43110	810517	44145
Anhui	754241	52930	52212	51447	50649	55103738	47067	46039	45731	46366	884086	48183
Fujian	439048	30740	30699	30264	29789	34366573	29539	28931	28714	28801	546001	29688
Jiangxi	517669	36259	36534	36120	35686	40413800	34969	34348	34378	34372	651438	35394
Shandong	1146600	80837	80466	79888	78686	89358154	76910	75422	75252	75500	1423779	77354
Henan	1148451	80530	80045	78498	77706	85563558	73413	72042	71151	71223	1353044	73743
Hubei	715022	50373	49735	49099	47978	53724341	46101	45109	44496	44681	846998	46004
Hunan	777739	54780	54867	54063	53010	60715957	51951	51099	51107	51363	976881	53099
Guangdong	1144620	80468	80650	80550	80247	97649498	83713	82228	81324	82029	1559777	85168
Guangxi	564039	39831	40026	39706	39345	41837842	36046	35185	35486	35659	675146	36994
Hainan	101346	7113	7139	7049	7008	7949791	6845	6773	6755	6826	129047	7037
Chongqing	348166	24519	24352	24010	23672	26962605	23431	23049	23095	23263	441611	24087
Sichuan	1013364	71822	70787	69797	68373	75277913	64328	63113	62617	62975	1200274	65259
Guizhou	448406	31859	31909	31612	31262	31837765	27087	26638	26607	26616	501212	27172
Yunnan	537098	38145	38261	38031	37602	42475720	36655	36013	35624	36038	682247	36930
Shaanxi	465541	32796	32678	32268	31717	35187233	30034	29505	28894	29130	549196	29889

Gansu	320059	22590	22562	22262	22002	23912906	20547	20108	19821	19984	377237	20445
Qinghai	65843	4671	4679	4643	4561	5184022	4454	4414	4398	4422	84198	4581
Ningxia	71782	5090	5117	5095	5083	5798346	5045	4961	4969	5062	95349	5210
Xinjiang	243918	17229	17464	17510	17442	19965557	17200	16910	16918	17083	329900	18087

Fixed asset investment price index(%) (Data from China Statistical Yearbook)												
Region	2005	2006	2007	2008	2009	2010	2011	2012	2013	2014	2015	2016
Beijing	100.7	100.4	102.8	107.8	97.1	102.5	105.7	101.3	99.9	100	97.6	99.7
Tianjin	101.2	100.7	102.6	109.2	97.6	102.6	105.7	100	99.5	100.5	99.9	99.4
Hebei	101.9	101.7	103.8	109.6	96.5	103.7	105.5	100.3	99.9	100.2	98	99.4
Shanxi	103	101.5	104.1	113.3	98.1	103.7	105.5	101.2	100.5	99.6	98.2	100
nner Mongoli	103.7	103.3	103.8	108.1	98.5	105.4	106.3	101.6	99.6	99.8	98	99.5
Liaoning	102.8	102.1	104.3	109.1	97	103.3	106.6	101	100	99.7	97.9	99.2
Jilin	102	102.2	103.9	107.3	99.4	102.4	105.6	100.4	100	100.2	97.6	98.7
Heilongjiang	102.2	102.1	104.5	109	97.6	105.2	107.5	100.8	100.1	100	99	99.4
Shanghai	100.8	100.1	103.5	107.9	97	103.8	106.5	99.4	100.2	100.5	97	99.6
Jiangsu	100.9	101.2	104.9	110	97.7	105.1	106.8	98.6	100.5	101.1	96.2	98.8
Zhejiang	100.3	101.5	104.4	109.3	96.7	104.7	107.5	99.2	100	100.6	97.4	99.5
Anhui	101	101.9	105.4	109.4	96	105.4	108.1	101	100.2	100.3	96.9	99.2
Fujian	100.7	102	105.9	105.9	98	103.3	106.2	100.3	100.1	100.4	98.3	100
Jiangxi	100.5	103.2	105.4	110.4	96.1	104.8	108.4	101	100.4	100.1	96.8	100
Shandong	102.9	101.8	104	107.7	96.9	103.6	106.8	100.8	100.4	100.3	97.7	99.1
Henan	101.4	101.6	104.6	109	96.4	103.5	107.4	101	99.9	100	97.6	99.2
Hubei	102.2	101.8	104.1	109.4	98.8	104.7	107.3	101.8	100.5	101	99.4	100.1
Hunan	103.6	103.1	105.8	109.9	99.7	104	107.2	101.7	101.3	101.5	100.4	100.4
Guangdong	101.6	100.7	102.4	108.6	96.7	103	105.5	101.5	101.4	101.5	99	100.3
Guangxi	101.4	101.2	102.3	107.9	97.9	103	106.2	100.6	100.1	101.6	98.8	99.5
Hainan	101.2	101	106.1	113.3	97.7	105.2	106.4	102	99.3	100.6	99.4	100.1
Chongqing	102.3	101.7	105.5	110.2	97.8	102.1	105.9	101.8	100.5	100.3	98.2	98.9
Sichuan	103.9	102.9	104.7	112.5	98.3	102.5	105.2	101	100.4	100.5	97.9	99.8
Guizhou	101.4	101.1	103.5	108.9	100.5	102.7	105.4	101.5	100.9	101.1	98.4	98.6
Yunnan	104.6	101.8	104.2	107.4	98.1	102.7	104.6	101.4	101.1	101	99.1	100.1
Shaanxi	103.7	102.6	104	109.5	99.3	103.6	105.9	102.6	102	101.1	98.8	99.9
Gansu	102.2	104.1	102.8	106.7	101.5	103.5	104.7	102.1	100.4	100.1	97.7	98.7
Qinghai	102.1	102.4	104.2	110.5	100.9	103.8	106.5	102.2	101.5	100.9	98.2	99.6
Ningxia	102.1	101.3	103.2	109	100.2	104.2	107.5	101.5	99.8	100.8	97.5	99.6
Xinjiang	102.8	102.2	104.4	111.2	98	104.6	107.1	100.6	100.5	100.3	98.3	99.9

Per capita disposable income of urban residents(Unit: yuan) (Data from China Statistical Yearbook)												
Region	2005	2006	2007	2008	2009	2010	2011	2012	2013	2014	2015	2016
Beijing	17652.95	19977.52	21988.71	24724.89	26738.48	29072.93	32903.03	36468.75	44563.9	48531.8	52859.2	57275.3
Tianjin	12638.55	14283.09	16357.35	19422.53	21402.01	24292.6	26920.86	29626.41	28979.8	31506	34101.3	37109.6
Hebei	9107.09	10304.56	11690.47	13441.09	14718.25	16263.43	18292.23	20543.44	22226.7	24141.3	26152.2	28249.4
Shanxi	8913.91	10027.7	11564.95	13119.05	13996.55	15647.66	18123.87	20411.71	22258.2	24069.4	25827.7	27352.3
nner Mongoli	9136.79	10357.99	12377.84	14432.55	15849.19	17698.15	20407.57	23150.26	26003.6	28349.6	30594.1	32974.9
Liaoning	9107.55	10369.61	12300.39	14392.69	15761.38	17712.58	20466.84	23222.67	26697	29081.7	31125.7	32876.1
Jilin	8690.62	9775.07	11285.52	12829.45	14006.27	15411.47	17796.57	20208.04	21331.1	23217.8	24900.9	26530.4
Heilongjiang	8272.51	9182.31	10245.28	11581.28	12565.98	13856.51	15696.18	17759.75	20848.4	22609	24202.6	25736.4
Shanghai	18645.03	20667.91	23622.73	26674.9	28837.78	31838.08	36230.48	40188.34	44878.3	48841.4	52961.9	57691.7
Jiangsu	12318.57	14084.26	16378.01	18679.52	20551.72	22944.26	26340.73	29676.97	31585.5	34346.3	37173.5	40151.6
Zhejiang	16293.77	18265.1	20573.82	22726.66	24610.81	27359.02	30970.68	34550.3	37079.7	40392.7	43714.5	47237.2
Anhui	8470.68	9771.05	11473.58	12990.35	14085.74	15788.17	18606.13	21024.21	22789.3	24838.5	26935.8	29156
Fujian	12321.31	13753.28	15506.05	17961.45	19576.83	21781.31	24907.4	28055.24	28173.9	30722.4	33275.3	36014.3
Jiangxi	8619.66	9551.12	11451.69	12866.44	14021.54	15481.12	17494.87	19860.36	22119.7	24309.2	26500.1	28673.3
Shandong	10744.79	12192.24	14264.7	16305.41	17811.04	19945.83	22791.84	25755.19	26882.4	29221.9	31545.3	34012.1
Henan	8667.97	9810.26	11477.05	13231.11	14371.56	15930.26	18194.8	20442.62	21740.7	23672.1	25575.6	27232.9
Hubei	8785.94	9802.65	11485.8	13152.86	14367.48	16058.37	18373.87	20839.59	22667.9	24852.3	27051.5	29385.8
Hunan	9523.97	10504.67	12293.54	13821.16	15084.31	16565.7	18844.05	21318.76	24352	26570.2	28838.1	31283.9
Guangdong	14769.94	16015.58	17699.3	19732.86	21574.72	23897.8	26897.48	30226.71	29537.3	32148.1	34757.2	37684.3
Guangxi	9286.7	9898.75	12200.44	14146.04	15451.48	17063.89	18854.06	21242.8	22689.4	24669	26415.9	28324.4
Hainan	8123.94	9395.13	10996.87	12607.84	13750.85	15581.05	18368.95	20917.71	22411.4	24486.5	26356.4	28453.5
Chongqing	10243.46	11569.74	12590.78	14367.55	15748.67	17532.43	20249.7	22968.14	23058.2	25147.2	27238.8	29610
Sichuan	8385.96	9350.11	11098.28	12633.38	13839.4	15461.16	17899.12	20306.99	22227.5	24234.4	26205.3	28335.3
Guizhou	8151.13	9116.61	10678.4	11758.76	12862.53	14142.74	16495.01	18700.51	20564.9	22548.2	24579.6	26742.6
Yunnan	9265.9	10069.89	11496.11	13250.22	14423.93	16064.54	18575.62	21074.5	22460	24299	26373.2	28610.6
Shaanxi	8272.02	9267.7	10763.34	12857.89	14128.76	15695.21	18245.23	20733.88	22345.9	24365.8	26420.2	28440.1
Gansu	8086.82	8920.59	10012.34	10969.41	11929.78	13188.55	14988.68	17156.89	19873.4	21803.9	23767.1	25693.5
Qinghai	8057.85	9000.35	10276.06	11640.43	12691.85	13854.99	15603.31	17566.28	20352.4	22306.6	24542.3	26757.4
Ningxia	8093.64	9177.26	10859.33	12931.53	14024.7	15344.49	17578.92	19831.41	21475.7	23284.6	25186	27153
Xinjiang	7990.15	8871.27	10313.44	11432.1	12257.52	13643.77	15513.62	17920.68	21091.5	23214	26274.7	28463.4

Per capita consumption expenditure of urban residents(Unit: yuan) (Data from China Statistical Yearbook)												
Region	2005	2006	2007	2008	2009	2010	2011	2012	2013	2014	2015	2016
Beijing	13244.2	14825.41	15330.44	16460.26	17893.3	19934.48	21984.37	24045.86	26274.89	31102.9	36642	38255.5
Tianjin	9653.26	10548.05	12028.88	13422.47	14801.35	16561.77	18424.09	20024.24	21711.86	22343	26229.5	28344.6
Hebei	6699.67	7343.49	8234.97	9086.73	9678.75	10318.32	11609.29	12531.12	13640.58	11931.5	17586.6	19105.9
Shanxi	6342.63	7170.94	8101.84	8806.55	9355.1	9792.65	11354.3	12211.53	13166.19	10863.8	15818.6	16992.8
nner Mongoli	6928.6	7666.61	9281.46	10828.62	12369.87	13994.62	15878.07	17717.1	19249.06	16258.1	21876.5	22744.5
Liaoning	7369.27	7987.49	9429.73	11231.48	12324.58	13280.04	14789.61	16593.6	18029.65	16068	21556.7	24995.9
Jilin	6794.71	7352.64	8560.3	9729.05	10914.44	11679.04	13010.63	14613.53	15932.31	13026	17972.6	19166.4

Heilongjiang	6178.01	6655.43	7519.28	8622.97	9629.6	10683.92	12054.19	12983.55	14161.71	12768.8	17152.1	18145.2
Shanghai	13773.41	14761.75	17255.38	19397.89	20992.35	23200.4	25102.14	26253.47	28155	33064.8	36946.1	39856.8
Jiangsu	8621.82	9628.59	10715.15	11977.55	13153	14357.49	16781.74	18825.28	20371.48	19163.6	24966	26432.9
Zhejiang	12253.74	13348.51	14091.19	15158.3	16683.48	17858.2	20437.45	21545.18	23257.19	22552	28661.3	30067.7
Anhui	6367.67	7294.73	8531.9	9524.04	10233.98	11512.55	13181.46	15011.66	16285.17	11727	17233.5	19606.2
Fujian	8794.41	9807.71	11055.13	12501.12	13450.57	14750.01	16661.05	18593.21	20092.72	17644.5	23520.2	25005.5
Jiangxi	6109.39	6645.54	7810.73	8717.37	9739.99	10618.69	11747.21	12775.65	13850.51	11088.9	16731.8	17695.6
Shandong	7457.31	8468.4	9666.61	11006.61	12012.73	13118.24	14560.67	15778.24	17112.24	13328.9	19853.8	21495.3
Henan	6038.02	6685.18	7826.72	8837.46	9566.99	10838.49	12336.47	13732.96	14821.98	11000.4	17154.3	18087.8
Hubei	6736.56	7397.32	8701.18	9477.51	10294.07	11450.97	13163.77	14495.97	15749.5	12928.3	18192.3	20040
Hunan	7504.99	8169.3	8990.72	9945.52	10828.23	11825.33	13402.87	14608.95	15887.11	13288.7	19501.4	21420
Guangdong	11809.87	12432.22	14336.87	15527.97	16857.5	18489.53	20251.82	22396.35	24133.26	19205.5	25673.1	28613.3
Guangxi	7032.8	6791.95	8151.26	9627.4	10352.38	11490.08	12848.37	14243.98	15417.62	10274.3	16321.2	17268.5
Hainan	5928.79	7126.78	8292.89	9408.48	10086.65	10926.71	12642.75	14456.55	15593.04	12470.6	18448.4	19015.5
Chongqing	8623.29	9398.69	9890.31	11146.8	12144.06	13335.02	14974.49	16573.14	17813.86	13810.6	19742.3	21030.9
Sichuan	6891.27	7524.81	8691.99	9679.14	10860.2	12105.09	13696.3	15049.54	16343.45	12368.4	19276.8	20659.8
Guizhou	6159.29	6848.39	7758.69	8349.21	9048.29	10058.29	11352.88	12585.7	13702.87	9303.4	16914.2	19201.7
Yunnan	6996.9	7379.81	7921.83	9076.61	10201.81	11074.08	12248.03	13883.93	15156.15	9869.5	17675	18622.4
Shaanxi	6656.46	7553.28	8427.06	9772.07	10705.67	11821.88	13782.75	15332.84	16679.69	12203.6	18463.9	19368.9
Gansu	6529.2	6974.21	7875.78	8308.62	8890.79	9895.35	11188.57	12847.05	14020.72	9874.6	17450.9	19539.2
Qinghai	6245.26	6530.11	7512.39	8192.56	8786.52	9613.79	10955.46	12346.29	13539.5	12604.8	19200.6	20853.2
Ningxia	6404.31	7205.57	7817.28	9558.29	10280	11334.43	12896.04	14067.15	15321.1	12484.5	18983.9	20364.2
Xinjiang	6207.52	6730.01	7874.27	8669.36	9327.55	10197.09	11839.4	13891.72	15206.16	11903.7	19414.7	21228.5

Per capita consumption expenditure of rural residents(Unit: yuan) (Data from China Statistical Yearbook)												
Region	2005	2006	2007	2008	2009	2010	2011	2012	2013	2014	2015	2016
Beijing	5315.71	5724.5	6399.27	7284.65	8897.59	9254.77	11077.66	11878.92	13553.2	14535.1	15811.2	17329
Tianjin	3035.96	3341.06	3538.31	3825.43	4273.15	4936.73	6725.42	8336.55	10155	13738.6	14739.4	15912.1
Hebei	2165.72	2495.33	2786.77	3125.55	3349.74	3844.92	4711.16	5364.14	6134.1	8248	9022.8	9798.3
Shanxi	1877.70	2253.25	2682.57	3097.54	3304.76	3663.86	4586.98	5566.19	5812.7	6991.7	7421.2	8028.8
nner Mongoli	2446.17	2771.97	3256.15	3618.11	3968.42	4460.83	5507.72	6381.97	7268.3	9972.2	10637.4	11462.6
Liaoning	2805.94	3066.87	3368.16	3814.03	4254.03	4489.5	5406.41	5998.39	7159	7800.7	8872.8	9953.1
Jilin	2305.98	2700.66	3065.44	3443.24	3902.9	4147.36	5305.75	6186.17	7379.7	8139.8	8783.3	9521.4
Heilongjiang	2544.65	2618.19	3117.44	3844.73	4241.27	4391.17	5333.61	5718.05	6813.6	7830	8391.5	9423.8
Shanghai	7277.94	8006	8844.88	9119.67	9804.37	10210.46	11049.32	11971.5	14234.7	14820.1	16152.3	17070.8
Jiangsu	3567.11	4135.21	4786.15	5328.37	5804.45	6542.87	8094.57	9138.18	9909.8	11820.3	12882.5	14428.2
Zhejiang	5432.95	6057.16	6801.6	7534.09	7731.7	8928.89	9965.08	10652.73	11760.2	14497.8	16107.7	17358.9
Anhui	2196.23	2420.94	2754.04	3284.11	3655.02	4013.31	4957.29	5555.99	5724.5	7980.8	8975.2	10287.3
Fujian	3292.63	3591.4	4053.47	4661.94	5015.72	5498.33	6540.85	7401.92	8151.2	11055.9	11960.8	12910.8
Jiangxi	2483.70	2676.6	2994.49	3309.21	3532.66	3911.61	4659.87	5129.47	5653.6	7548.3	8485.6	9128.3
Shandong	2735.77	3143.8	3621.57	4077.05	4417.18	4807.18	5900.57	6775.95	7392.7	7962.2	8747.6	9518.9
Henan	1891.57	2229.28	2676.41	3044.21	3388.47	3682.21	4319.95	5032.14	5627.7	7277.2	7887.4	8586.6
Hubei	2430.19	2732.46	3090	3652.57	3725.24	4090.78	5010.74	5726.73	6279.5	8680.9	9803.1	10938.3
Hunan	2756.43	3013.32	3377.38	3804.97	4020.87	4310.37	5179.36	5870.12	6609.5	9024.8	9690.6	10629.9
Guangdong	3707.73	3885.97	4202.32	4872.46	5019.81	5515.58	6725.55	7458.56	8343.5	10043.2	11103	12414.8
Guangxi	2349.60	2413.93	2747.47	2985.03	3231.14	3455.29	4210.89	4933.58	5205.6	6675.1	7582	8351.2
Hainan	1969.09	2232.19	2556.56	2883.1	3088.56	3446.24	4166.13	4776.3	5465.6	7029	8210.3	8921.2
Chongqing	2142.12	2205.21	2526.7	2884.92	3142.14	3624.62	4502.06	5018.64	5796.4	7982.6	8937.7	9954.4
Sichuan	2274.17	2395.04	2747.27	3127.94	4141.4	3897.53	4675.47	5366.71	6308.5	8301.1	9250.6	10191.6
Guizhou	1552.39	1627.07	1913.71	2165.7	2421.95	2852.48	3455.78	3901.71	4740.2	5970.3	6644.9	7533.3
Yunnan	1789.00	2195.64	2637.18	2990.61	2924.85	3398.33	3999.87	4561.33	4743.6	6030.3	6830.1	7330.5
Shaanxi	1896.48	2181	2559.59	2979.37	3349.23	3793.8	4491.71	5114.68	5724.2	7252.4	7900.7	8567.7
Gansu	1819.58	1855.49	2017.21	2400.95	2766.45	2941.99	3664.91	4146.24	4849.6	6147.8	6829.8	7487
Qinghai	1976.03	2178.95	2446.5	2896.62	3209.41	3774.5	4536.81	5338.91	6060.2	8235.1	8566.5	9222.2
Ningxia	2094.48	2246.97	2528.76	3094.86	3347.94	4013.17	4726.64	5351.36	6489.7	7676.5	8414.9	9138.4
Xinjiang	1924.41	2032.36	2350.58	2691.79	2950.63	3457.88	4397.82	5301.25	6119.1	7365.3	7697.9	8277

Share of secondary industry value added in regional GDP(%) (Data from China Statistical Yearbook)												
Region	2005	2006	2007	2008	2009	2010	2011	2012	2013	2014	2015	2016
Beijing	29.5	27.8	26.8	25.7	23.5	24	23.1	22.7	22.3	21.3	19.7	19.3
Tianjin	55.5	57.1	57.3	60.1	53	52.5	52.4	51.7	50.6	49.2	46.6	42.3
Hebei	51.8	52.4	52.8	54.2	52	52.5	53.5	52.7	52.2	51	48.3	47.6
Shanxi	56.3	57.8	60	61.5	54.3	56.9	59	55.6	53.9	49.3	40.7	38.5
nner Mongoli	45.5	48.6	51.8	55	52.5	54.6	56	55.4	54	51.3	50.5	47.2
Liaoning	49.4	51.1	53.1	55.8	52	54.1	54.7	53.2	52.7	50.2	45.5	38.7
Jilin	43.6	44.8	46.8	47.7	48.7	52	53.1	53.4	52.8	52.8	49.8	47.4
Heilongjiang	53.9	54.4	52.3	52.5	47.3	50.2	50.3	44.1	41.1	36.9	31.8	28.6
Shanghai	48.6	48.5	46.6	45.5	39.9	42.1	41.3	38.9	37.2	34.7	31.8	29.8
Jiangsu	56.6	56.6	55.6	55	53.9	52.5	51.3	50.2	49.2	47.4	45.7	44.7
Zhejiang	53.4	54	54	53.9	51.8	51.6	51.2	50	49.1	47.7	46	44.9
Anhui	41.3	43.1	44.7	46.6	48.7	52.1	54.3	54.6	54.6	53.1	49.7	48.4
Fujian	48.7	49.1	49.2	50	49.1	51	51.6	51.7	52	52	50.3	48.9
Jiangxi	47.3	49.7	51.7	52.7	51.2	54.2	54.6	53.6	53.5	52.5	50.3	47.7
Shandong	57.4	57.7	56.9	57	55.8	54.2	52.9	51.5	50.1	48.4	46.8	46.1
Henan	52.1	53.8	55.2	56.9	56.5	57.3	57.3	56.3	55.4	51	48.4	47.6
Hubei	43.1	44.4	43	43.8	46.6	48.6	50	50.3	49.3	46.9	45.7	44.9
Hunan	39.9	41.6	42.6	44.2	43.5	45.8	47.6	47.4	47	46.2	44.3	42.3
Guangdong	50.7	51.3	51.3	51.6	49.2	50	49.7	48.5	47.3	46.3	44.8	43.4
Guangxi	37.1	38.9	40.7	42.4	43.6	47.1	48.4	47.9	47.7	46.7	45.9	45.2
Hainan	24.6	27.4	29.8	29.8	26.8	27.7	28.3	28.2	27.7	25	23.7	22.4
Chongqing	41	43	45.9	47.7	52.8	55	55.4	52.4	50.5	45.8	45	44.5

Sichuan	41.5	43.7	44.2	46.3	47.4	50.5	52.5	51.7	51.7	48.9	44.1	40.8
Guizhou	41.8	43	41.9	42.3	37.7	39.1	38.5	39.1	40.5	41.6	39.5	39.7
Yunnan	41.2	42.8	43.3	43	41.9	44.6	42.5	42.9	42	41.2	39.8	38.5
Shaanxi	50.3	53.9	54.2	56.1	51.9	53.8	55.4	55.9	55.5	54.1	50.4	48.9
Gansu	43.4	45.8	47.3	46.3	45.1	48.2	47.4	46	45	42.8	36.7	34.9
Qinghai	48.7	51.6	53.3	55.1	53.2	55.1	58.4	57.7	57.3	53.6	49.9	48.6
Ningxia	46.4	49.2	50.8	52.9	48.9	49	50.2	49.5	49.3	48.7	47.4	47
Xinjiang	44.7	48	46.8	49.6	45.1	47.7	48.8	46.4	45	42.6	38.6	37.8

GDP growth index(%) (Data from China Statistical Yearbook)												
Region	2005	2006	2007	2008	2009	2010	2011	2012	2013	2014	2015	2016
Beijing	111.8	112.8	113.3	109	110.2	110.3	108.1	107.7	107.7	107.3	106.9	106.8
Tianjin	114.7	114.5	115.2	116.5	116.5	117.4	116.4	113.8	112.5	110	109.3	109.1
Hebei	113.4	113.4	112.8	110.1	110	112.2	111.3	109.6	108.2	106.5	106.8	106.8
Shanxi	112.6	111.8	114.4	108.3	105.4	113.9	113	110.1	108.9	104.9	103.1	104.5
nner Mongoli	123.8	118.7	119.1	117.2	116.9	115	114.3	111.5	109	107.8	107.7	107.2
Liaoning	112.3	113.8	114.5	113.1	113.1	114.2	112.2	109.5	108.7	105.8	103	97.5
Jilin	112.1	115	116.1	116	113.6	113.8	113.8	112	108.3	106.5	106.3	106.9
Heilongjiang	111.6	112.1	112	111.8	111.4	112.7	112.3	110	108	105.6	105.7	106.1
Shanghai	111.1	112	114.3	109.7	108.2	110.3	108.2	107.5	107.7	107	106.9	106.9
Jiangsu	114.5	114.9	114.9	112.3	112.4	112.7	111	110.1	109.6	108.7	108.5	107.8
Zhejiang	112.8	113.9	114.7	110.1	108.9	111.9	109	108	108.2	107.6	108	107.6
Anhui	111.6	112.8	113.9	112.7	112.9	114.6	113.5	112.1	110.4	109.2	108.7	108.7
Fujian	111.6	114.8	115.2	113	112.3	113.9	112.3	111.4	111	109.9	109	108.4
Jiangxi	112.8	112.3	113	112.6	113.1	114	112.5	111	110.1	109.7	109.1	109
Shandong	115.2	114.8	114.3	112.1	112.2	112.3	110.9	109.8	109.6	108.7	108	107.6
Henan	114.2	114.4	114.6	112.1	110.9	112.5	111.9	110.1	109	108.9	108.3	108.1
Hubei	112.1	113.2	114.5	113.4	113.5	114.8	113.8	111.3	110.1	109.7	108.9	108.1
Hunan	111.6	112.2	114.5	112.8	113.7	114.6	112.8	111.3	110.1	109.5	108.5	108
Guangdong	113.8	114.6	114.7	110.1	109.7	112.4	110	108.2	108.5	107.8	108	107.5
Guangxi	113.2	113.6	115.1	112.8	113.9	114.2	112.3	111.3	110.2	108.5	108.1	107.3
Hainan	110.2	112.5	114.8	109.8	111.7	116	112	109.1	109.9	108.5	107.8	107.5
Chongqing	111.5	112.2	115.6	114.3	114.9	117.1	116.4	113.6	112.3	110.9	111	110.7
Sichuan	112.6	113.3	114.2	109.5	114.5	115.1	115	112.6	110	108.5	107.9	107.8
Guizhou	111.6	111.6	113.7	110.2	111.4	112.8	115	113.6	112.5	110.8	110.7	110.5
Yunnan	109.0	111.9	112.5	111	112.1	112.3	113.7	113	112.1	108.1	108.7	108.7
Shaanxi	112.6	112.8	114.6	115.6	113.6	114.6	113.9	112.9	111	109.7	107.9	107.6
Gansu	111.8	111.5	112.3	110.1	110.3	111.8	112.5	112.6	110.8	108.9	108.1	107.6
Qinghai	112.2	112.2	112.5	112.7	110.1	115.3	113.5	112.3	110.8	109.2	108.2	108
Ningxia	110.9	112.7	112.7	112.2	111.9	113.5	112.1	111.5	109.8	108	108	108.1
Xinjiang	110.9	111	112.2	111	108.1	110.6	112	112	111	110	108.8	107.6

Total wastewater discharge(Unit:10000 tons)(Data from China Statistical Yearbook, China Environmental Statistical Yearbook, provincial and municipal statistical												
Region	2005	2006	2007	2008	2009	2010	2011	2012	2013	2014	2015	2016
Beijing	12813	10170	9134	8367	8713	8198	8633	9190	9486	9174	8978	8515.44
Tianjin	30081	22978	21444	20433	19441	19680	19795	19117	18692	19011	18973	18022
Hebei	124533	130340	123537	121172	110058	114232	118505	122645	109876	108562	94110	67790
Shanxi	32099	44091	41140	41150	39720	49881	39665	48108	47795	49250	41356	28513.4
nner Mongoli	24967	27823	25021	29167	28616	39536	39409	33618	36986	39325	35753	24200
Liaoning	105072	94724	95197	83073	75159	71521	90457	87168	78286	90631	83140	57639.21
Jilin	41189	39321	39666	38353	37563	38656	41884	44842	42656	42192	38772	19237.55
Heilongjiang	45158	44801	38388	38910	34188	38921	44072	58355	47796	41984	36410	23935
Shanghai	51097	48336	47570	41871	41192	36696	44626	46359	45426	43939	46939	36600
Jiangsu	296318	287181	268762	259999	256160	263760	246298	236094	220559	204890	206427	179400
Zhejiang	192426	199593	201211	200488	203442	217426	182240	175416	163674	149380	147353	129913
Anhui	63487	70119	73556	67007	73441	70971	70720	67175	70972	69580	71436	49625
Fujian	130939	127583	136408	139997	142747	124168	177186	106319	104658	102052	90741	68872.15
Jiangxi	53972	64074	71410	68681	67192	72526	71196	67871	68230	64856	76412	85527
Shandong	139071	144365	166574	176977	182673	208257	187245	183634	181179	180022	185493	160580
Henan	123476	130158	134344	133144	140325	150406	138654	137356	130789	128048	129809	69500
Hubei	92432	91146	91001	93687	91324	94593	104434	91609	84993	81657	80817	49090
Hunan	122440	100024	100113	92340	96396	95605	97197	97133	92311	82271	76888	48692.86
Guangdong	231568	234713	246331	213314	188844	187031	178626	186126	170463	177554	161455	132000
Guangxi	145609	128932	183981	205745	161596	165211	101234	110671	89508	72936	63253	32554
Hainan	7428	7351	5960	5991	7031	5782	6820	7465	6744	7956	6879	5061.8784
Chongqing	84885	86496	69003	67027	65684	45180	33954	30611	33451	34968	35524	25874
Sichuan	122590	115348	114687	108700	105910	93444	80420	69984	64864	67577	71647	64071.408
Guizhou	14850	13928	12101	11695	13478	14130	20626	23399	22898	32674	29174	16400
Yunnan	32928	34286	35352	32996	32375	30926	47228	42811	41844	40443	45933	48800
Shaanxi	42819	40479	48523	48477	49137	45487	40806	38037	34871	36163	37730	28416.76
Gansu	16798	16570	15856	16405	16364	15352	19720	19188	20171	19742	18760	13022.13
Qinghai	7619	7168	7318	7098	8404	9031	8677	8917	8395	8214	8546	6732.0594
Ningxia	21411	18500	21089	20448	21542	21977	19285	16548	15708	15147	16443	12194
Xinjiang	20052	20558	20960	22875	24201	25413	28769	29738	34718	32799	28402	15900

Production of industrial solid waste(Unit:10000 tons)(Data from China Statistical Yearbook, China Environmental Statistical Yearbook,, provincial and municipal												
Region	2005	2006	2007	2008	2009	2010	2011	2012	2013	2014	2015	2016
Beijing	1238	1356	1274.84	1157	1242.44	1269	1126	1104	1044	1021	709.86	629
Tianjin	1123	1292	1399.40	1479	1515.67	1862	1752	1820	1592	1735	1546	1490
Hebei	16279	14229	18688.29	19769	21975.81	31688	45129	45576	43289	41928	35372	33236

Shanxi	11183	11817	13819.14	16213	14742.85	18270	27556	29031	30520	30199	31794	28845
nner Mongoli	7363	8710	10972.78	10622	12108.32	16996	23584	24226	20081	23191	26669	24762
Liaoning	10242	13013	14341.81	15841	17221.41	17273	28270	27280	26759	28666	32434	22822
Jilin	2457	2802	3112.56	3415	3940.52	4642	5379	4731	4591	4944	5385	4006
Heilongjiang	3210	3914	4130.13	4472	5274.72	5405	6017	6313	6094	6312	7495	6940
Shanghai	1964	2063	2165.40	2347	2254.59	2448	2442	2199	2054	1925	1868	1680
Jiangsu	5757	7195	7354.22	7724	8027.81	9064	10475	10224	10856	10925	10701	11649
Zhejiang	2514	3096	3613.45	3785	3909.66	4268	4446	4461	4300	4542	4486	4263
Anhui	4196	5028	5960.35	7569	8470.83	9158	11473	12022	11937	12000	13059	12653
Fujian	3773	4238	4814.86	5371	6348.91	7487	4415	7720	8535	4835	4956	4449
Jiangxi	7007	7393	7777.28	8190	8898.18	9407	11372	11134	11518	10821	10777	12665
Shandong	9175	11011	11934.73	12988	14137.95	16038	19533	18343	18172	19199	19798	22510
Henan	6178	7464	8850.58	9557	10785.82	10714	14574	15250	16270	15917	14722	14256
Hubei	3692	4315	4682.66	5014	5561.45	6813	7596	7611	8181	8006	7750	8193
Hunan	3366	3688	4559.73	4520	5092.79	5773	8487	8116	7806	6934	7126	5320
Guangdong	2896	3057	3852.43	4833	4740.85	5456	5849	5965	5912	5665	5609	5610
Guangxi	3489	3894	4543.64	5417	5693.10	6232	7438	7964	7676	8038	6977	6938
Hainan	127	147	157.93	220	200.86	212	421	386	415	515	422	330
Chongqing	1777	1764	2086.84	2311	2551.85	2837	3299	3115	3162	3068	2828	2344
Sichuan	6421	7600	9653.82	9237	8596.86	11239	12684	13187	14007	14246	12316	11765
Guizhou	4854	5827	5988.58	5844	7317.37	8188	7598	7835	8194	7394	7055	7753
Yunnan	4661	5972	7097.52	7986	8672.83	9392	17335	16038	16040	14481	14109	13122
Shaanxi	4588	4794	5480.02	6121	5546.67	6892	7118	7215	7491	8682	9330	8648
Gansu	2249	2591	3001.46	3199	3150.21	3745	6524	6671	5907	6141	5824	5091
Qinghai	649	882	1129.27	1337	1347.62	1783	12017	12301	12377	12423	14868	14669
Ningxia	719	799	1045.74	1143	1398.25	2465	3344	2961	3277	3694	3430	3618
Xinjiang	1295	1581	2136.64	2438	3206.08	3914	5219	7880	9283	7790	7263	8530

Total energy consumption(Unit: 10,000 tons of standard coal) (Data from China Energy Statistical Yearbook, provincial and municipal statistical yearbooks)												
Region	2005	2006	2007	2008	2009	2010	2011	2012	2013	2014	2015	2016
Beijing	5522	5904	6285	6327	6570	6954	6995	7178	6724	6831	6853	6962
Tianjin	4085	4500	4943	5364	5874	6818	7598	8208	7882	8145	8260	8245
Hebei	19836	21794	23585	24322	25419	27531	29498	30250	29664	29320	29395	29794
Shanxi	12750	14098	15601	15675	15576	16808	18315	19336	19761	19863	19384	19401
nner Mongoli	9666	11221	12777	14100	15344	16820	18737	19786	17681	18309	18927	19457
Liaoning	13611	14987	16544	17801	19112	20947	22712	23526	21721	21803	21667	21031
Jilin	5258.5	5871.5	6465.9	7100.1	7553.4	8172.8	8886.9	9028.3	8645.4	8559.8	8141.9	8014.1
Heilongjiang	8050	8731	9377	9979	10467	11234	12119	12758	11853	11955	12126	12280
Shanghai	8225	8876	9670	10207	10367	11201	11270	11362	11346	11085	11387	11712
Jiangsu	17167	19041	20948	22232	23709	25774	27589	28850	29205	29863	30235	31054
Zhejiang	12032	13219	14524	15107	15567	16865	17827	18076	18640	18826	19610	20276
Anhui	6506	7069	7739	8325	8896	9707	10570	11358	11696	12011	12332	12695
Fujian	6142	6828	7587	8254	8916	9809	10653	11185	11190	12110	12180	12358
Jiangxi	4286	4660	5053	5383	5813	6355	6928	7233	7583	8055	8440	8747
Shandong	24162	26759	29177	30570	32420	34808	37132	38899	35358	36511	37945	38723
Henan	14625	16232	17838	18976	19751	21438	23062	23647	21909	22890	23161	23117
Hubei	10082	11049	12143	12845	13708	15138	16579	17675	15703	16320	16404	16850
Hunan	9709	10581	11629	12355	13331	14880	16161	16744	14919	15317	15469	15804
Guangdong	17921	19971	22217	23476	24654	26908	28480	29144	28480	29593	30145	31241
Guangxi	4869	5390	5997	6497	7075	7919	8591	9155	9100	9515	9761	10092
Hainan	822	920	1057	1135	1233	1359	1601	1688	1720	1820	1938	2006
Chongqing	4943	5368	5947	6472	7030	7856	8792	9278	8049	8593	8934	9204
Sichuan	11816	12986	14214	15145	16322	17892	19696	20575	19212	19879	19888	20362
Guizhou	5641	6172	6800	7084	7566	8175	9068	9878	9299	9709	9948	10227
Yunnan	6024	6621	7133	7511	8032	8674	9540	10434	10072	10455	10357	10656
Shaanxi	5571	6129	6775	7417	8044	8882	9761	10626	10610	11222	11716	12120
Gansu	4368	4743	5109	5346	5482	5923	6496	7007	7287	7521	7523	7334
Qinghai	1670	1903	2095	2279	2348	2568	3189	3524	3768	3992	4134	4111
Ningxia	2499.6	2789.1	3033	3182.8	3338.9	3628.1	4254.1	4496.7	4780.5	4946.1	5404.7	5591.8
Xinjiang	5506	6047	6576	7069	7526	8290	9927	11831	13632	14926	15651	16302

Total exports(Unit: USD 10000)(According to the location of the operating unit) (Data from China Statistical Yearbook)												
Region	2005	2006	2007	2008	2009	2010	2011	2012	2013	2014	2015	2016
Beijing	3086590	3795398	4892639.4	5749960.9	4837932	5543621.1	5899715	5963209	6309756	6233842	5466682	5202284
Tianjin	2738088	3349078	3807405.2	4210299.1	2989271.9	3748482.6	4448194	4831256	4900494	5259066	5116293	4427869
Hebei	1092430	1283400	1700040.6	2400412.1	1568890.2	2255644.3	2856985	2959820	3096061	3571020	3293276	3057554
Shanxi	352849	413963	653249.2	925311.6	283745.5	470281.5	542512	701604	799557	894087	842077	993219
nner Mongoli	177362	214050	294439.4	359185	231547.6	333442.6	468697	397016	409256	639355	565001	439599
Liaoning	2343832	2831942	3532408.9	4206949.7	3341492.8	4309871.1	5104236	5795905	6452201	5874518	5071098	4306277
Jilin	246616	299665	385705.6	477162.9	312493.5	447584.9	499772	598268	673891	577759	461375	420201
Heilongjiang	606944	843595	1225712.2	1680624.4	1008212.7	1628078.6	1767299	1443517	1623173	1733524	803541	503553
Shanghai	9071752	11358927	14384611	16914514	14179603	18071398	20967384	20673017	20418003	21013386	19591321	18335213
Jiangsu	12296671	16040962	20360978	23802941	19919919	27053869	31259006	32852352	32880175	34183250	33864478	31905309
Zhejiang	7680245	10089056	12826397	15429623	13301295	18046478	21634949	22451714	24874624	27332705	27633211	26786375
Anhui	518850	683775	881373.4	1136411.1	888648.7	1241288.8	1708264	2674850	2825131	3148537	3227017	2844668
Fujian	3484187	4126174	4993757.3	5699184.3	5331911	7149312.8	9283778	9783259	10647442	11345229	11268011	10367799
Jiangxi	243934	375302	544458.7	772665.6	736848.8	1341606.3	2187606	2511279	2816665	3202532	3311674	2979840
Shandong	4612289	5859834	7511010.5	9319478.5	7949070.6	10422560	12571257	12870921	13419013	14470865	14392568	13709609
Henan	508753	663440	837491.6	1071889.7	734537.6	1052937.2	1923991	2967645	3598710	3938303	4306137	4280551
Hubei	442868	626063	817293.9	1170890.9	997879.6	1444179.5	1953460	1939850	2283621	2664242	2921182	2603934
Hunan	374714	509182	651539.7	841288.4	549203.4	795598.9	990380	1260220	1482120	1994300	1913709	1769254

Guangdong	23815883	30194643	36931609	40566447	35895489	45319116	53192657	57405077	63636385	64608701	64317208	59860199
Guangxi	287663	359297	510915.7	734744.4	837537	960307.3	1245776	1546775	1869326	2432743	2793398	2292641
Hainan	102254	137562	136445.8	158719.8	130863.2	232033	254162	313610	370649	441674	374304	212580
Chongqing	252058	335102	450720.7	572204.9	428007.1	748893.7	1983165	3856758	4679590	6340080	5518683	4065438
Sichuan	470161	662412	860595.9	1313248.5	1416944.7	1884062.5	2902729	3846907	4194906	4483913	3309290	2794762
Guizhou	85894	103843	146546.6	190077.5	135661.2	192018	298509	495223	688598	939726	994862	474279
Yunnan	264173	339149	476827.9	498441.3	451325.2	760576.7	947245	1001737	1567138	1878747	1661563	1149031
Shaanxi	307689	362961	467524.9	538081.7	398814.9	620821.5	703503	865226	1022566	1392980	1478876	1583756
Gansu	109100	150960	165865.7	160135.4	73551.2	163778.5	215878	357355	467732	532948	581181	406256
Qinghai	32320	53420	38591.3	41910	25187.6	46619.7	66182	72876	84726	112790	164197	137006
Ningxia	68742	94262	108567.4	125836.8	74293	117000.2	159943	164112	255216	430285	296311	248675
Xinjiang	503892	713923	1150217	1929910	1093456.3	1296865.2	1682572	1934565	2226774	2348076	1749608	1558217

Average annual exchange rate of RMB(RMB/USD) (Data from China Statistical Yearbook)												
Region	2005	2006	2007	2008	2009	2010	2011	2012	2013	2014	2015	2016
Beijing	8.19	7.97	7.604	6.9451	6.831	6.7695	6.4588	6.3125	6.1932	6.1428	6.2284	6.6423
Tianjin	8.19	7.97	7.604	6.9451	6.831	6.7695	6.4588	6.3125	6.1932	6.1428	6.2284	6.6423
Hebei	8.19	7.97	7.604	6.9451	6.831	6.7695	6.4588	6.3125	6.1932	6.1428	6.2284	6.6423
Shanxi	8.19	7.97	7.604	6.9451	6.831	6.7695	6.4588	6.3125	6.1932	6.1428	6.2284	6.6423
nner Mongoli	8.19	7.97	7.604	6.9451	6.831	6.7695	6.4588	6.3125	6.1932	6.1428	6.2284	6.6423
Liaoning	8.19	7.97	7.604	6.9451	6.831	6.7695	6.4588	6.3125	6.1932	6.1428	6.2284	6.6423
Jilin	8.19	7.97	7.604	6.9451	6.831	6.7695	6.4588	6.3125	6.1932	6.1428	6.2284	6.6423
Heilongjiang	8.19	7.97	7.604	6.9451	6.831	6.7695	6.4588	6.3125	6.1932	6.1428	6.2284	6.6423
Shanghai	8.19	7.97	7.604	6.9451	6.831	6.7695	6.4588	6.3125	6.1932	6.1428	6.2284	6.6423
Jiangsu	8.19	7.97	7.604	6.9451	6.831	6.7695	6.4588	6.3125	6.1932	6.1428	6.2284	6.6423
Zhejiang	8.19	7.97	7.604	6.9451	6.831	6.7695	6.4588	6.3125	6.1932	6.1428	6.2284	6.6423
Anhui	8.19	7.97	7.604	6.9451	6.831	6.7695	6.4588	6.3125	6.1932	6.1428	6.2284	6.6423
Fujian	8.19	7.97	7.604	6.9451	6.831	6.7695	6.4588	6.3125	6.1932	6.1428	6.2284	6.6423
Jiangxi	8.19	7.97	7.604	6.9451	6.831	6.7695	6.4588	6.3125	6.1932	6.1428	6.2284	6.6423
Shandong	8.19	7.97	7.604	6.9451	6.831	6.7695	6.4588	6.3125	6.1932	6.1428	6.2284	6.6423
Henan	8.19	7.97	7.604	6.9451	6.831	6.7695	6.4588	6.3125	6.1932	6.1428	6.2284	6.6423
Hubei	8.19	7.97	7.604	6.9451	6.831	6.7695	6.4588	6.3125	6.1932	6.1428	6.2284	6.6423
Hunan	8.19	7.97	7.604	6.9451	6.831	6.7695	6.4588	6.3125	6.1932	6.1428	6.2284	6.6423
Guangdong	8.19	7.97	7.604	6.9451	6.831	6.7695	6.4588	6.3125	6.1932	6.1428	6.2284	6.6423
Guangxi	8.19	7.97	7.604	6.9451	6.831	6.7695	6.4588	6.3125	6.1932	6.1428	6.2284	6.6423
Hainan	8.19	7.97	7.604	6.9451	6.831	6.7695	6.4588	6.3125	6.1932	6.1428	6.2284	6.6423
Chongqing	8.19	7.97	7.604	6.9451	6.831	6.7695	6.4588	6.3125	6.1932	6.1428	6.2284	6.6423
Sichuan	8.19	7.97	7.604	6.9451	6.831	6.7695	6.4588	6.3125	6.1932	6.1428	6.2284	6.6423
Guizhou	8.19	7.97	7.604	6.9451	6.831	6.7695	6.4588	6.3125	6.1932	6.1428	6.2284	6.6423
Yunnan	8.19	7.97	7.604	6.9451	6.831	6.7695	6.4588	6.3125	6.1932	6.1428	6.2284	6.6423
Shaanxi	8.19	7.97	7.604	6.9451	6.831	6.7695	6.4588	6.3125	6.1932	6.1428	6.2284	6.6423
Gansu	8.19	7.97	7.604	6.9451	6.831	6.7695	6.4588	6.3125	6.1932	6.1428	6.2284	6.6423
Qinghai	8.19	7.97	7.604	6.9451	6.831	6.7695	6.4588	6.3125	6.1932	6.1428	6.2284	6.6423
Ningxia	8.19	7.97	7.604	6.9451	6.831	6.7695	6.4588	6.3125	6.1932	6.1428	6.2284	6.6423
Xinjiang	8.19	7.97	7.604	6.9451	6.831	6.7695	6.4588	6.3125	6.1932	6.1428	6.2284	6.6423

of foreign direct investment(Unit: USD 10000) (Data from China Statistical Yearbook, New China 60 years of statistical data compilation, the provincial and municipal sta												
Region	2005	2006	2007	2008	2009	2010	2011	2012	2013	2014	2015	2016
Beijing	352638	455191	506572	608172	612094	636358	705447	804160	852418	904085	1299635	1302858
Tianjin	332885	413077	527776	741978	901985	1084872	1305602	1501633	1682897	1886676	2113444	1010046
Hebei	191256	201434	241621	341868	359824	383074	468095	580486	644720	637196	617750	735388
Shanxi	27516	47199	134283	102282	49315	71421	207278	250379	280667	295186	286985	233242
nner Mongoli	118577	174066	214889	265074	298385	338456	383827	394319	464456	397748	336629	396672
Liaoning	359042	598554	909673	1201925	1544390	2075010	2426739	2679315	2903996	2742335	518516	299902
Jilin	66115	76064	88495	99331	113977	128042	148125	164866	181949	196643	212747	227449
Heilongjiang	152202	174901	216908	265642	250900	275851	345694	399140	464232	515551	554509	589647
Shanghai	685000	710700	792000	1008400	1054000	1112100	1260055	1518453	1677958	1816593	1845923	1851378
Jiangsu	1318339	1743140	2189206	2512001	2532298	2849777	3213173	3575956	3325922	2817416	2427469	2454296
Zhejiang	772271	888935	1036576	1007294	993974	1100175	1166601	1306926	1415898	1579725	1696024	1757748
Anhui	68845	139354	299892	348988	388416	501446	662887	863811	1068772	1233978	1361945	1476712
Fujian	260775	322047	406058	567171	573747	580279	620111	633774	667896	711499	768339	819465
Jiangxi	242258	280657	310358	360368	402354	510084	605881	682431	755096	845074	947321	1044056
Shandong	897072	1000069	1101159	820246	801007	916833	1116022	1235267	1405315	1519511	1630090	1682556
Henan	122960	184526	306162	403266	479858	624670	1008209	1211777	1345659	1492688	1608637	1699312
Hubei	218475	244853	276622	324481	365766	405015	465503	566591	688847	792792	894801	1012889
Hunan	207235	259335	327051	400515	459787	518441	615031	728034	870482	1026585	1156441	1285208
Guangdong	1236391	1451065	1712603	1916703	1953460	2026098	2179836	2354911	2495210	2687144	2687546	2334921
Guangxi	37866	44740	68396	97119	103533	91200	101381	74853	70008	100119	172208	88845
Hainan	68401	74878	112001	128337	93806	151213	152299	164119	181060	188858	200567	213134
Chongqing	51575	69595	108534	272913	404383	636956	1057862	1057661	1059715	1062946	1076505	1134184
Sichuan	88686	120819	149322	308842	412933	701299	1102733	1055054	1057481	1065328	1043681	854381
Guizhou	19568	18413	15333	17379	18027	34026	71674	109805	157415	213053	262747	321631
Yunnan	17352	30234	39453	77688	91010	132900	173800	218900	251500	270600	299200	86700
Shaanxi	62839	92489	119516	136954	151053	182006	235483	293609	367800	417557	462118	501178
Gansu	2044	2954	11802	12842	13383	13521	7024	6110	7129	10032	11036	11588
Qinghai	26600	27500	31000	22000	21500	21930	16893	20578	9372	5010	5500	1495
Ningxia	14107	13794	16968	12073	14208	23175	34226	34809	20384	14304	18639	25363
Xinjiang	4749	10366	12484	18984	21570	23742	33485	40795	48102	41700	45250	40076

bound overnight tourists(Unit: 10000 people)(Data from China Statistical Yearbook, New China 60 years of statistical data compilation, the provincial and municipal statisti

Region	2005	2006	2007	2008	2009	2010	2011	2012	2013	2014	2015	2016
Beijing	362.92	390.29	435.48	379.04	412.51	490.07	520.4	500.86	450.13	427.45	419.96	416.53
Tianjin	74.01	88.05	103.23	122.04	141.02	166.07	73.06	73.75	75.86	76.63	78.48	82.43
Hebei	62.65	72.48	81.76	75.02	84.22	97.74	114.14	129.32	84.27	75.61	76.64	83.79
Shanxi	42.15	57.37	73.79	93.93	106.78	130.29	155.32	189.18	53.84	56.56	59.38	62.98
nner Mongoli	100.16	123.24	149.45	154.93	128.96	142.8	151.52	159.17	161.61	167.31	160.78	177.91
Liaoning	130.2	161.29	200.09	241.87	293.2	361.8	405.33	473.13	256.04	260.7	264.01	273.67
Jilin	37.32	43.48	54.36	61.73	68.05	82.01	99.32	118.27	124.3	130.63	148.1	161.95
Heilongjiang	82.15	106.37	141.42	200.61	142.51	172.42	206.52	207.62	152.86	141.72	83.47	95.7
Shanghai	444.54	464.63	520.10	526.47	533.39	733.72	668.61	651.23	614.09	639.62	653.59	690.43
Jiangsu	378.3	445.19	512.55	544.30	556.83	653.55	737.33	791.54	288.03	297.1	305.01	329.77
Zhejiang	348.05	426.82	511.18	539.67	570.64	684.71	773.69	865.93	337.57	370.88	459.02	525.59
Anhui	63.29	80.37	106.43	132.09	156.16	198.42	262.87	331.47	271.95	280.18	291.12	313.43
Fujian	197.39	229.67	268.75	293.19	312.03	368.14	427.42	493.67	294.02	318.9	332.71	611.48
Jiangxi	37.25	49.72	66.47	80.21	96.43	113.97	135.83	156.18	123.89	147.67	155.88	164.83
Shandong	155.11	193.13	249.64	253.67	310.04	366.79	424.23	469.91	285.98	300.19	312.22	328.82
Henan	60.05	75.74	88.09	104.36	125.85	146.84	168.29	190.77	127.38	124.76	135.3	149.93
Hubei	82.57	105.57	131.82	118.75	133.46	181.74	213.52	264.72	267.96	277.07	311.76	337.56
Hunan	71.98	97.08	120.57	111.02	130.87	189.87	227.63	224.55	230.66	219.55	226.05	240.81
Guangdong	1896.99	2089.71	2460.87	2567.97	2747.8	3140.93	3331.63	3489.43	3397.9	3355.43	3450.35	3507.21
Guangxi	147.71	170.77	205.52	201.02	209.85	250.24	302.79	350.27	281.74	295.76	450.35	482.52
Hainan	43.19	61.69	75.31	70.65	55.15	66.33	81.43	81.58	75.64	66.14	60.84	74.89
Chongqing	52.39	60.32	76.17	87.19	104.81	137.02	186.4	224.28	115.17	126.36	148.1	180.89
Sichuan	106.28	140.17	170.87	69.95	84.99	104.93	163.97	227.34	209.56	240.17	273.2	308.79
Guizhou	27.62	32.14	43.00	39.54	39.95	50.01	58.51	70.5	62.4	65.31	68.59	72.29
Yunnan	150.28	181	221.90	250.22	284.49	329.15	395.38	457.84	287.88	286.56	570.08	600.38
Shaanxi	92.84	106.1	123.13	125.73	145.08	212.17	270.41	335.24	253.47	266.3	293.03	338.2
Gansu	28.85	30.32	33.12	8.32	6.07	7.02	9.11	10.2	9.78	4.88	5.45	7.15
Qinghai	3.52	4.21	5.00	2.99	3.61	4.67	5.17	4.73	4.65	5.15	6.53	7.01
Ningxia	0.82	0.86	0.94	1.16	1.45	1.8	1.95	1.9	2.54	3.37	3.73	5.12
Xinjiang	33.11	36.2499	43.84	36.32	35.49	50.94	56.37	62.49	68.88	54.01	53.14	58.21

Consumption rate(%)(Unit: 10000 people) (Data from China Statistical Yearbook)												
Region	2005	2006	2007	2008	2009	2010	2011	2012	2013	2014	2015	2016
Beijing	51.4	53.4	54.3	57.5	55.6	56	58.4	59.6	61.3	62.5	63	60
Tianjin	40.8	40.4	40.9	34.1	38.2	38.3	37.9	37.8	39.2	39.8	43.3	44.8
Hebei	42.7	42.8	43.1	41.8	41.9	40.8	39.3	41.7	42	42.6	44.3	45.3
Shanxi	47.6	47.1	45.1	42.9	45.5	43.8	43.3	45.5	49.1	49.9	55.9	57.1
nner Mongoli	44	43.7	43.2	37.6	40.7	39.5	38.5	39.3	40.9	40.3	41.8	44.3
Liaoning	47.7	44.6	41.6	34.5	41.2	40.5	39.9	40.5	41.4	42.6	45.4	59.1
Jilin	51.7	43.1	46.2	45	44.3	41.1	39.6	38.9	39.4	37	36.1	37.7
Heilongjiang	48.3	47.8	49.7	51.3	55.7	53.1	52.4	53	55.4	58.1	59.6	62.3
Shanghai	48.3	49	49.4	50.5	51.3	54.9	56.4	57.1	57.9	58.8	59.1	57.4
Jiangsu	41.2	41.6	42	40.2	41.7	41.6	42	42	44.7	47.7	50	51
Zhejiang	47.4	47.2	46.1	43	46.4	45.7	46.5	47.6	47.2	48.2	48.8	48.2
Anhui	56.2	55.1	54.1	52.7	51.5	50.3	49.7	49	53.4	48.6	49.9	49.6
Fujian	50.2	48.6	45.7	44.2	42.8	42.6	40.7	40	38.6	38.7	39.8	40.3
Jiangxi	52.1	50.8	50.8	50.5	46.3	47.5	47.8	48.8	49.1	49.1	50.3	50.6
Shandong	43	43.1	43.3	43.4	40	39.1	39.9	41.1	41.3	40.7	41.5	47.3
Henan	50.6	49.7	45.5	42	44.9	44.2	43.8	45.1	47.5	48.2	50.6	51.3
Hubei	55.9	56.7	52.4	50.2	47.8	45.7	44.3	44.1	43.9	43.7	44.2	46.7
Hunan	62	60.9	58	53.6	50.9	47.4	46.2	45.9	46	46.1	51.1	51.1
Guangdong	51.6	49.2	48.8	49.4	47.1	46.7	49	51.3	51.8	50	51.1	50.6
Guangxi	60.8	58.1	55.1	52.1	55.9	50.7	47.8	50	51.5	52.2	52.8	53.7
Hainan	52.4	52	53.4	50.5	48.9	46.2	46.8	48.5	50.5	49.2	60.6	61.4
Chongqing	57.3	57.4	57.2	54.1	48.8	48.1	46.4	47.3	47.4	47.4	47.7	47.6
Sichuan	59	55.9	54	51	51	50.1	49.6	50	50.4	50.9	52.5	52.3
Guizhou	82.2	80	77.8	67.8	65.7	62.7	60.3	57.7	56.6	57.1	56.7	57.3
Yunnan	66.9	65.3	61.3	61.3	60.7	59.4	59.3	61.2	62.8	64	65	64.9
Shaanxi	45.8	41.4	45.2	41.1	47.7	45.3	44.5	44.2	44	44.2	45.5	45.3
Gansu	63	61	59.8	61.4	62.3	59.1	59.1	58.9	58.8	59	64.4	66
Qinghai	66.4	66	65	61.7	57	53	51.5	52.7	49.9	50.1	61.5	65.2
Ningxia	64.5	64	59.4	55	48.2	48.8	48.5	50.6	52.2	53.4	59.1	59.7
Xinjiang	48.6	52	54.8	54	53	52.7	53.2	56.8	55	54.2	60.5	63.8

Number of participants in basic endowment insurance for urban employees(Unit: 10000 people) (Data from China Labor Statistics Yearbook)												
Region	2005	2006	2007	2008	2009	2010	2011	2012	2013	2014	2015	2016
Beijing	519.9525	603.5652	671.0195	757.1514	826.6511	981.3317	1089.3879	1206.3791	1311.2997	1392.6052	1424.249	1546.64
Tianjin	308.2797	328.1957	344.757	376.5273	401.5257	431.4502	458.7054	490.263	520.6662	545.4325	565.179	639.031
Hebei	707.9442	747.5423	795.6097	862.528	919.5377	988.4371	1059.8147	1125.6194	1194.6676	1261.9536	1320.477	1403.137
Shanxi	383.4312	486.8727	506.6724	539.4184	563.7905	591.0313	623.7722	648.6951	672.4425	692.0289	714.27	760.21
nner Mongoli	338.9473	356.6343	370.8992	389.4734	410.8251	430.6858	452.3836	471.9461	496.4808	524.9431	578.956	655.017
Liaoning	1193.6043	1248.8297	1299.7233	1406.236	1457.398	1496.8954	1556.6129	1609.2422	1729.4706	1769.1827	1780.16	1800.298
Jilin	455.942	480.238	501.7147	525.2645	554.2552	599.4975	617.4668	632.1837	655.1923	676.6585	693.629	706.833
Heilongjiang	768.9254	800.9588	826.8398	857.7899	920.3373	952.2402	981.0213	1012.9915	1062.0641	1090.1002	1118.004	1144.142
Shanghai	830.0078	891.7338	932.4297	967.6948	1001.1037	1049.4664	1382.6571	1416.8959	1429.8823	1457.4346	1493.801	1527.138
Jiangsu	1345.5662	1469.8036	1602.2826	1751.6453	1883.0837	2033.0247	2223.945	2427.5407	2582.1118	2691.9098	2779.895	2861.534
Zhejiang	962.2665	1052.5827	1167.1033	1386.9056	1527.4317	1702.2175	1919.2199	2183.343	2375.3981	2548.0013	2504.281	2506.94
Anhui	471.7465	495.2226	530.32	578.414	628.1524	669.5378	729.2715	783.7594	811.3287	829.2494	857.513	892.237
Fujian	409.644	456.0671	512.8398	557.2266	585.8984	635.5057	695.0932	756.4937	812.8205	848.2816	883.666	979.765
Jiangxi	387.4304	414.9706	475.0301	550.3276	581.9294	607.5966	653.0302	707.3734	754.1837	783.8878	823.096	957.304

Shandong	1302.435	1368.0193	1457.0697	1565.8694	1661.0335	1773.0062	1907.0501	2063.1977	2259.5636	2370.1912	2477.458	2576.385
Henan	814.0044	863.7763	912.898	972.0333	1019.089	1079.3281	1168.3805	1270.631	1349.9892	1431.6355	1508.707	1848.423
Hubei	803.9603	850.7726	886.767	932.3252	982.0235	1039.7688	1113.4251	1171.3872	1219.3779	1266.2367	1315.512	1355.038
Hunan	718.6488	751.645	783.9859	829.0536	879.0727	938.9005	988.1913	1047.9779	1091.7322	1118.8914	1160.069	1186.654
Guangdong	1796.0947	1972.3244	2226.794	2444.2509	2716.4342	3215.1757	3800.7439	4034.0837	4183.0372	4809.4651	5086.527	5392.432
Guangxi	288.6039	302.7058	325.4525	368.0521	411.3232	449.2947	483.7549	512.6503	538.3687	557.5905	576.629	751.912
Hainan	120.9186	132.0468	141.737	156.1943	168.0826	180.8071	199.855	214.1629	231.4981	242.3242	249.848	224.93
Chongqing	290.1555	317.3488	344.773	406.1038	492.8359	584.3624	647.5558	716.8569	773.124	825.5042	849.292	952.245
Sichuan	793.4084	842.6511	917.3852	1017.8567	1176.1843	1300.8617	1494.2446	1615.3527	1720.2586	1839.6905	1938.984	2157.603
Guizhou	183.6792	193.2484	205.928	215.8926	235.6135	257.3053	282.0634	309.3767	337.2946	361.4535	392.095	423.577
Yunnan	258.6897	267.4192	279.3615	293.7182	306.5407	317.4171	342.8186	364.4729	384.3192	397.892	412.944	581.796
Shaanxi	376.1252	391.5216	408.0842	433.3678	458.8383	550.3573	588.6216	643.4911	684.9841	716.4585	751.707	790.824
Gansu	197.304	201.1748	208.4464	220.9942	230.9138	242.4796	262.9541	277.3694	288.3981	298.8492	306.209	314.967
Qinghai	59.9803	62.4638	65.1729	68.3026	71.3455	74.4292	81.515	86.0137	90.3053	94.5655	100.074	132.31
Ningxia	67.5491	72.338	77.0185	82.5557	89.3812	107.7866	121.4124	131.2306	143.7698	151.4192	157.516	189.274
Xinjiang	302.1	313.3	327.7246	346.3181	356.9149	393.7858	431.5141	458.7604	476.3368	490.7549	499.446	624.983

Actual sales area of residential housing(Unit: 10000 square meters) (Data from China Statistical Yearbook, China Real Estate Statistical Yearbook)												
Region	2005	2006	2007	2008	2009	2010	2011	2012	2013	2014	2015	2016
Beijing	2823.6529	2205.0296	1731.4798	1031.43	1880.45	1201.39	1034.96	1483.37	1363.67	1136.53	1126.84	981.37
Tianjin	1264.3849	1332.4916	1401.8481	1135.35	1461.47	1302.61	1365.71	1511.4	1720.34	1483.64	1674.78	2521.87
Hebei	1322.3216	1692.4231	1969.1234	2128.86	2819.77	4325.12	5293.18	4622.46	5020.13	5015.06	5161.65	5899.72
Shanxi	619.8563	740.7279	846.503	893.1	964.05	1070.54	1170.88	1390.44	1484.37	1433.91	1481.14	1881.51
nner Mongoli	918.4265	1250.0996	1809.7306	2093.34	2253.98	2569.84	2911.16	2104.22	2263.65	1995.68	1944.92	2073.36
Liaoning	2340.3556	2730.7384	3545.5509	3731.19	4864.25	6013.5	6624.05	7655.4	8014.8	4932.08	3477.26	3383.08
Jilin	745.9214	879.2384	1184.3969	1435.73	1758.37	2105.33	2122.39	2159.43	1985.95	1387.87	1304.82	1630.72
Heilongjiang	1048.2603	1298.5068	1518.5671	1286.62	1751.22	2385.68	2947.84	3226.22	2944.23	2131.46	1710.6	1797.02
Shanghai	2845.6969	2615.4915	3279.1745	2007.48	2928.04	1690.82	1500	1592.63	2015.81	1780.91	2009.17	2019.8
Jiangsu	4523.1432	5317.2013	6772.7134	5282.89	9034.69	8112.37	6767.25	7923.37	10191.52	8800.93	10275.95	12657.66
Zhejiang	2824.6482	3039.5413	3924.9182	2480.74	4760.12	3833.74	2757.18	3316.23	4097.63	3941.49	5131.88	7234.19
Anhui	1686.0304	2037.5447	2775.6617	2542.6	3646.44	3641.88	3991.99	4275.43	5573.53	5364.94	5356.81	7506.87
Fujian	1720.555	1743.3904	2096.3689	1250	2420.83	2139.26	2213.3	2741.96	3957.46	3324.1	3315.69	4134.46
Jiangxi	1454.512	1635.2608	2037.8732	1604.86	2108.07	2265.72	2156.77	2125.9	2846.04	2775.22	3145.83	4140.55
Shandong	3417.7512	3824.4151	4710.6645	5039.4	6478.28	8448.26	8741.16	7745.87	9300.29	7972.49	8526.85	10598.58
Henan	1539.6044	2190.9901	3569.1829	2943.36	4017.45	5092.49	5725.12	5455.5	6561.41	7009.09	7645.84	10137.13
Hubei	1549.1866	1915.2966	2381.9082	1821.31	2576.32	3236.88	3788.68	3620.1	4765.68	5002.6	5647.72	6789.21
Hunan	1611.596	1815.0118	2498.8296	2413.7	3262.34	4140.07	4455.59	4664.08	5411.48	4852.32	5671.19	7190.66
Guangdong	4546.315	4693.3884	5603.8771	4360.45	6567.43	6552.81	6706.6	7157.63	8830.95	8163.56	10497.62	13021.97
Guangxi	1314.3702	1372.3957	1855.5035	1637.99	2249.7	2607.15	2749.33	2546.96	2765.15	2869.32	3181.51	3864.01
Hainan	233.0764	193.7266	299.5932	358.72	545.91	834.19	819.02	898.35	1154.86	942.84	984.79	1417.09
Chongqing	1792.414	2011.703	3310.1292	2669.93	3771.22	3986.31	4063.42	4105.11	4359.19	4423.68	4477.71	5105.46
Sichuan	3028.5455	3738.8884	4562.4651	3247.32	5553.13	5849.34	5826.52	5679.33	6505.32	6176.51	6495.43	7884.09
Guizhou	774.7121	809.5586	989.3858	848.11	1541.91	1596.3	1698.52	2002.4	2646.98	2707.09	2943.37	3426.96
Yunnan	1294.9535	1500.1867	1792.8355	1478.25	2040.33	2658.99	2829.58	2789.68	2855.52	2618.02	2576.8	2933.1
Shaanxi	849.4304	1060.0763	1377.9062	1426.06	1995.67	2471.95	2874.51	2530.84	2831.22	2836.69	2717.98	3012.61
Gansu	439.8286	482.8996	556.2469	588.63	659.38	692.07	758.15	893.36	1134.81	1212.6	1307.48	1478.81
Qinghai	108.4571	110.9646	147.3356	141.23	208.02	266.43	342.56	246.85	369.7	362.92	329.71	373.03
Ningxia	319.1585	337.0199	448.2252	453.26	677.98	816.79	705.13	707.57	928.26	939.37	708.12	830.22
Xinjiang	606.4836	806.4638	1078.2814	886.35	1326.67	1450.02	1570.56	1274.8	1799.95	1541.25	1536.49	1543.66

Administrative area of each region(Unit: 10000 square kilometers) (Data from China Statistical Yearbook)												
Region	2005	2006	2007	2008	2009	2010	2011	2012	2013	2014	2015	2016
Beijing	1.641	1.641	1.641	1.641	1.641	1.641	1.641	1.641	1.641	1.641	1.641	1.641
Tianjin	1.1946	1.1946	1.1946	1.1946	1.1946	1.1946	1.1946	1.1946	1.1946	1.1946	1.1946	1.1946
Hebei	18.88	18.88	18.88	18.88	18.88	18.88	18.88	18.88	18.88	18.88	18.88	18.88
Shanxi	15.67	15.67	15.67	15.67	15.67	15.67	15.67	15.67	15.67	15.67	15.67	15.67
nner Mongoli	118.3	118.3	118.3	118.3	118.3	118.3	118.3	118.3	118.3	118.3	118.3	118.3
Liaoning	14.8	14.8	14.8	14.8	14.8	14.8	14.8	14.8	14.8	14.8	14.8	14.8
Jilin	18.74	18.74	18.74	18.74	18.74	18.74	18.74	18.74	18.74	18.74	18.74	18.74
Heilongjiang	47.3	47.3	47.3	47.3	47.3	47.3	47.3	47.3	47.3	47.3	47.3	47.3
Shanghai	0.634	0.634	0.634	0.634	0.634	0.634	0.634	0.634	0.634	0.634	0.634	0.634
Jiangsu	10.32	10.32	10.32	10.32	10.32	10.32	10.32	10.32	10.32	10.32	10.32	10.32
Zhejiang	10.43	10.43	10.43	10.43	10.43	10.43	10.43	10.43	10.43	10.43	10.43	10.43
Anhui	14.01	14.01	14.01	14.01	14.01	14.01	14.01	14.01	14.01	14.01	14.01	14.01
Fujian	12.4	12.4	12.4	12.4	12.4	12.4	12.4	12.4	12.4	12.4	12.4	12.4
Jiangxi	16.69	16.69	16.69	16.69	16.69	16.69	16.69	16.69	16.69	16.69	16.69	16.69
Shandong	15.58	15.58	15.58	15.58	15.58	15.58	15.58	15.58	15.58	15.58	15.58	15.58
Henan	16.7	16.7	16.7	16.7	16.7	16.7	16.7	16.7	16.7	16.7	16.7	16.7
Hubei	18.59	18.59	18.59	18.59	18.59	18.59	18.59	18.59	18.59	18.59	18.59	18.59
Hunan	21.18	21.18	21.18	21.18	21.18	21.18	21.18	21.18	21.18	21.18	21.18	21.18
Guangdong	17.97	17.97	17.97	17.97	17.97	17.97	17.97	17.97	17.97	17.97	17.97	17.97
Guangxi	23.67	23.67	23.67	23.67	23.67	23.67	23.67	23.67	23.67	23.67	23.67	23.67
Hainan	3.54	3.54	3.54	3.54	3.54	3.54	3.54	3.54	3.54	3.54	3.54	3.54
Chongqing	8.24	8.24	8.24	8.24	8.24	8.24	8.24	8.24	8.24	8.24	8.24	8.24
Sichuan	48.6	48.6	48.6	48.6	48.6	48.6	48.6	48.6	48.6	48.6	48.6	48.6
Guizhou	17.6167	17.6167	17.6167	17.6167	17.6167	17.6167	17.6167	17.6167	17.6167	17.6167	17.6167	17.6167
Yunnan	39.41	39.41	39.41	39.41	39.41	39.41	39.41	39.41	39.41	39.41	39.41	39.41
Shaanxi	20.58	20.58	20.58	20.58	20.58	20.58	20.58	20.58	20.58	20.58	20.58	20.58
Gansu	42.59	42.59	42.59	42.59	42.59	42.59	42.59	42.59	42.59	42.59	42.59	42.59
Qinghai	72.1	72.1	72.1	72.1	72.1	72.1	72.1	72.1	72.1	72.1	72.1	72.1
Ningxia	6.64	6.64	6.64	6.64	6.64	6.64	6.64	6.64	6.64	6.64	6.64	6.64

Xinjiang	166	166	166	166	166	166	166	166	166	166	166	166

Total assets of state-owned construction enterprises(Unit: 10000 yuan) (Data from China Statistical Yearbook, China Construction Industry Statistical Yearbook)												
Region	2005	2006	2007	2008	2009	2010	2011	2012	2013	2014	2015	2016
Beijing	17188832.9	16860402.1	23085134	13546435	14902217	14932842	16403442	18063256	22320490	36082621	38548710	40447255
Tianjin	2855637.2	2451628.9	3487100.4	5143340.5	5531822.9	5940228.7	7211125	10074649	6463419	5934373	7209857	7424233
Hebei	3282951.7	3248197	4260102.4	3533867.4	4229297.7	4794437.2	5785910	6562179	6126689	6085587	5947892	6635210
Shanxi	4357141.1	4090435.6	3744472.8	3753303.6	3736032.9	4413282.2	6095906	7562400	7622216	8204668	8093201	10201650
nner Mongoli	226465.8	280987.4	594134.2	754718.8	797583.4	920122.5	1041083	1277917	1249080	1654136	2507390	2964442
Liaoning	3557037.6	3745868.7	3902014.8	4741134.5	5603269.8	5268885.5	7643550	9287976	8064444	9222947	8332340	8263564
Jilin	1793840.5	918854.9	849292.3	866852.6	874918.8	815458.1	1199811	1337783	1241827	1222824	2560577	1483063
Heilongjiang	1889407.5	2418050.2	3011719.3	3964547.1	3821000.3	3675323.4	5106373	5174080	4672794	4319697	4295068	4448894
Shanghai	4650553.2	4920426.2	4677530.6	7206144.4	7548658.8	8740665.6	10098891	11695611	11559302	10207810	11047873	12444691
Jiangsu	3806893.1	4019661.5	4488892.7	4651614.2	5742240.5	6707002.3	8147964	10298723	8386739	10133069	10800049	11718212
Zhejiang	1723520.7	1831647.7	2188929.9	2167346.4	1948908.6	2121798.4	3222567	3736669	3463031	3727521	3585155	3372703
Anhui	2318512.9	3184970	4002078.3	4933243.5	6352585	7401744.3	5877310	6578144	7073291	8267126	8865822	10998886
Fujian	2019765.5	2030534.7	2147978.3	2677518.2	3398199.6	3235483.4	3434840	4053320	3193402	3633771	4616947	5452277
Jiangxi	1858393	1809980.7	2147431.3	2773478.4	3198002.9	3392904.2	3618983	3950484	3155159	3120554	3557886	3993995
Shandong	5024441	5295654.1	6498546	6646134.2	7559127.5	9769779.1	12029664	13150404	12792841	14895307	17136616	20101714
Henan	3184295.1	3213646.7	3790922.7	4431985.6	5237177.9	5178471.5	6002118	7125470	6472547	6988816	7322855	8691272
Hubei	4458031.5	4473505.9	4366991.9	8344256.3	15185045	13087088	16899622	19505913	19105177	22402909	31096261	32173634
Hunan	2977561.5	3596690	4353054.9	4849044.7	6200331	7174919.5	8588087	7499391	6800569	8078650	9693889	11984621
Guangdong	8439749.6	7951559.7	9385394.8	10133548	10415107	13914361	16100775	18352377	19438993	20686454	18887141	21408212
Guangxi	2332490	2186815.7	2327741.5	2810950.7	3145924.3	3663064.8	4727907	5245533	6203888	6587643	7099303	7271895
Hainan	178243.7	182651.6	171538.4	338382.6	401175.6	422350.1	640658	712397	373293	356036	379599	434409
Chongqing	1263180.2	1616035.6	1907753.2	2837195.7	3599459.2	3780614.5	5783578	7311204	6507551	6685149	7072853	8375060
Sichuan	5001410.3	4944742.7	4920527.5	7201910.3	7541188.5	8578198.4	12473560	14522742	14139808	15564664	17591954	16851370
Guizhou	1881925.5	2170096.7	2263351	2721004.8	2999402.8	3969113.6	5163132	7124294	6794333	8428095	12012839	17923159
Yunnan	1366011.8	1542089.5	2022177.2	2405739.8	2530232.9	3626756.7	4470584	5434235	5341976	7058386	9103582	14967492
Shaanxi	2883618.5	3312853.8	4810754.4	7055126.1	5771876.7	7784930	6746799	8886741	6720566	7715505	9130655	10332847
Gansu	1211640.7	1264275.5	1261631.1	990656.5	1017233.4	1169996.3	1802228	3793203	2158455	2396701	2759932	3447768
Qinghai	543350.3	570959.3	658725.9	924294.7	989412.3	1091785.2	1474320	1990584	2075874	2451999	2590098	2872342
Ningxia	458752	494979.9	528756.7	553796.5	659652.2	757387.9	859043	1069909	1149575	1453284	1471729	1366358
Xinjiang	1178845	1336968	1509053.1	1639995.8	1945763.3	1789436.3	2570904	2760552	2715534	2655152	3045125	3782748

otal assets of construction enterprises above designated size(Unit: 100 million yuan) (Data from China Statistical Yearbook, China Construction Industry Statistical Yearboo												
Region	2005	2006	2007	2008	2009	2010	2011	2012	2013	2014	2015	2016
Beijing	32714956.4	35736877.5	46620233	55908651	69965367	88979807	108092880	117600352	136831634	162812465	183659721	202636726
Tianjin	7384760.4	9176834.6	11997954	16011201	19886825	23974077	30791742	39248602	44865074	52720198	57219100	60167213
Hebei	9764736.2	10574714.7	12500542	16014722	18849275	22001384	26196440	32436006	37921097	41561072	44823884	49726839
Shanxi	8504544	9497871.7	10859790	13549162	16358752	20268277	25034786	29761637	34202261	37939935	40337922	48453897
nner Mongoli	3441532.1	3874639.2	4941411.1	5902372.8	7425047.9	9735992.3	13217648	16175581	17886901	17963082	18725376	19758626
Liaoning	12539488.2	14568798.7	17011132	19399640	24072001	31813950	39897618	51045894	55032788	63315459	68779289	59845041
Jilin	4857475	5230565.6	5197781.3	6535074.7	6339398.2	8731874.8	11076683	16779574	19293029	20870699	23449197	24185056
Heilongjiang	7038065.8	8203440.1	9142571.2	10397027	10829222	11951907	14595943	16398329	18033052	17955882	17274374	19576338
Shanghai	20777866.1	25173090.9	29094656	36920878	41538292	48369587	56538718	65533626	72752012	79425938	86446890	90496425
Jiangsu	32095807.4	39070068.4	45236905	53947024	62669839	73874618	97748467	115561174	134237735	150780767	164330243	178352447
Zhejiang	25943721.1	30669657.7	36754947	41641090	48901065	61879270	75503558	86603741	98324153	109100038	116542560	120878776
Anhui	7716162.2	9195762.1	11726821	13957253	17024096	21150759	25975806	31696142	36541088	42665144	47335318	54963345
Fujian	7725253.1	8966977.8	10442704	12458511	14609997	17347043	20820441	25480227	31273033	38115217	42664757	47584471
Jiangxi	4486359.9	4991030.8	5566435.7	6954046.4	8114543	9450774	11252405	15469748	19312471	23606780	26984565	34474766
Shandong	23373281	26516230	30565566	35056790	40713260	48111134	59757178	69483152	81774689	91202531	99206358	111358708
Henan	9230042	11235087.5	14768953	18939907	23830698	28531774	35588319	41440134	49472704	57630749	61702253	70435847
Hubei	11400956.8	13434936.9	19680916	22549260	31126995	37563487	48743237	58525342	63518819	75575328	88949139	98533062
Hunan	8213667	9401553.6	11053201	12831670	14624101	17255836	20985943	25179114	29805043	34387744	39953782	46319249
Guangdong	25926241.1	28244404.1	32825696	37467354	43389638	51012221	61366181	72945325	88215591	98843618	107374057	122000895
Guangxi	4555244.6	4582672	5203292.8	6139335.9	6890105.7	7765458.1	9778957	11241396	13839071	15694735	17094285	18981517
Hainan	598573.8	618904.7	406822.9	833385.5	947501.9	840327.7	1477832	1686772	2064568	2144442	2152272	2515039
Chongqing	6668558.2	8058935.2	9548123.9	12026040	15171178	19592948	26426441	31933147	40256771	45663501	49040475	53259373
Sichuan	14464214.3	14924153.4	16408116	20668583	24505665	33818810	47115894	52404093	60963372	69889625	77654931	98587197
Guizhou	2858639	3178261.4	3469707	3971954.5	4632227.9	5937136.7	7313506	10201530	14518652	18016556	26087102	35439953
Yunnan	6360031.9	7252161.4	8254202.7	10032425	11264239	14498808	17378059	20966424	25924958	30583845	36943637	45905599
Shaanxi	5830474.1	6618675.8	8691412.9	12545428	21420271	18364597	21035153	27486331	33837635	40123874	46703921	53441687
Gansu	3409363.5	3477593.5	4003927.4	4299312	4800632.6	5512297.9	6684843	11319040	13619280	14892340	16903621	18634425
Qinghai	1123283.4	1195939.4	1431453.4	1757265.3	1961633	2217839.4	2898100	3631771	4170533	4825717	5098509	5680858
Ningxia	1627824.4	1796836.8	1889855	2157776.1	2514166	2961617.3	3661264	4408589	5278587	6363355	6895018	7476294
Xinjiang	3784693.2	4082342	4543478.1	5485244.5	6494981.3	7797928.1	10513737	12995836	16196519	17847771	20383343	23193403

Investment in comprehensive agricultural development projects(Unit: 10000 yuan) (Data from China Financial Yearbook)												
Region	2005	2006	2007	2008	2009	2010	2011	2012	2013	2014	2015	2016
Beijing	24620	25396	28107	63288	67618.145	71948.29	119850.58	86670.57	70022	74008	58575	70025.06
Tianjin	22673.2	20933.9	23368.08	24530.7	28106.45	31682.2	74962	68030	59805	76535	76306	63403
Hebei	66058.07	72400.79	90124.44	82722.61	106518.93	130315.24	168996.13	219938.98	253521.11	275500.39	284723.29	284123.45
Shanxi	38859.1	44764.8	49658.48	49085.1	57450.75	65816.4	81201.7	94840.89	108161.3	118014.82	124176.4	125941.58
nner Mongoli	62381.6	74343.3	75302.9	96414.4	120434.3	144454.2	173321.8	235050.8	261272.18	292398.28	323499.79	324982.77
Liaoning	99476.9	102841.5	117099.44	115496	124018.5	132541	148821.75	183867.5	201879.5	219206	244389	246402.6
Jilin	80213.44	76255.1	72956.2	91348.2	103924.1	116500	145162	200652.73	215655.4	232829.1	264681.28	261132.6
Heilongjiang	89358	93086.82	100429.65	114705.44	151339.58	187973.71	171888	247640	281571	270127.5	317157.6	265279.56
Shanghai	19045.79	21077.01	23439.99	21010.38	24925.68	28840.98	24496	24392	24837.95	45702.26	40478.77	44015.05
Jiangsu	72422.5	89620.76	102861.72	116617.8	139551.55	162485.3	198869.79	255348.24	279815.31	317131.19	336365.6	343636.19

Zhejiang	91338.13	107575.03	104160.35	111290.77	111532.54	111774.3	118076.1	134884.72	144909.2	175973.77	189454.06	186251.04
Anhui	76464.18	70211.28	92654.91	96014.69	115155.43	134296.17	148062.58	187731.61	204418.7	229851.31	259922.21	263315.48
Fujian	47256.97	52676.32	65892.04	81460.24	82379.735	83299.23	73620.51	90687.65	101160.32	117941.5	123117.61	123938.3
Jiangxi	56263.39	63302.53	70516.56	72443.05	93459	114474.95	118827.84	146835.28	163237	181393.82	158995.51	256870.78
Shandong	85807.7	106173.98	118346.15	127276.85	137875.17	148473.49	162135.37	205126.95	216060.51	246134	268484.68	280742.11
Henan	65689.41	86243.86	97914.06	93388.37	119566.94	145745.51	171042.75	234084	278292.08	303379.91	333407.41	314744.11
Hubei	60162.65	78102.27	82966.42	84815.75	103238.64	121661.53	135822	169760.6	191661.78	224981.27	237763.37	253437.71
Hunan	68063	79326.92	84584.41	90444.55	107391.08	124337.61	139478.9	185941	212836.75	234596	257672.89	272965.47
Guangdong	40714.73	50391.16	47971	47611.5	57975.42	68339.34	81087.1	100457.6	106323.63	123540	126033.45	124754.37
Guangxi	40981.51	40733.99	42036.6	50477.5	61704.9	72932.3	80505.15	94060.1	110945.3	122693.85	132857.25	141950.63
Hainan	25524	27424.5	23607.6	34352.5	42075.64	49798.78	54287.6	61886.87	69818.34	70997.31	73432.03	66665.76
Chongqing	37689.03	44063.72	43944.88	49841.18	59696.835	69552.49	78221.2	89442.25	102870.35	117981.26	128269.87	251117.4
Sichuan	87039.52	88380.34	92510.66	104229.86	119288.91	134347.96	146151.04	176361.5	195652.93	216335.82	242696.93	132520.55
Guizhou	30302.37	30373.98	32498.46	36724.06	45371.38	54018.7	116106.32	69642.72	98891.55	108516.6	112997.03	173051.02
Yunnan	53050.86	55070.65	61853.22	69479.51	76935.66	84391.81	96105.12	111639.65	129900.05	139656.3	168458.87	118258.56
Shaanxi	40350	48409.98	56976.16	79086.04	80959.77	82833.5	87162.05	98421.2	115585.8	124331.7	136970.5	143929.5
Gansu	30000.13	36921.19	43837.4	46307.38	56150.99	65994.6	75533.5	84863.5	92825.5	104524.7	120860.8	121899.04
Qinghai	20756.9	20426.73	24820.16	27494.96	33880.08	40265.2	44103.64	56003	64284.74	69848.01	73783.03	77371.79
Ningxia	16915.88	24331.5	24131.28	27961.5	39098.475	50235.45	58721.76	61981.33	69488.98	77837.92	93504.61	91096.74
Xinjiang	39447.37	46961.1	51951.55	54255.78	63004.115	71752.45	84657.48	100058.08	116225.24	111694.02	146686.42	147358.7

nvestment in fixed assets of the whole societye(Unit: 100 million yuan) (Data from provincial and municipal statistical yearbooks, the new China 60 years of statistical comp												
Region	2005	2006	2007	2008	2009	2010	2011	2012	2013	2014	2015	2016
Beijing	2827.2	3371.5	3966.6	3848.5	4858.4	5493.5	5910.6	6462.8	7032.2	7562.3	7990.9	8461.7
Tianjin	1516.84	1849.8	2388.63	3404.1	5006.32	6511.42	7510.67	8871.31	10121.21	11654.09	13065.18	14629.22
Hebei	4210.25	5501	6884.68	8866.56	12311.85	15083.35	16389.33	19661.28	23194.23	26671.92	29448.27	31750.02
Shanxi	1859.3969	2321.4735	2927.1653	3635.1396	5033.5333	6352.6011	7373.0582	9176.3142	11200.238	12354.53	14137.159	14284.982
nner Mongoli	1808.31	2291.7	2963.4	3770.67	5069.29	6035.68	7332.86	8821.13	10441.6	12074.24	13824.76	15469.5
Liaoning	4234.1	5689.6	7435.2	10019.1	12292.6	16043	17726.3	21836.3	25107.7	24730.8	17917.9	6692.2
Jilin	1802.4	2804.3	4003.2	5608.2	7259.5	9621.8	7441.7	9511.6	9979.3	11339.6	12705.3	13923.2
Heilongjiang	1731.9	2235.9	2864.2	3656	5028.8	6801.7	7475.4	9780.2	11453.1	9828.9	10182.9	10648.4
Shanghai	3542.55	3925.09	4458.61	4829.45	5273.33	5317.67	5067.09	5254.38	5647.79	6016.43	6352.7	6755.88
Jiangsu	8739.71	10071.42	12268.07	15060.45	18949.88	23184.28	26314.66	31706.58	35982.52	41552.75	45905.17	49370.85
Zhejiang	6696.25	7593.66	8420.43	9323	10742.32	12376.04	14077.25	17095.96	20194.07	23554.76	26664.72	29571
Anhui	2521	3544.7	5093.7	6799.9535	9263.1822	11849.434	12147.779	15054.951	18251.121	21256.294	23965.552	26758.112
Fujian	2344.733	3115.0775	4321.7404	5301.6939	6362.0327	8273.4186	10119.468	12709.66	15526.869	18449.479	21628.306	23107.49
Jiangxi	2168.9712	2683.5744	3301.9427	4345.4333	5693.1422	7164.625	8737.5985	10774.158	12850.253	15079.255	17388.128	19694.21
Shandong	10541.87	11136.06	12537.02	15435.93	19030.97	23276.69	26769.73	31255.96	36789.07	42495.55	48312.46	53322.49
Henan	4378.69	5907.74	8010.11	10490.65	13704.65	14124.69	17770.51	21449.99	26087.45	30782.17	35660.34	40415.09
Hubei	2834.75	3572.69	4534.14	5798.56	8211.85	10802.69	12935.02	16504.17	20753.91	25001.77	29191.06	29503.88
Hunan	2563.96	3242.39	4294.36	5649.69	7695.27	9821.06	11431.48	14576.61	18381.44	21950.77	25954.27	27688.45
Guangdong	7164.11	8132.37	9596.95	11165.06	13353.15	16113.19	16843.83	19307.53	22828.65	25928.09	30031.2	33008.86
Guangxi	1769.0715	2246.5743	2970.0845	3783.1385	5706.6957	7859.066	10160.453	12635.218	11907.667	13843.212	16227.782	18236.779
Hainan	379.4284	426.0137	509.2568	709.0144	1002.3333	1331.4557	1611.4087	2145.3762	2725.3954	3039.4554	3355.4048	3747.0251
Chongqing	2006.318	2451.8351	3161.5147	4045.2509	5317.9185	6934.7966	7685.8699	9380.0012	11205.028	13223.746	15480.325	17361.121
Sichuan	3477.6772	4521.74	5855.3	7602.4	12017.28	13581.96	15124.09	18038.92	21049.15	23577.17	25973.74	29126.03
Guizhou	1018.25	1197.68	1488.8	1864.45	2450.99	3186.28	5101.55	5717.8049	7373.6013	9025.7503	10945.543	13203.996
Yunnan	1755.3	2220.45	2798.89	3526.6	4527.02	5528.71	6185.3	7831.1	9968.3	11498.58	13500.62	16119.4
Shaanxi	1982.04	2610.22	3642.13	4851.41	6553.39	8561.24	10023.53	12840.15	15934.21	18709.49	20177.86	20825.25
Gansu	874.525	1024.8651	1310.3759	1735.7895	2479.5998	3378.1	4180.24	5040.53	6407.2	7759.62	8626.6	9534.1023
Qinghai	367.15	419.62	487.47	582.85	800.51	1068.73	1434.33	1920.03	2403.9	2908.71	3266.64	3533.1918
Ningxia	444.8174	515.2753	621.8092	858.8368	1119.1392	1464.6972	1654.1517	2109.5182	2681.1429	3200.9849	3532.9268	3835.4633
Xinjiang	1354.3432	1577.3966	1907.0184	2272.5814	3034.5365	3784.4438	5126.5602	6672.5427	8268.1763	9793.963	10656.525	9388.8871

Total GDP of China(Unit: 100 million yuan) (Data from China Financial Yearbook)												
Region	2005	2006	2007	2008	2009	2010	2011	2012	2013	2014	2015	2016
Beijing	187318.9	219438.5	270232.3	319515.5	349081.4	413030.3	489300.6	540367.4	595244.4	643974	689052.1	744127.2
Tianjin	187318.9	219438.5	270232.3	319515.5	349081.4	413030.3	489300.6	540367.4	595244.4	643974	689052.1	744127.2
Hebei	187318.9	219438.5	270232.3	319515.5	349081.4	413030.3	489300.6	540367.4	595244.4	643974	689052.1	744127.2
Shanxi	187318.9	219438.5	270232.3	319515.5	349081.4	413030.3	489300.6	540367.4	595244.4	643974	689052.1	744127.2
nner Mongoli	187318.9	219438.5	270232.3	319515.5	349081.4	413030.3	489300.6	540367.4	595244.4	643974	689052.1	744127.2
Liaoning	187318.9	219438.5	270232.3	319515.5	349081.4	413030.3	489300.6	540367.4	595244.4	643974	689052.1	744127.2
Jilin	187318.9	219438.5	270232.3	319515.5	349081.4	413030.3	489300.6	540367.4	595244.4	643974	689052.1	744127.2
Heilongjiang	187318.9	219438.5	270232.3	319515.5	349081.4	413030.3	489300.6	540367.4	595244.4	643974	689052.1	744127.2
Shanghai	187318.9	219438.5	270232.3	319515.5	349081.4	413030.3	489300.6	540367.4	595244.4	643974	689052.1	744127.2
Jiangsu	187318.9	219438.5	270232.3	319515.5	349081.4	413030.3	489300.6	540367.4	595244.4	643974	689052.1	744127.2
Zhejiang	187318.9	219438.5	270232.3	319515.5	349081.4	413030.3	489300.6	540367.4	595244.4	643974	689052.1	744127.2
Anhui	187318.9	219438.5	270232.3	319515.5	349081.4	413030.3	489300.6	540367.4	595244.4	643974	689052.1	744127.2
Fujian	187318.9	219438.5	270232.3	319515.5	349081.4	413030.3	489300.6	540367.4	595244.4	643974	689052.1	744127.2
Jiangxi	187318.9	219438.5	270232.3	319515.5	349081.4	413030.3	489300.6	540367.4	595244.4	643974	689052.1	744127.2
Shandong	187318.9	219438.5	270232.3	319515.5	349081.4	413030.3	489300.6	540367.4	595244.4	643974	689052.1	744127.2
Henan	187318.9	219438.5	270232.3	319515.5	349081.4	413030.3	489300.6	540367.4	595244.4	643974	689052.1	744127.2
Hubei	187318.9	219438.5	270232.3	319515.5	349081.4	413030.3	489300.6	540367.4	595244.4	643974	689052.1	744127.2
Hunan	187318.9	219438.5	270232.3	319515.5	349081.4	413030.3	489300.6	540367.4	595244.4	643974	689052.1	744127.2
Guangdong	187318.9	219438.5	270232.3	319515.5	349081.4	413030.3	489300.6	540367.4	595244.4	643974	689052.1	744127.2
Guangxi	187318.9	219438.5	270232.3	319515.5	349081.4	413030.3	489300.6	540367.4	595244.4	643974	689052.1	744127.2
Hainan	187318.9	219438.5	270232.3	319515.5	349081.4	413030.3	489300.6	540367.4	595244.4	643974	689052.1	744127.2
Chongqing	187318.9	219438.5	270232.3	319515.5	349081.4	413030.3	489300.6	540367.4	595244.4	643974	689052.1	744127.2
Sichuan	187318.9	219438.5	270232.3	319515.5	349081.4	413030.3	489300.6	540367.4	595244.4	643974	689052.1	744127.2
Guizhou	187318.9	219438.5	270232.3	319515.5	349081.4	413030.3	489300.6	540367.4	595244.4	643974	689052.1	744127.2
Yunnan	187318.9	219438.5	270232.3	319515.5	349081.4	413030.3	489300.6	540367.4	595244.4	643974	689052.1	744127.2

Shaanxi	187318.9	219438.5	270232.3	319515.5	349081.4	413030.3	489300.6	540367.4	595244.4	643974	689052.1	744127.2
Gansu	187318.9	219438.5	270232.3	319515.5	349081.4	413030.3	489300.6	540367.4	595244.4	643974	689052.1	744127.2
Qinghai	187318.9	219438.5	270232.3	319515.5	349081.4	413030.3	489300.6	540367.4	595244.4	643974	689052.1	744127.2
Ningxia	187318.9	219438.5	270232.3	319515.5	349081.4	413030.3	489300.6	540367.4	595244.4	643974	689052.1	744127.2
Xinjiang	187318.9	219438.5	270232.3	319515.5	349081.4	413030.3	489300.6	540367.4	595244.4	643974	689052.1	744127.2

Number of beds in health institutions(Unit: 10000 sheets) (Data from China Financial Yearbook)												
Region	2005	2006	2007	2008	2009	2010	2011	2012	2013	2014	2015	2016
Beijing	79067	81440	83925	86153	90100	92764	94735	100167	104011	109811	111555	117041
Tianjin	41513	43600	44335	46054	46353	48828	49423	53509	57743	60869	63693	65832
Hebei	162061	173024	195637	213965	232638	249725	266479	284359	303497	322909	342096	360485
Shanxi	107980	112105	108742	127263	144517	155885	157132	165309	172620	177442	183209	189689
nner Mongoli	69047	69753	73900	81068	87390	93350	100633	110788	120065	129011	133889	139236
Liaoning	177524	179415	179952	182972	191492	204208	215815	230962	241860	255513	266986	284384
Jilin	87279	90492	94373	99329	108345	115057	121240	127756	133245	140995	144500	151195
Heilongjiang	119692	123176	126058	135600	146572	159914	165255	178210	189183	201337	212590	220054
Shanghai	89758	93214	95960	97352	99704	105083	107130	109784	114314	117510	122813	129166
Jiangsu	197515	208902	220369	236541	250809	269548	296390	333118	368287	392293	413612	443060
Zhejiang	140734	148122	154663	160873	170199	184097	194759	213286	230056	245756	272509	289870
Anhui	126587	133321	139625	159724	174483	188010	204210	222315	235959	252044	267405	281720
Fujian	81645	84536	79645	88579	104290	113043	124232	139341	156149	164781	173007	174767
Jiangxi	84836	88061	94862	105106	115445	124640	135570	163721	174299	186727	197837	209097
Shandong	247574	258425	278791	319905	347052	382254	416148	473768	489737	500631	519369	540994
Henan	212407	223810	239511	268004	302378	327569	349612	393993	429810	459338	489621	521546
Hubei	139147	142152	150633	167673	187156	200394	223980	252991	288169	317500	343147	360558
Hunan	151297	159377	172394	187732	212043	233510	257687	287013	314090	355485	396950	425757
Guangdong	208936	220315	234179	250497	271982	300083	325038	355274	378367	405751	435666	465142
Guangxi	93532	96765	105223	118365	131569	143695	152039	168691	187216	201600	214485	224471
Hainan	18651	19927	20767	21889	23526	25981	28465	30289	32100	34466	38698	40324
Chongqing	64174	68250	74635	81950	92709	103624	115627	130813	147436	160579	176549	190850
Sichuan	193171	200344	214512	243746	275085	301227	334663	390147	426635	459596	488755	519205
Guizhou	61594	66152	79150	83103	97527	105277	117534	139211	166724	182189	196422	210279
Yunnan	106334	109895	119038	127560	140187	157143	173434	194707	210125	224899	237597	253555
Shaanxi	106391	110943	117851	125189	134431	142334	153847	169230	185139	199372	211885	225400
Gansu	63314	65988	70290	76581	81520	90410	94907	112296	116064	122412	127743	134346
Qinghai	15088	15470	16050	17352	19223	20451	23117	26018	29529	33007	34546	34749
Ningxia	17754	18260	18927	20891	22142	23659	25805	27765	31134	32506	33804	36313
Xinjiang	79441	83303	90329	96747	107243	116230	125391	131592	137325	142956	150263	156912

Employees of research and development institutions in various regions(Unit: people) (Data from China Science and Technology Statistical Yearbook)												
Region	2005	2006	2007	2008	2009	2010	2011	2012	2013	2014	2015	2016
Beijing	108991	112202	119441	124933	130687	138688	146738	156753	163135	168993	170563	167138
Tianjin	12341	12840	12945	11734	12603	12347	13536	14060	15036	16730	17088	17228
Hebei	12009	12271	14810	15035	15423	15935	17095	18121	19569	20347	21605	22900
Shanxi	14135	14234	15806	16475	16186	16364	16927	16876	16512	15913	16208	16479
nner Mongoli	8308	7637	7093	8227	8296	8437	8227	9213	9145	8679	9002	9597
Liaoning	21104	20325	20224	20065	20534	20640	21372	22039	21343	21599	21791	22144
Jilin	14092	13519	13204	13031	13307	13444	13167	13303	13216	12775	12520	12325
Heilongjiang	14071	14386	14381	14559	14435	14543	15168	14998	15242	15093	14593	13772
Shanghai	34086	35009	39059	38139	39136	40708	41491	44791	45650	47890	48056	44870
Jiangsu	29416	30280	34785	35148	38517	41559	43544	48709	51258	53827	57002	57211
Zhejiang	10310	10735	12957	13332	14872	16483	19888	22158	24790	27927	15775	15793
Anhui	12262	12991	14228	14331	15289	16075	18178	19109	20302	20360	21373	21987
Fujian	5246	5476	5778	5908	6287	6437	6408	6732	7127	7411	7469	7641
Jiangxi	12049	11869	11871	11809	11791	12011	12952	12788	12536	13379	13094	12732
Shandong	20987	21132	21262	21323	21503	22678	22822	23130	23093	22605	23079	22217
Henan	21726	20397	24015	25109	22855	23532	25404	27740	27179	28050	31241	29791
Hubei	23130	23880	25023	24997	25096	26168	29706	31448	31149	31764	32032	28356
Hunan	12615	12563	13018	13111	13158	15699	15873	15928	16824	15760	14010	13268
Guangdong	16328	16548	18682	18665	20426	20998	23173	25460	26774	28408	29124	29627
Guangxi	10346	10337	10282	10079	10283	10691	10487	11019	11123	11712	11986	11387
Hainan	4699	3931	4203	4285	4702	5077	5389	5454	5129	5165	4971	4681
Chongqing	7964	8099	8595	8630	9289	10247	12413	14030	15096	10604	11422	14120
Sichuan	54198	54526	60251	60899	63162	65709	69302	68001	71642	74240	77723	75444
Guizhou	6443	6260	6348	5686	5742	5437	5480	6242	5933	6255	6417	6429
Yunnan	9323	9599	9923	9856	10567	10784	10550	10857	10979	10502	11112	11995
Shaanxi	45857	45228	46466	48555	48817	51868	56476	58909	60853	62386	63004	59009
Gansu	11548	10624	11078	11115	11390	11660	10591	10822	10768	10949	10687	10631
Qinghai	863	881	896	914	933	1071	1040	1094	1133	1072	1364	1395
Ningxia	953	928	913	809	809	840	864	855	858	844	855	858
Xinjiang	6631	6637	6600	6715	6687	6780	7012	7115	7277	6392	6383	6494

Number of people who have not attended school(Unit: people) (Data from China Financial Yearbook)												
Region	2005	2006	2007	2008	2009	2010	2011	2012	2013	2014	2015	2016
Beijing	7697	596	490	472	440	363770	306	271	278	295	6180	313
Tianjin	6603	392	457	424	381	312312	307	297	292	375	5738	333
Hebei	58381	3665	3674	2939	2971	2153698	2317	2383	2177	2167	44629	2752
Shanxi	21630	1281	1191	1193	1234	868223	965	810	722	981	17212	909
nner Mongoli	31560	1872	1669	1631	1557	1131271	934	863	962	1024	21579	1013
Liaoning	25497	1690	1519	1481	1345	975536	943	908	809	817	15151	782

Jilin	20311	1270	1133	1094	978	637242	600	480	610	728	12954	675
Heilongjiang	30028	1718	1527	1433	1553	913586	935	873	818	992	18889	1295
Shanghai	12505	790	701	675	663	694616	509	466	758	653	11792	651
Jiangsu	96151	5982	5296	5081	4473	3448220	3396	3492	2629	3320	65122	3908
Zhejiang	71599	4322	4172	3928	3470	3372461	2912	2383	2412	2662	52902	2916
Anhui	125436	7647	7815	6726	6181	5158856	3780	3700	3394	3331	59704	3549
Fujian	52608	3200	3231	2990	2466	1190153	1530	1606	1679	1799	38145	1953
Jiangxi	48835	2930	2429	2226	1836	1682257	1468	1350	1081	1335	31679	1830
Shandong	140084	7206	6477	6200	5917	5326632	5427	4933	4288	4719	96731	5230
Henan	101730	6546	5969	5552	5278	4529564	4165	3958	3770	3541	77360	4323
Hubei	78240	4635	4152	3727	3764	2994419	2775	2697	2409	2666	51048	2690
Hunan	62996	3378	2996	3140	2886	2012174	2349	2338	1875	1837	36205	1984
Guangdong	67240	4023	3330	3268	2954	2611734	2845	2479	2620	3088	53925	3194
Guangxi	45454	2341	2241	2206	2094	1587939	1660	1433	1447	1537	32658	1697
Hainan	8748	596	550	550	510	390150	348	312	328	340	6574	332
Chongqing	37514	2188	1785	1740	1654	1348719	1264	1240	1199	1362	24737	1070
Sichuan	150815	8328	7039	6792	6276	4960433	4676	4353	4313	4591	97796	5508
Guizhou	83420	5146	4512	4014	3731	3313944	3033	3040	2657	2841	60459	3117
Yunnan	96592	5744	5467	4632	4849	3392628	3174	3009	2980	2977	63598	3219
Shaanxi	44316	2830	2737	2513	2283	1636786	1741	1576	1356	1790	29519	1685
Gansu	59141	4405	3866	3530	3300	2448945	2048	1774	1521	1790	41499	1793
Qinghai	14906	840	781	704	632	700487	534	639	597	587	13950	605
Ningxia	11607	699	617	458	476	419166	406	363	380	407	8397	350
Xinjiang	19703	1109	801	849	678	635247	679	629	694	666	16311	790

Number of junior high school graduates(Unit: people) (Data from China Financial Yearbook)												
Region	2005	2006	2007	2008	2009	2010	2011	2012	2013	2014	2015	2016
Beijing	63027	4058	4225	4499	4364	6157444	5201	4746	4496	4658	80272	4070
Tianjin	50103	3387	3546	3587	3785	4936137	4059	3990	4242	4158	85899	4169
Hebei	391553	28185	28441	28487	28577	31902985	28324	28314	26955	26964	468196	25765
Shanxi	192850	13859	13997	14052	13756	16115283	13699	13065	13129	13048	230264	12675
nner Mongol	114065	8353	8315	8741	8562	9689532	7977	8209	8374	8186	138368	7552
Liaoning	244140	16992	17137	17035	17546	19829444	16810	15616	15256	15573	293115	15399
Jilin	143398	10107	10382	10600	10607	11549212	9588	9955	9594	9189	174513	9333
Heilongjiang	211122	15111	15766	15695	15787	17245267	15248	14154	13761	13826	257250	13491
Shanghai	81520	5332	5490	5576	5506	8406458	7809	7728	7063	6729	120392	6199
Jiangsu	370726	25582	26528	27524	27275	30423013	26449	24602	24668	24496	425735	22540
Zhejiang	212998	15020	15389	15581	15624	19961827	17195	16056	15432	15978	284255	15554
Anhui	276259	20384	20393	20657	21334	22969845	20595	18814	18937	18727	352751	20802
Fujian	151251	10492	10268	10414	10122	13977601	11043	11267	11293	10598	189992	10337
Jiangxi	183396	12496	12901	13147	13650	16787837	14420	13805	13419	14194	252488	13502
Shandong	479871	33858	35648	35484	35933	38468023	33569	32127	32094	31654	569311	30835
Henan	539866	38637	38945	38750	38264	39925272	33086	34963	32606	30610	579625	32979
Hubei	276577	18850	19226	19150	19438	22677927	18884	17468	16684	16854	310274	17461
Hunan	308624	21835	22447	22059	22800	25977062	21941	21041	21498	21688	369554	20403
Guangdong	462511	33848	35053	34729	34816	44075971	35612	35639	37022	35212	630674	33330
Guangxi	215420	16332	17658	17974	17756	17840416	14642	15085	15385	15202	277465	16376
Hainan	41203	3120	3150	3196	3208	3642423	3295	3082	3169	3130	57970	3197
Chongqing	111705	8202	9094	9261	8594	9646397	7955	8224	8486	7386	146462	8112
Sichuan	309710	22388	24094	24676	23962	28058292	23843	23404	21140	22054	406996	23637
Guizhou	119830	9647	9921	10642	10924	10506809	9004	9289	9731	9483	174867	9951
Yunnan	132722	10808	10533	11108	11841	12591002	11906	11290	11631	12096	220696	12135
Shaanxi	181467	12723	12804	12665	12998	14981471	12936	12385	12128	11578	207427	11835
Gansu	92812	6963	7277	7283	7345	7982874	6803	6639	6945	6948	112808	6337
Qinghai	16520	1295	1269	1231	1186	1427740	1261	1242	1206	1223	22619	1390
Ningxia	22730	1682	1803	1918	1957	2130699	1955	1871	1825	1882	32026	1821
Xinjiang	87885	6463	6888	6894	7179	7906166	6419	6418	6697	6805	121585	6743

Population with college degree or above(Unit: people) (Data from China Financial Yearbook)												
Region	2005	2006	2007	2008	2009	2010	2011	2012	2013	2014	2015	2016
Beijing	48001	4042	4213	3986	4433	6177772	5597	6143	6859	6420	134106	7729
Tianjin	18601	1415	1498	1501	1713	2261701	2313	2553	2670	2727	53240	3176
Hebei	40036	2340	2454	2818	3233	5242511	3045	3232	4307	4447	108825	5966
Shanxi	23197	1949	2105	2092	2198	3114389	2347	2707	3013	2799	73661	3941
nner Mongol	23660	1367	1560	1539	1619	2522759	2532	2364	1966	2126	59527	3640
Liaoning	44404	3601	3765	4128	4371	5234081	4500	6519	6924	6013	110524	6331
Jilin	22832	1690	1790	1793	1913	2715172	2031	1955	2509	2578	53934	3093
Heilongjiang	30888	2074	2151	1980	2131	3492275	2945	3093	3708	3744	76013	4159
Shanghai	40549	3496	3457	3707	3855	5039565	4063	4392	4703	5156	102157	5791
Jiangsu	63909	4794	5355	4621	4995	8511408	7651	8373	8462	8796	190987	10458
Zhejiang	33115	3631	3727	4148	4335	5078506	5547	6473	7464	6510	118847	6705
Anhui	29007	2499	2054	2063	2360	4006203	3218	4721	4186	4862	103688	4515
Fujian	21877	1793	1741	1774	2918	3084680	3583	2262	2554	3366	70991	3421
Jiangxi	19946	1719	2638	2309	2447	3055988	2536	2846	3228	2801	68863	3177
Shandong	50909	4630	4652	4369	4728	8328681	6885	7367	7445	7415	179857	9499
Henan	48450	3331	3237	3703	4006	6016007	5612	4798	5757	7380	118304	5870
Hubei	36287	3885	4057	3965	3662	5456838	5181	5514	5304	5107	126939	6406
Hunan	34917	2777	3384	3519	3254	4991904	4114	3749	4343	4725	116158	6184
Guangdong	66510	4589	5207	5667	5512	8905508	8852	8027	6665	7693	186951	11779
Guangxi	22556	1820	1606	1308	1613	2751201	3172	2281	2732	2855	62166	2954
Hainan	5524	386	447	407	482	670160	526	694	593	555	14006	685

Chongqing	16122	1102	918	1019	1300	2445439	2696	2299	2162	2991	55464	3038
Sichuan	35297	3239	2901	3038	3844	5366709	5338	6258	6597	5706	131966	5869
Guizhou	14897	866	1026	1108	1035	1853345	2232	1749	2419	2763	42372	1905
Yunnan	18117	1184	1540	1334	1152	2636038	2558	2438	2763	2454	64678	3210
Shaanxi	28734	2445	2525	2802	2885	3940301	3050	3150	3463	3217	97441	3822
Gansu	13637	746	864	999	1053	1923282	1816	1790	1788	2061	47227	2191
Qinghai	4682	278	331	347	403	484794	405	423	553	567	8799	444
Ningxia	4895	370	380	390	425	587054	452	452	559	540	14533	801
Xinjiang	21340	1498	1567	1699	1658	2315302	2433	2272	2174	2263	47998	2483

RMB deposit balance of financial institutions(Unit: 100 million yuan) (Data from provincial and municipal statistical yearbooks)												
Region	2005	2006	2007	2008	2009	2010	2011	2012	2013	2014	2015	2016
Beijing	26785.9	31313.8	35369.7	42107.6	54275.5	64453.9	72655.4	81389.6	87990.6	95370.5	123767.4	132791.9
Tianjin	5716.09	6564.47	7930.31	9606.36	13548.56	16142.69	17197.51	19675.68	22684.59	23959.42	27145.93	29041.36
Hebei	10764.93	12551.62	14355.59	17709.02	22361.37	26099	29563.77	34257.16	39444.45	43764.02	48927.59	55928.87
Shanxi	7088.6971	8577.4569	10041.846	12766.718	15698.468	18575.653	20920.432	24050.581	26105.349	26779.47	28346.099	30371.374
nner Mongoli	3298.1538	4036.5605	4953.7024	6341.0312	8373.6999	10278.693	12063.722	13612.724	15205.693	16217.571	18077.597	21165.615
Liaoning	11966.9518	13596.7845	15117.796	18223.2	22758.6	27372.5	30832.4	35303.5	39418	42053.1	47758.2	51692.5
Jilin	4270.49	4963.71	53118.59	6362.48	8318	9606.7	10874.19	12706.13	14781.42	16400.1	18499.59	21003.9
Heilongjiang	6135.1	6923.4	7559.7	8993.8	11022.8	12835.7	14328.4	16326.6	18131.8	19254.8	21218.9	22179
Shanghai	21395.85	24428.61	28489.02	33643.85	44620.27	52190.04	58186.48	63555.25	69256.32	73882.45	103760.6	110510.96
Jiangsu	22001.44	25860.47	30450.54	37017.48	48850.29	58984.14	65723.56	75481.51	85604.08	93735.61	107873.03	121106.58
Zhejiang	20494.16	24413.94	28504.46	34806.43	44336.49	53441.45	59727.91	64886.28	71986.58	77145.38	87393.3	96438.16
Anhui	5993.82	7100.37	8406.57	10303.3	13306.53	16366.1	19404.3	22977.3	26739.3	29817.73	34482.9	40856.2
Fujian	7248.4	8836.26	10040.15	11804.4	14702.34	18309.45	21055.49	24283.68	28043.82	30747.61	35576.06	39275.82
Jiangxi	4445.4486	5213.7571	5900.0625	7206.5639	9296.3922	11846.183	14240.291	16715.907	19434.747	21537.74	24785.146	28893.105
Shandong	17103.5148	19633.9878	22072.243	26930.181	34697.776	41104.965	46345.413	54301.525	62077.881	67498.288	74524.157	83414.88
Henan	10003.96	11492.55	12576.42	15255.42	19175.06	23148.83	26646.15	31970.43	37591.7	41374.91	47629.91	53977.62
Hubei	8184.96	9570.97	11093.03	13439.52	17505.86	21568.31	23949.15	28006.36	32636.15	36153.65	40896.52	47284.95
Hunan	6498.2262	7719.4294	9083.2721	10895.495	13947.997	16643.271	19444.097	23147.148	26876.027	30255.58	36220.614	41996.738
Guangdong	35958.71	41146.58	47016.48	54309.57	67742.59	79957.97	89168.6	99934.6	114855.02	121964.85	153551.79	171024.47
Guangxi	4262.3	5029.47	5801.04	7075.02	9638.89	11813.9	13527.97	15966.65	18400.48	20298.54	22793.54	25477.8
Hainan	1262.1622	1513.6894	1833.2099	2305.4593	3107.262	4166.0283	4449.9859	5042.8256	5878.5984	6363.572	7508.84	8959.21
Chongqing	4727.72	5519.75	6576.68	8021.95	10933	13454.98	15832.81	18934.83	22202.1	24501.542	28094.37	31216.45
Sichuan	9905.33	11802.14	13980.36	18661.04	24976.45	30299.67	34971.21	41576.8	48122.05	53935.75	60117.72	66892.423
Guizhou	2777.54	3300.08	3826.37	4736.93	5898.26	7363.92	8742.79	10540.06	13265.01	15263.258	19438.64	23770.931
Yunnan	5140.5	6131.25	7170.87	8418.94	11119.64	13411.49	15416.7	18008.04	20767.77	22456.26	25035.09	27726.1
Shaanxi	6446.4787	7452.5296	8501.3871	10790.866	13860.431	16386.18	19147.619	22657.74	25577.19	28111.34	32415.24	35255.48
Gansu	2895.861	3316.9583	3747.1108	4728.8246	5881.8151	7115.3682	8394.04	10033.4	12029.66	13921.36	16141.19	17411.68
Qinghai	733.0545	896.7816	1092.6494	1383.6757	1785.7812	2319.6433	2825.83	3528.41	4102.54	4529.87	5212.8	5570.17
Ningxia	985.34	1131.22	1278.52	1590.58	2058.49	2573.64	2966.87	3495.41	3868.47	4209.06	4805.15	5441.54
Xinjiang	3427.48	4040.78	4614.62	5399.34	6845.07	8870.72	10387	12330.89	14088.83	15055.39	17123.95	18747.64

Urban registered unemployment rate(Unit: %) (Data from provincial and municipal statistical yearbooks)												
Region	2005	2006	2007	2008	2009	2010	2011	2012	2013	2014	2015	2016
Beijing	2.1	2	1.8	1.8	1.4	1.4	1.39	1.27	1.21	1.31	1.39	1.4
Tianjin	3.7	3.6	3.6	3.6	3.6	3.6	3.6	3.6	3.6	3.6	3.5	3.5
Hebei	3.9	3.8	3.8	4	3.9	3.86	3.75	3.69	3.68	3.59	3.6	3.7
Shanxi	3	3.2	3.2	3.3	3.9	3.6	3.5	3.3	3.1	3.4	3.5	3.5
nner Mongoli	4.3	4.1	4	4.1	4	3.9	3.8	3.7	3.7	3.6	3.7	3.7
Liaoning	5.7	5.1	4.4	3.8	3.9	3.7	3.7	3.6	3.4	3.4	3.4	3.8
Jilin	4.2	4.2	3.9	4	4	3.8	3.7	3.7	3.7	3.4	3.5	3.5
Heilongjiang	4.4	4.3	4.3	4.2	4.3	4.27	4.1	4.15	4.43	4.47	4.48	4.22
Shanghai	4.4	4.4	4.2	4.2	4.3	4.4	3.5	3.1	4	4.1	4	4.1
Jiangsu	3.6	3.4	3.19	3.25	3.22	3.16	3.22	3.14	3.03	3.01	3	3
Zhejiang	3.71	3.5	3.3	3.5	3.3	2.19	1.86	1.63	3	3	2.9	2.9
Anhui	4.4	4.2	4.06	3.92	3.92	3.7	3.74	3.68	3.41	3.21	3.14	3.2
Fujian	4	3.93	3.9	3.86	3.9	3.77	3.69	3.63	3.55	3.47	3.66	3.86
Jiangxi	3.5	3.6	3.4	3.4	3.4	3.3	3	3	3.2	3.3	3.4	3.4
Shandong	3.3	3.3	3.2	3.7	3.4	3.4	3.4	3.3	3.2	3.3	3.4	3.5
Henan	3.5	3.5	3.4	3.4	3.5	3.4	3.4	3.1	3.1	3	3	3
Hubei	4.33	4.22	4.21	4.2	4.21	4.18	4.1	3.83	3.49	3.1	2.64	2.41
Hunan	4.3	4.3	4.3	4.2	4.1	4.2	4.2	4.2	4.2	4.1	4.1	4.2
Guangdong	2.6	2.6	2.5	2.6	2.6	2.5	2.5	2.5	2.4	2.4	2.5	2.5
Guangxi	4.2	4.1	3.8	3.8	3.7	3.7	3.5	3.4	3.3	3.2	2.9	2.9
Hainan	3.6	3.6	3.5	3.7	3.5	3	1.7	2	2.2	2.3	2.3	2.4
Chongqing	4.1	4	4	4	4	3.9	3.5	3.3	3.4	3.5	3.6	3.7
Sichuan	4.6	4.5	4.2	4.6	4.3	4.1	4.2	4	4.1	4.2	4.1	4.2
Guizhou	4.2	4.1	4	4	3.8	3.6	3.6	3.3	3.3	3.3	3.3	3.2
Yunnan	4.2	4.3	4.2	4.2	4.3	4.2	4.1	4	4	4	4	3.6
Shaanxi	4.2	4.03	4.02	3.91	3.94	3.9	3.59	3.22	3.32	3.41	3.4	3.3
Gansu	3.3	3.6	3.3	3.2	3.3	3.2	3.1	2.7	2.3	2.2	2.1	2.2
Qinghai	3.9	3.9	3.8	3.8	3.8	3.8	3.8	3.4	3.3	3.2	3.2	3.1
Ningxia	4.5	4.3	4.3	4.4	4.4	4.4	4.4	4.2	4.1	4	4	3.9
Xinjiang	3.9	3.9	3.9	3.7	3.8	3.2	3.2	3.4	3.4	3.2	2.9	2.5

Gross regional product per capita (RMB per person) (Data from China Statistical Yearbook)												
Region	2005	2006	2007	2008	2009	2010	2011	2012	2013	2014	2015	2016
Beijing	45443.6929	50467.00	58204.00	63029.00	70452.00	75943.00	81658	87475	94648	99995	106497	118198
Tianjin	35783.1885	41163.00	46122.00	55473.00	62574.00	72994.00	85213	93173	100105	105231	107960	115053
Hebei	14782.2597	16962.00	19877.00	23239.00	24581.00	28668.00	33969	36584	38909	39984	40255	43062
Shanxi	12495	14123.00	16945.00	20398.00	21522.00	26283.00	31357	33628	34984	35070	34919	35532
ner Mongol	16330.8187	20053.00	25393.00	32214.00	40282.00	47347.00	57974	63886	67836	71046	71101	72064
Liaoning	18983.2013	21788.00	25729.00	31259.00	35239.00	42355.00	50760	56649	61996	65201	65354	50791
Jilin	13348	15720.00	19383.00	23514.00	26595.00	31599.00	38460	43415	47428	50160	51086	53868
Heilongjian	14434.0561	16195.00	18478.00	21727.00	22447.00	27076.00	32819	35711	37697	39226	39462	40432
Shanghai	51474	57695.00	66367.00	73124.00	78989.00	76074.00	82560	85373	90993	97370	103796	116562
Jiangsu	24560	28814.00	33928.00	39622.00	44744.00	52840.00	62290	68347	75354	81874	87995	96887
Zhejiang	27702.6847	31874.00	37411.00	42214.00	44641.00	51711.00	59249	63374	68805	73002	77644	84916
Anhui	8675.14475	10055.00	12045.00	14485.00	16407.66	20888.00	25659	28792	32001	34425	35997	39561
Fujian	18645.8416	21471.00	25908.00	30123.00	33840.00	40025.00	47377	52763	58145	63472	67966	74707
Jiangxi	9440	10798.00	12633.00	14781.00	17335.00	21253.00	26150	28800	31930	34674	36724	40400
Shandong	20096.4547	23794.00	27807.00	33083.00	35894.00	41106.00	47335	51768	56885	60879	64168	68733
Henan	11346.498	13313.00	16012.00	19593.00	20597.00	24446.00	28661	31499	34211	37072	39123	42575
Hubei	11431	13296.00	16206.00	19860.00	22677.00	27906.00	34197	38572	42826	47145	50654	55665
Hunan	10426	11950.00	14492.00	17521.00	20428.00	24719.00	29880	33480	36943	40271	42754	46382
Guangdong	24435.0156	28332.00	33151.00	37589.00	41166.00	44736.00	50807	54095	58833	63469	67503	74016
Guangxi	8787.72899	10296.00	12555.00	14966.00	16045.00	20219.00	25326	27952	30741	33090	35190	38027
Hainan	10871	12654.00	14555.00	17175.00	19254.00	23831.00	28898	32377	35663	38924	40818	44347
Chongqing	10982	12457.00	14660.00	18025.00	22920.00	27596.00	34500	38914	43223	47850	52321	58502
Sichuan	9060	10546.00	12893.00	15378.00	17339.00	21182.00	26133	29608	32617	35128	36775	40003
Guizhou	5051.96	5787.00	6915.00	8824.00	10309.00	13119.00	16413	19710	23151	26437	29847	33246
Yunnan	7835	8970.00	10540.00	12587.00	13539.00	15752.00	19265	22195	25322	27264	28806	31093
Shaanxi	9899	12138.00	14607.00	18246.00	21688.00	27133.00	33464	38564	43117	46929	47626	51015
Gansu	7476.52929	8757.00	10346.00	12110.00	12872.00	16113.00	19595	21978	24539	26433	26165	27643
Qinghai	10044.7403	11762.00	14257.00	17389.00	19454.00	24115.00	29522	33181	36875	39671	41252	43531
Ningxia	10239	11847.00	14649.00	17892.00	21777.00	26860.00	33043	36394	39613	41834	43805	47194
Xinjiang	13108	15000.00	16999.00	19893.00	19942.00	25034.00	30087	33796	37553	40648	40036	40564

Consumer Price Index(%) (Data from China Statistical Yearbook)												
Region	2005	2006	2007	2008	2009	2010	2011	2012	2013	2014	2015	2016
Beijing	101.45	100.89	102.41	105.06	98.46	102.40	105.6	103.3	103.3	101.6	101.8	101.4
Tianjin	101.55	101.51	104.22	105.40	99.03	103.55	104.9	102.7	103.1	101.9	101.7	102.1
Hebei	101.80	101.69	104.68	106.20	99.32	103.07	105.7	102.6	103	101.7	100.9	101.5
Shanxi	102.30	102.03	104.63	107.19	99.58	103.02	105.2	102.5	103.1	101.7	100.6	101.1
nner Mongoli	102.44	101.48	104.61	105.74	99.73	103.20	105.6	103.1	103.2	101.6	101.1	101.2
Liaoning	101.36	101.22	105.12	104.64	100.05	103.04	105.2	102.8	102.4	101.7	101.4	101.6
Jilin	101.50	101.36	104.80	105.11	100.07	103.70	105.2	102.5	102.9	102	101.7	101.6
Heilongjiang	101.18	101.93	105.39	105.56	100.19	103.90	105.8	103.2	102.2	101.5	101.1	101.5
Shanghai	100.96	101.21	103.15	105.78	99.59	103.11	105.2	102.8	102.3	102.7	102.4	103.2
Jiangsu	102.11	101.62	104.31	105.36	99.57	103.81	105.3	102.6	102.3	102.2	101.7	102.3
Zhejiang	101.32	101.06	104.17	105.03	98.47	103.84	105.4	102.2	102.3	102.1	101.4	101.9
Anhui	101.35	101.22	105.27	106.17	99.09	103.14	105.6	102.3	102.4	101.6	101.3	101.8
Fujian	102.22	100.80	105.23	104.55	98.19	103.22	105.3	102.4	102.5	102	101.7	101.7
Jiangxi	101.72	101.17	104.84	106.05	99.34	103.04	105.2	102.7	102.5	102.3	101.5	102
Shandong	101.68	101.01	104.44	105.29	100.00	102.93	105	102.1	102.2	101.9	101.2	102.1
Henan	102.08	101.29	105.42	107.03	99.42	103.53	105.6	102.5	102.9	101.9	101.3	101.9
Hubei	102.90	101.58	104.84	106.27	99.59	102.91	105.8	102.9	102.8	102	101.5	102.2
Hunan	102.32	101.44	105.57	106.02	99.64	103.11	105.5	102	102.5	101.9	101.4	101.9
Guangdong	102.25	101.79	103.68	105.60	97.65	103.12	105.3	102.8	102.5	102.3	101.5	102.3
Guangxi	102.44	101.34	106.09	107.78	97.86	102.98	105.9	103.2	102.2	102.1	101.5	101.6
Hainan	101.46	101.53	105.04	106.93	99.32	104.84	106.1	103.2	102.8	102.4	101	102.8
Chongqing	100.76	102.37	104.73	105.57	98.36	103.25	105.3	102.6	102.7	101.8	101.3	101.8
Sichuan	101.66	102.32	105.93	105.07	100.80	103.18	105.3	102.5	102.8	101.6	101.5	101.9
Guizhou	101.01	101.71	106.41	107.59	98.72	102.92	105.1	102.7	102.5	102.4	101.8	101.4
Yunnan	101.43	101.87	105.86	105.70	100.40	103.73	104.9	102.7	103.1	102.4	101.9	101.5
Shaanxi	101.17	101.48	105.15	106.36	100.52	103.97	105.7	102.8	103	101.6	101	101.3
Gansu	101.75	101.28	105.53	108.21	101.25	104.10	105.9	102.7	103.2	102.1	101.6	101.3
Qinghai	100.76	101.59	106.64	110.09	102.65	105.35	106.1	103.1	103.9	102.8	102.6	101.8
Ningxia	101.50	101.94	105.41	108.48	100.75	104.07	106.3	102	103.4	101.9	101.1	101.5
Xinjiang	100.73	101.31	105.49	108.06	100.74	104.33	105.9	103.8	103.9	102.1	100.6	101.4

Number of patents granted(Unit: piece) (Data from China Statistical Yearbook)												
Region	2005	2006	2007	2008	2009	2010	2011	2012	2013	2014	2015	2016
Beijing	10100	11238	14954	17747	22921	33511	40888	50511	62671	74661	94031	100578
Tianjin	3045	4159	5584	6790	7404	11006	13982	19782	24856	26351	37342	39734
Hebei	3585	4131	5358	5496	6839	10061	11119	15315	18186	20132	30130	31826
Shanxi	1220	1421	1992	2279	3227	4752	4974	7196	8565	8371	10020	10062
nner Mongoli	845	978	1313	1328	1494	2096	2262	3084	3836	4031	5522	5846
Liaoning	6195	7399	9615	10665	12198	17093	19176	21223	21656	19525	25182	25104
Jilin	2023	2319	2855	2984	3275	4343	4920	5930	6219	6696	8878	9995
Heilongjiang	2906	3622	4303	4574	5079	6780	12236	20268	19819	15412	18943	18046
Shanghai	12603	16602	24481	24468	34913	48215	47960	51508	48680	50488	60623	64230
Jiangsu	13580	19352	31770	44438	87286	138382	199814	269944	239645	200032	250290	231033
Zhejiang	19056	30968	42069	52953	79945	114643	130190	188463	202350	188544	234983	221456
Anhui	1939	2235	3413	4346	8594	16012	32681	43321	48849	48380	59039	60983
Fujian	5147	6412	7761	7937	11282	18063	21857	30497	37511	37857	61621	67142

Jiangxi	1361	1536	2069	2295	2915	4349	5550	7985	9970	13831	24161	31472
Shandong	10743	15937	22821	26688	34513	51490	58844	75496	76976	72818	98101	98093
Henan	3748	5242	6998	9133	11425	16539	19259	26791	29482	33366	47766	49145
Hubei	3860	4734	6616	8374	11357	17362	19035	24475	28760	28290	38781	41822
Hunan	3659	5608	5687	6133	8309	13873	16064	23212	24392	26637	34075	34050
Guangdong	36894	43516	56451	62031	83621	119343	128413	153598	170430	179953	241176	259032
Guangxi	1225	1442	1907	2228	2702	3647	4402	5900	7884	9664	13573	14858
Hainan	200	248	296	341	630	714	765	1093	1331	1597	2061	1939
Chongqing	3591	4590	4994	4820	7501	12080	15525	20364	24828	24312	38914	42738
Sichuan	4606	7138	9935	13369	20132	32212	28446	42218	46171	47120	64953	62445
Guizhou	925	1337	1727	1728	2084	3086	3386	6059	7915	10107	14115	10425
Yunnan	1381	1637	2139	2021	2923	3823	4199	5853	6804	8124	11658	12032
Shaanxi	1894	2473	3451	4392	6087	10034	11662	14908	20836	22820	33350	48455
Gansu	547	832	1025	1047	1274	1868	2383	3662	4737	5097	6912	7975
Qinghai	79	97	222	228	368	264	538	527	502	619	1217	1357
Ningxia	214	290	296	606	910	1081	613	844	1211	1424	1865	2677
Xinjiang	921	1187	1534	1493	1866	2562	2642	3439	4998	5238	8761	7116

Population with college degree or above(Unit: 10000 yuan) (Data from China Statistical Yearbook, China Population and Employment Statistical Yearbook)												
Region	2005	2006	2007	2008	2009	2010	2011	2012	2013	2014	2015	2016
Beijing	48001	4042	4213	3986	4433	6177772	5597	6143	6859	6420	134106	7729
Tianjin	18601	1415	1498	1501	1713	2261701	2313	2553	2670	2727	53240	3176
Hebei	40036	2340	2454	2818	3233	5242511	3045	3232	4307	4447	108825	5966
Shanxi	23197	1949	2105	2092	2198	3114389	2347	2707	3013	2799	73661	3941
nner Mongoli	23660	1367	1560	1539	1619	2522759	2532	2364	1966	2126	59527	3640
Liaoning	44404	3601	3765	4128	4371	5234081	4500	6519	6924	6013	110524	6331
Jilin	22832	1690	1790	1793	1913	2715172	2031	1955	2509	2578	53934	3093
Heilongjiang	30888	2074	2151	1980	2131	3492275	2945	3093	3708	3744	76013	4159
Shanghai	40549	3496	3457	3707	3855	5039565	4063	4392	4703	5156	102157	5791
Jiangsu	63909	4794	5355	4621	4995	8511408	7651	8373	8462	8796	190987	10458
Zhejiang	33115	3631	3727	4148	4335	5078506	5547	6473	7464	6510	118847	6705
Anhui	29007	2499	2054	2063	2360	4006203	3218	4721	4186	4862	103688	4515
Fujian	21877	1793	1741	1774	2918	3084680	3583	2262	2554	3366	70991	3421
Jiangxi	19946	1719	2638	2309	2447	3055988	2536	2846	3228	2801	68863	3177
Shandong	50909	4630	4652	4369	4728	8328681	6885	7367	7445	7415	179857	9499
Henan	48450	3331	3237	3703	4006	6016007	5612	4798	5757	7380	118304	5870
Hubei	36287	3885	4057	3965	3662	5456838	5181	5514	5304	5107	126939	6406
Hunan	34917	2777	3384	3519	3254	4991904	4114	3749	4343	4725	116158	6184
Guangdong	66510	4589	5207	5667	5512	8905508	8852	8027	6665	7693	186951	11779
Guangxi	22556	1820	1606	1308	1613	2751201	3172	2281	2732	2855	62166	2954
Hainan	5524	386	447	407	482	670160	526	694	593	555	14006	685
Chongqing	16122	1102	918	1019	1300	2445439	2696	2299	2162	2991	55464	3038
Sichuan	35297	3239	2901	3038	3844	5366709	5338	6258	6597	5706	131966	5869
Guizhou	14897	866	1026	1108	1035	1853345	2232	1749	2419	2763	42372	1905
Yunnan	18117	1184	1540	1334	1152	2636038	2558	2438	2763	2454	64678	3210
Shaanxi	28734	2445	2525	2802	2885	3940301	3050	3150	3463	3217	97441	3822
Gansu	13637	746	864	999	1053	1923282	1816	1790	1788	2061	47227	2191
Qinghai	4682	278	331	347	403	484794	405	423	553	567	8799	444
Ningxia	4895	370	380	390	425	587054	452	452	559	540	14533	801
Xinjiang	21340	1498	1567	1699	1658	2315302	2433	2272	2174	2263	47998	2483

Total fixed capital formation(Unit: 100 million yuan) (Data from China Statistical Yearbook)												
Region	2005	2006	2007	2008	2009	2010	2011	2012	2013	2014	2015	2016
Beijing	3204.65	3551.17	4082.56	3754.49	4435	5342.4	5953.9	7032.8	7595.4	7957.2	8155.4	9716.1
Tianjin	1753.92	2122.35	2681.35	3746.99	5077.9	6468.5	8069.9	9314.8	10438.8	11338	10495.8	10113.9
Hebei	4239.12	5094.34	6211.94	8397.68	9390.2	10791.6	13688.8	15087.9	16167.1	17064.7	17298.8	18595.7
Shanxi	1897.39	2345.57	2909.61	3587.61	4856.8	5973.1	7256.1	7663.4	8693.5	8818.5	8821.6	8369.4
nner Mongoli	2685.22	3353.88	4356.39	5596.45	7425.2	8938.7	10837.1	12954.3	15287.1	13453.9	13844.4	12489.5
Liaoning	3705.99	4755.78	6031.3	9981.43	8906.4	11024.6	13474.6	15049.6	16479.8	16927.4	12098.9	9171.7
Jilin	1743.66	2804.29	4003.18	5608.3	6280.5	7618.1	8355.5	9235	9751.5	10372.7	11001.2	10185.7
Heilongjiang	1788.67	2232.04	2877.84	3655.29	4995	5410.5	6599.3	7824.1	9153.5	9288	9664.1	9151.4
Shanghai	3743.42	4272.85	5041.4	5419.43	6447.5	6380.3	6801.8	7012.5	7617.1	8022.5	8999.9	10631.4
Jiangsu	8739.71	10021.43	11594.62	14418.47	17138	20709.1	24522.2	26415.5	27711.1	28796.2	29940.8	32254.6
Zhejiang	6269.16	7065.65	8201.28	9369.14	10220.1	12101.3	13822.9	14607.6	16139.7	17000.9	18213.3	20677.8
Anhui	2224.31	2682.62	3346.26	4290.19	4820.5	6061.1	7594.4	8680.9	9739.1	10723.8	11106.5	12345.1
Fujian	2654.95	3310.15	4344.88	5601.36	6438.3	7341.6	9060.5	10270.2	11678.6	13038	14140.3	15576.1
Jiangxi	1922.1	2282.41	2688.33	3203.51	4082.6	4740.3	5785.8	6301.1	6774.2	6876.9	7706	9029.5
Shandong	8798.79	10408.84	11784.57	14141.48	17734.4	20800.6	24281.2	26808.9	29249.5	31647.1	33229.4	33548.7
Henan	4506.75	6007.32	8043.35	10435.38	12996.1	15704.1	18819.2	21667.8	24376	26655.8	27722.5	29595.1
Hubei	2804.01	3542.47	4371.14	5368.99	6612.9	8200.4	10597.8	12064.8	13701.9	15442.9	16757.4	18515.8
Hunan	2569.24	3125.38	3945.45	5480.69	6666.8	8568.8	10487.3	11990.7	13573.9	15139.7	15085	16477.4
Guangdong	7407.54	8465.26	9920.99	11405.38	14025.1	16812.7	19432.8	22033.8	24997.7	27930.8	29250.4	33279.7
Guangxi	1699.04	2201.15	2831.62	3559.92	5533.2	7785.5	9745.7	10547.4	9725.6	10463.1	11264.7	12114.1
Hainan	363.37	423.88	495.5	741.07	895.9	1179.4	1460.5	1947.9	2233.5	2549.3	2325.1	2519.1
Chongqing	1862.65	2138.2	2587.86	3615.03	3633.2	4379.3	5511.2	6041.2	6581	7380.9	8042.5	9114.9
Sichuan	3179.92	3989.63	5005.45	6579.56	7464.2	8911.1	10691.3	12096.2	13081.7	13990.6	14415.3	15800.5
Guizhou	996.24	1152.95	1361.79	1689.06	2046.8	2510.4	3147.5	4067.3	5141.8	5928.8	6913.9	7975.5
Yunnan	1755.3	2156.84	2616.77	2616.77	3502.4	5213.1	6678.2	7949.5	9311.1	10918.8	12080.1	13321.1
Shaanxi	2035.2	2659.37	3152.28	4569.16	5270.3	6851.5	8190.2	9700.1	10779.5	11783.7	11662.8	12584.8

Gansu	874.52	1027.78	1221.96	1733.54	1788.3	2177.9	2685.8	3128.7	3649.1	4116.7	4412.3	4894
Qinghai	364.08	411.68	480.43	583.2	791.8	1057.9	1369.9	1895.7	2418.3	2953.5	3303.6	3551.9
Ningxia	444.82	515.26	621.82	858.64	1185.4	1490	1653.2	1967.2	2223	2987.7	3516.9	3757.1
Xinjiang	1485.17	1825.75	2005	2227.63	2473.2	3233	3948.9	5477.6	6943.7	8301.4	8755.7	8655.1

Total factor productivity(Calculated by DEA- Malmquist index method)												
Region	2005	2006	2007	2008	2009	2010	2011	2012	2013	2014	2015	2016
Beijing	0.999	1.065	1.001	1.182	0.954	1.003	1.034	0.964	1.028	1.038	1.021	0.992
Tianjin	1.061	0.961	0.938	1.074	1.061	1.101	1.116	1.055	1.030	1.032	1.011	1.053
Hebei	0.963	0.953	0.965	0.913	0.945	1.038	0.947	0.961	0.969	0.966	0.971	0.980
Shanxi	0.914	0.928	0.995	1.041	0.750	1.028	1.009	1.008	0.898	0.973	0.976	1.066
nner Mongol	0.951	1.071	1.034	1.104	1.026	1.119	1.105	1.027	0.956	0.980	1.007	1.036
Liaoning	1.047	0.966	0.983	0.841	1.178	0.987	0.999	0.983	0.991	1.011	1.326	0.999
Jilin	0.803	0.816	0.927	0.939	1.038	1.015	1.119	1.076	1.014	1.001	0.960	1.089
Heilongjiang	0.994	0.904	0.880	0.952	0.751	1.129	1.011	0.896	0.884	1.010	0.944	1.055
Shanghai	1.077	1.047	1.073	1.049	1.017	1.098	1.056	0.999	0.943	1.017	0.937	0.961
Jiangsu	1.035	1.048	1.067	1.025	0.934	1.012	1.016	0.982	1.035	1.037	0.980	0.989
Zhejiang	1.033	1.066	1.040	1.047	0.973	1.031	1.041	0.985	0.964	0.995	0.957	0.948
Anhui	0.946	0.954	0.967	0.966	0.980	0.998	1.011	0.972	0.974	0.972	0.975	0.972
Fujian	0.958	0.972	0.985	0.953	0.993	1.059	0.974	0.970	0.953	0.970	0.963	0.990
Jiangxi	1.009	1.021	1.027	1.050	0.834	1.081	1.045	0.999	1.014	1.051	0.906	0.926
Shandong	1.006	1.030	1.046	1.033	0.864	0.995	1.010	0.986	0.994	0.979	0.975	1.038
Henan	0.893	0.879	0.900	0.942	0.843	0.981	0.990	0.941	0.939	0.974	0.981	0.997
Hubei	0.960	0.917	0.986	1.017	0.922	1.011	0.965	0.985	0.959	0.970	0.974	0.980
Hunan	0.903	0.974	0.975	0.913	0.930	0.964	1.018	0.982	0.970	0.981	1.062	0.985
Guangdong	0.974	1.035	1.024	1.042	0.878	0.975	1.003	0.934	0.955	0.964	1.000	0.957
Guangxi	0.876	0.918	0.920	0.960	0.711	0.878	0.982	1.002	1.178	1.003	0.969	0.993
Hainan	0.950	0.992	1.037	0.849	0.896	0.951	0.990	0.839	0.937	0.948	1.141	0.984
Chongqing	1.090	0.975	0.996	0.949	1.116	0.997	1.010	1.032	1.006	0.980	0.980	0.968
Sichuan	0.963	0.943	0.958	0.972	0.965	1.011	1.019	0.989	0.998	1.000	0.986	0.979
Guizhou	1.058	1.002	1.015	1.008	0.923	0.957	0.991	0.919	0.919	0.981	0.939	0.945
Yunnan	0.893	0.937	0.971	1.212	0.791	0.779	0.958	0.962	0.961	0.911	0.934	0.971
Shaanxi	1.029	0.933	1.013	0.904	0.966	0.954	1.037	0.973	0.999	0.994	1.007	0.984
Gansu	0.842	1.030	0.973	0.815	1.039	0.993	0.977	0.960	0.935	0.939	0.891	0.931
Qinghai	1.035	1.064	1.029	1.056	0.779	0.927	0.960	0.960	0.936	1.028	1.010	1.036
Ningxia	1.000	1.031	1.061	1.010	0.828	1.000	1.134	0.931	0.951	0.940	1.032	1.052
Xinjiang	1.030	1.001	1.051	1.094	0.904	0.982	1.007	0.793	0.860	0.962	0.936	1.031

Per capita disposable income of rural residents(Unit: yuan) (Data from China Statistical Yearbook)												
Region	2005	2006	2007	2008	2009	2010	2011	2012	2013	2014	2015	2016
Beijing	7346.25918	8275.47	9439.63	10661.92	11668.59	13262.29	14735.68	16475.74	17101.2	18867.3	20568.7	22309.5
Tianjin	5579.87026	6227.94	7010.06	7910.78	8687.56	10074.86	12321.22	14025.54	15352.6	17014.2	18481.6	20075.6
Hebei	3481.63786	3801.82	4293.43	4795.46	5149.67	5957.98	7119.69	8081.39	9187.7	10186.1	11050.5	11919.4
Shanxi	2890.65614	3180.92	3665.66	4097.24	4244.1	4736.25	5601.4	6356.63	7949.5	8809.4	9453.9	10082.5
nner Mongol	2988.86971	3341.88	3953.1	4656.18	4937.8	5529.59	6641.56	7611.31	8984.9	9976.3	10775.9	11609
Liaoning	3690.20622	4090.4	4773.43	5576.48	5958	6907.93	8296.54	9383.72	10161.2	11191.5	12056.9	12880.7
Jilin	3263.99003	3641.13	4191.34	4932.74	5265.91	6237.44	7509.95	8598.17	9780.7	10780.1	11326.2	12122.9
Heilongjiang	3221.26894	3552.43	4132.29	4855.59	5206.76	6210.72	7590.68	8603.85	9369	10453.2	11095.2	11831.9
Shanghai	8247.77151	9138.65	10144.62	11440.26	12482.94	13977.96	16053.79	17803.68	19208.3	21191.6	23205.2	25520.4
Jiangsu	5276.28872	5813.23	6561.01	7356.47	8003.54	9118.24	10804.95	12201.95	13521.3	14958.4	16256.7	17605.6
Zhejiang	6659.95114	7334.81	8265.15	9257.93	10007.31	11302.55	13070.69	14551.92	17493.9	19373.3	21125	22866.1
Anhui	2640.96379	2969.08	3556.27	4202.49	4504.32	5285.17	6232.21	7160.46	8850	9916.4	10820.7	11720.5
Fujian	4450.35572	4834.75	5467.08	6196.07	6680.18	7426.86	8778.55	9967.17	11404.8	12650.2	13792.7	14999.2
Jiangxi	3128.88771	3459.53	4044.7	4697.19	5075.01	5788.56	6891.63	7829.43	9088.8	10116.6	11139.1	12137.7
Shandong	3930.54821	4368.33	4985.34	5641.43	6118.77	6990.28	8342.13	9446.54	10686.9	11882.3	12930.4	13954.1
Henan	2870.58261	3261.03	3851.6	4454.24	4806.95	5523.73	6604.03	7524.94	8969.1	9966.1	10852.9	11696.7
Hubei	3099.20064	3419.35	3997.48	4656.38	5035.26	5832.27	6897.92	7851.71	9691.8	10849.1	11843.9	12725
Hunan	3117.74494	3389.62	3904.2	4512.46	4909.04	5621.96	6567.06	7440.17	9028.6	10060.2	10992.5	11930.4
Guangdong	4690.48652	5079.78	5624.04	6399.79	6906.93	7890.25	9371.73	10542.84	11067.8	12245.6	13360.4	14512.2
Guangxi	2494.66677	2770.48	3224.05	3690.34	3980.44	4543.41	5231.33	6007.55	7793.1	8683.2	9466.6	10359.5
Hainan	3004.0319	3255.53	3791.37	4389.97	4744.36	5275.37	6446.01	7408	8801.7	9912.6	10857.6	11842.9
Chongqing	2809.31893	2873.83	3509.29	4126.21	4478.35	5276.66	6480.41	7383.27	8492.5	9489.8	10504.7	11548.8
Sichuan	2802.77646	3002.38	3546.69	4121.21	4462.05	5086.89	6128.55	7001.43	8380.7	9347.7	10247.4	11203.1
Guizhou	1876.95807	1984.62	2373.99	2796.93	3005.41	3471.93	4145.35	4753	5897.8	6671.2	7386.9	8090.3
Yunnan	2041.79326	2250.46	2634.09	3102.6	3369.34	3952.03	4721.99	5416.54	6723.6	7456.1	8242.1	9019.8
Shaanxi	2052.62911	2260.19	2644.69	3136.46	3437.55	4104.98	5027.87	5762.52	7092.2	7932.2	8688.9	9396.4
Gansu	1979.87941	2134.05	2328.92	2723.79	2980.1	3424.65	3909.37	4506.66	5588.8	6276.6	6936.2	7456.9
Qinghai	2151.4639	2358.37	2683.78	3061.24	3346.15	3862.68	4608.46	5364.38	6461.6	7282.7	7933.4	8664.4
Ningxia	2508.88659	2760.14	3180.84	3681.42	4048.33	4674.89	5409.95	6180.32	7598.7	8410	9118.7	9851.6
Xinjiang	2482.15262	2737.28	3182.97	3502.9	3883.1	4642.67	5442.15	6393.68	7846.6	8723.8	9425.1	10183.2

Per capita food consumption expenditure of urban residents(Unit: yuan) (Data from China Statistical Yearbook)												
Region	2005	2006	2007	2008	2009	2010	2011	2012	2013	2014	2015	2016
Beijing	4215.56	4560.52	4934.05	5561.54	5936.11	6392.9	6905.51	7535.29	8170.22	7467.8	8091.1	8070.4
Tianjin	3542.9	3680.22	4249.31	5005.09	5404.53	5940.44	6663.31	7343.64	7943.06	7376.6	8447.7	8679.6
Hebei	2315.76	2492.26	2789.85	3155.4	3250.77	3335.23	3927.26	4211.16	4404.93	3263.7	4581.1	4991.6
Shanxi	2056.79	2252.5	2600.37	2974.76	3071.93	3052.57	3558.04	3855.56	3676.65	2940.5	3981	3862.8
nner Mongol	2177.63	2323.55	2824.89	3553.48	3772.63	4211.48	4962.4	5463.18	6117.93	4746.4	6210.3	6445.8
Liaoning	2860.98	3102.13	3560.21	4378.14	4680.85	4658	5254.96	5809.39	5803.9	4554.8	6092.5	6901.6
Jilin	2356	2457.21	2842.68	3307.14	3637.32	3767.85	4252.85	4635.27	4658.13	3531.6	4640.6	4975.7

Heilongjiang	2071.62	2215.68	2633.18	3128.1	3397.41	3784.72	4348.45	4687.23	5069.89	3537.9	4749.7	5019.3
Shanghai	4940.06	5248.95	6125.45	7108.62	7344.83	7776.98	8905.95	9655.6	9822.88	9011.6	9690.7	10014.8
Jiangsu	3205.79	3462.66	3928.71	4544.64	4773.67	5243.14	6060.91	6658.37	7074.11	5591.7	7003.8	7389.2
Zhejiang	4140.34	4393.4	4892.58	5522.56	5604.72	6118.46	7066.22	7552.02	8008.16	6569.2	8092	8467.3
Anhui	2781.5	3091.28	3384.38	3905.05	4051.4	4369.63	5246.76	5814.92	6370.23	4003.1	5802.1	6381.7
Fujian	3595.2	3854.26	4296.22	5078.85	5336.36	5790.72	6534.94	7317.42	7424.67	6081.9	7759.1	8299.6
Jiangxi	2495.09	2636.93	3192.61	3633.05	3881.56	4195.38	4675.16	5071.61	5221.1	3785.8	5407.8	5667.5
Shandong	2512.73	2711.65	3180.64	3699.42	3954.34	4205.88	4827.61	5201.32	5625.94	3932.3	5527.4	5929.4
Henan	2067.51	2215.32	2707.44	3079.82	3272.75	3575.75	4212.76	4607.47	4913.87	3202.4	4818.7	5067.7
Hubei	2625.41	2868.39	3455.98	3996.27	4160.51	4429.3	5363.68	5837.93	6259.22	4139.7	5828.6	6294.3
Hunan	2689.39	2850.94	3243.88	3970.42	4174.55	4322.09	4943.89	5441.63	5583.99	4240.5	6075.5	6407.7
Guangdong	4265.19	4503.86	5056.68	5866.91	6225.22	6746.62	7471.88	8258.44	8856.91	6589.8	8533.4	9421.6
Guangxi	2906.73	2857.4	3398.09	4082.99	4129.55	4372.75	5074.49	5552.56	5841.16	3680.1	5610.2	5937.2
Hainan	2819.96	3097.71	3546.67	4226.9	4507.81	4895.96	5673.65	6556.1	6979.22	4915	7051.8	7419.7
Chongqing	3135.65	3415.92	3674.28	4418.34	4576.23	5012.56	5847.9	6870.23	7245.12	4971.9	6627.6	6883.9
Sichuan	2709.69	2838.22	3580.14	4255.48	4391.73	4779.6	5571.69	6073.86	6471.84	4548.2	6783.1	7118.4
Guizhou	2458.3	2649.02	3122.46	3597.94	3755.61	4013.67	4565.85	4992.85	4915.02	3151.9	5282.7	6010.3
Yunnan	2997.06	3102.46	3562.33	4272.29	4460.58	4593.49	4802.26	5468.17	5741.01	3211.5	5346.4	5528.2
Shaanxi	2401.52	2588.91	3063.69	3586.13	3988.57	4381.4	5040.47	5550.71	6075.58	3405.1	5146.4	5422
Gansu	2352.82	2408.37	2824.42	3183.79	3359.3	3702.18	4182.47	4602.33	5162.87	3218.2	5345.9	5777.3
Qinghai	2267.36	2366.42	2803.45	3315.94	3548.85	3784.81	4260.27	4667.34	4777.1	3854.4	5502.6	5975.7
Ningxia	2228.63	2444.98	2760.74	3352.83	3432.23	3768.09	4483.44	4768.91	4895.2	3555.6	4883.4	4889.2
Xinjiang	2257.44	2386.97	2760.69	3235.77	3386.33	3694.81	4537.46	5238.89	5323.5	3855	5954.9	6179.4

Per capita food consumption expenditure of rural residents(Unit: yuan) (Data from China Statistical Yearbook)												
Region	2005	2006	2007	2008	2009	2010	2011	2012	2013	2014	2015	2016
Beijing	1735.95	1878.95	2132.51	2470.72	2808.92	2994.66	3593.48	3944.76	4695.9	4048	4372.1	4667.1
Tianjin	1171.39	1212.62	1367.75	1568.95	1848.11	2060.83	2375.97	3019.86	3539.7	4314.4	4346.3	4980.9
Hebei	888.37	915.5	1025.72	1192.93	1195.65	1351.41	1579.65	1817	1963.3	2421.2	2578.1	2745.4
Shanxi	830.48	867.65	1033.68	1206.69	1224.6	1372.49	1729.91	1859.98	1920.7	2054.3	2150.2	2272.4
nner Mongoli	1054.26	1082.07	1280.05	1483.61	1578.57	1675.04	2067.03	2379.76	2583.5	3039	3123	3362.9
Liaoning	1127.23	1162.53	1334.18	1549	1563.33	1714.15	2116.3	2299.99	2518.9	2210.9	2498.8	2678.6
Jilin	1003.22	1082.28	1240.93	1362.44	1371.12	1523.32	1872.09	2268.76	2438.5	2411.2	2550.8	2721.9
Heilongjiang	923.59	923.7	1077.34	1267.68	1331.07	1483.95	2072.44	2164.94	2397.7	2210.2	2306.7	2609.1
Shanghai	2683.89	3023.53	3259.48	3731.27	3639.14	3806.82	4517.16	4847.59	5334.6	5332.7	5660	5731.9
Jiangsu	1569.27	1728.99	1968.88	2202.58	2275.28	2491.51	2839.93	3049.11	3283.2	3711.9	4078.3	4254.7
Zhejiang	2061.44	2218.88	2430.6	2779.1	2812.39	3055.59	3714.82	3947.31	4190.9	4618.5	5008.4	5520.2
Anhui	999.79	1045.19	1192.57	1454.18	1494.19	1632.96	2055.23	2180.8	2269.7	2842.3	3212	3523
Fujian	1517.60	1621.92	1870.32	2162.3	2304.14	2537.15	3032.17	3403.46	3600.8	4222.5	4493.8	4818.3
Jiangxi	1220.53	1312.28	1492.02	1633.12	1609.2	1812.66	2106.27	2232.83	2389.1	2755.1	3071.8	3221.7
Shandong	1087.65	1191.32	1369.2	1551.77	1618.66	1804.45	2107.07	2321.46	2553.7	2464.5	2661.6	2832.8
Henan	858.97	911.48	1017.43	1165.81	1220.36	1371.17	1559.74	1701.75	1938.5	2153.8	2301.3	2447.3
Hubei	1192.26	1278.88	1479.04	1711.34	1668.35	1763.05	1954.62	2154.01	2308.5	2724.1	2952.7	3295.3
Hunan	1433.01	1463.33	1675.16	1947.52	1967.54	2087.85	2343.06	2574.81	2537	3095.2	3188.9	3370.7
Guangdong	1789.42	1887.17	2087.58	2388.91	2425.55	2630.05	3301.14	3658.66	3736.6	3968.9	4511.3	5010.5
Guangxi	1186.71	1196.07	1378.78	1594.67	1572.82	1675.41	1844.94	2085.63	2084.7	2462.9	2680.6	2880.4
Hainan	1134.75	1191.09	1430.31	1537.55	1639.34	1724.47	2137.9	2410.07	2625	3037.2	3506.3	3854.3
Chongqing	1130.35	1150.98	1376	1537.59	1542.12	1750.01	2108.61	2216.15	2539	3229	3571.1	3850.7
Sichuan	1244.36	1216.19	1435.52	1627.58	1740.59	1881.18	2161.65	2514.16	2665	3299.3	3618.4	3886.6
Guizhou	819.87	838.42	998.39	1119.64	1093.94	1319.43	1646.53	1740.58	2036.2	2223.5	2270.2	2316.5
Yunnan	975.72	1071.13	1226.69	1483.16	1410	1604.5	1883.95	2080.61	2097.6	2145.9	2486.7	2586
Shaanxi	812.93	850.2	941.81	1115.66	1175.29	1299.22	1344.99	1520.1	1821.3	2112.2	2199.5	2307
Gansu	858.89	865.99	944.14	1132.53	1142.05	1315.25	1548.19	1648.6	1798.5	2145.7	2244.1	2342.6
Qinghai	893.32	938.5	1069.04	1220.02	1164.07	1442.88	1716.39	1858.62	1872	2626	2564.2	2715.4
Ningxia	922.54	929.15	1019.35	1288.47	1395.42	1541.77	1762.53	1891.37	2021.8	2296	2452.7	2419.1
Xinjiang	803.82	810.74	939.03	1146.69	1225.93	1394.38	1589.46	1891.1	2072	2540.2	2622.5	2624.2

The proportion of the added value of tertiary industry to regional GDP(%) (Data from China Statistical Yearbook)												
Region	2005	2006	2007	2008	2009	2010	2011	2012	2013	2014	2015	2016
Beijing	69.1	70.9	72.1	73.2	75.5	75.1	76.1	76.5	76.9	77.9	79.7	80.2
Tianjin	41.5	40.2	40.5	37.9	45.3	46	46.2	47	48.1	49.6	52.2	56.4
Hebei	33.3	33.8	34	33.2	35.2	34.9	34.6	35.3	35.5	37.3	40.2	41.5
Shanxi	37.4	36.4	35.3	34.2	39.2	37.1	35.2	38.7	40	44.5	53.2	55.5
nner Mongoli	39.4	37.8	35.7	33.3	38	36.1	34.9	35.5	36.5	39.5	40.5	43.8
Liaoning	39.6	38.3	36.6	34.5	38.7	37.1	36.7	38.1	38.7	41.8	46.2	51.5
Jilin	39.1	39.5	38.3	38	37.9	35.9	34.8	34.8	35.5	36.2	38.8	42.5
Heilongjiang	33.7	33.7	34.7	34.4	39.3	37.2	36.2	40.5	41.4	45.8	50.7	54
Shanghai	50.5	50.6	52.6	53.7	59.4	57.3	58	60.4	62.2	64.8	67.8	69.8
Jiangsu	35.4	36.3	37.4	38.1	39.6	41.4	42.4	43.5	44.7	47	48.6	50
Zhejiang	40	40.1	40.7	41	43.1	43.5	43.9	45.2	46.1	47.8	49.8	51
Anhui	40.7	40.2	39	37.4	36.4	33.9	32.5	32.7	33	35.4	39.1	41
Fujian	38.5	39.1	40	39.3	41.3	39.7	39.2	39.3	39.1	39.6	41.6	42.9
Jiangxi	34.8	33.5	31.9	30.9	34.4	33	33.5	34.6	35.1	36.8	39.1	42
Shandong	32	32.6	33.4	33.4	34.7	36.6	38.3	40	41.2	43.5	45.3	46.7
Henan	30	29.8	30.1	28.6	29.3	28.6	29.7	30.9	32	37.1	40.2	41.8
Hubei	40.3	40.6	42.1	40.5	39.6	37.9	36.9	36.9	38.1	41.5	43.1	43.9
Hunan	40.5	40.8	39.8	37.8	41.4	39.7	38.3	39	40.3	42.2	44.1	46.4
Guangdong	42.9	42.7	43.3	42.9	45.7	45	45.3	46.5	47.8	49	50.6	52
Guangxi	40.5	39.7	38.4	37.4	37.6	35.4	34.1	35.4	36	37.9	38.8	39.6
Hainan	41.8	39.9	40.7	40.2	45.3	46.2	45.5	46.9	48.3	51.9	53.3	54.3
Chongqing	43.9	44.8	42.4	41	37.9	36.4	36.2	39.4	41.4	46.8	47.7	48.1

Sichuan	38.4	37.8	36.5	34.8	36.7	35.1	33.4	34.5	35.2	38.7	43.7	47.2
Guizhou	39.6	39.8	41.8	41.3	48.2	47.3	48.8	47.9	46.6	44.6	44.9	44.7
Yunnan	39.5	38.5	39.1	39.1	40.8	40	41.6	41.1	41.8	43.3	45.1	46.7
Shaanxi	37.8	35.3	34.9	32.9	38.5	36.4	34.8	34.7	34.9	37	40.7	42.3
Gansu	40.7	39.5	38.4	39.1	40.2	37.3	39.1	40.2	41	44	49.2	51.4
Qinghai	39.3	37.5	36	34	36.9	34.9	32.3	33	32.8	37	41.4	42.8
Ningxia	41.7	39.6	38.2	36.2	41.7	41.6	41	42	42	43.4	44.5	45.4
Xinjiang	35.7	34.7	35.4	33.9	37.1	32.5	34	36	37.4	40.8	44.7	45.1

Total industrial sulfur dioxide (SO2) emissions(Unit:ton) (Data from China Statistical Yearbook)												
Region	2005	2006	2007	2008	2009	2010	2011	2012	2013	2014	2015	2016
Beijing	105000	94000	82909.149	57783	59921.864	56844	61299	59330	52041	40347	22070	10257
Tianjin	241000	232000	224775	209844	172979.81	217620	221897	215481	207793	195395	154605	54539
Hebei	1281000	1326000	1294416	1158712	1042679.3	994177	1317099	1238737	1173147	1047351	829414	518954
Shanxi	1200000	1177000	1118474.4	1058363	1009585.7	1147115	1294321	1194634	1140835	1077990	900765	385721
nner Mongoli	1296000	1384000	1283250.5	1258581	1204018.4	1192984	1250180	1241475	1236409	1167133	1061017	430700
Liaoning	961000	1037000	1067185.8	1000779	918837.47	859410	1048914	979025	947330	926035	869328	401584.83
Jilin	308000	336000	336566.41	313202	300200.24	300636	363443	352337	330956	319643	302082	129713.43
Heilongjiang	431000	440000	440418.72	441284	418905.48	417051	415209	397284	352732	317480	280967	165200
Shanghai	375000	374000	364416.19	298000	239347.51	221476	210092	193405	172867	155360	104852	67376
Jiangsu	1312000	1241000	1160532.8	1073577	1011823.5	1002445	1025013	959210	909478	870175	794656	525100
Zhejiang	831000	829000	774638.56	715909	677002.51	653889	647048	610882	579104	560083	523973	245000
Anhui	515000	519000	516732.04	502633	486863.97	483912	487228	469776	450223	440642	420033	232362.02
Fujian	439000	446000	426878.8	409305	399192.35	391231	370269	352389	342040	337632	317063	189300
Jiangxi	555000	570000	553413.33	511173	490199.98	470954	568058	551502	543497	517408	515661	262700
Shandong	1715000	1687000	1582668.9	1465501	1366150.5	1382874	1628647	1543766	1445348	1358883	1220937	865459
Henan	1471000	1464000	1410203.4	1280622	1175968.5	1162874	1229200	1129858	1102662	1031667	915002	284700
Hubei	626000	654000	603436.69	562290	527430.77	516044	594980	548591	524005	506192	470683	185500
Hunan	755000	766000	739430.1	674803	649425.55	627383	636181	593342	588696	559504	515935	285682.05
Guangdong	1274000	1247000	1176162.2	1096928	1012579.1	989088	825952	771467	731995	699102	648998	330000
Guangxi	975000	944000	926247.04	870264	835295.78	847970	488744	471621	437996	431075	385507	130000
Hainan	22000	23000	24943.698	21204	21433.199	28167	31058	33036	31652	31855	31683	23734.218
Chongqing	683000	712000	683060.13	627238	586116.62	572747	531340	509788	494415	474805	426800	174048
Sichuan	1141000	1121000	1023118.7	968914	946424.13	937632	828929	793965	746363	725729	622441	578442.16
Guizhou	659000	1040000	920585.97	741315	623743.19	637779	903132	837101	778581	702427	598896	411800
Yunnan	429000	456000	445391.2	419931	417820.78	439554	642523	622599	612954	582558	523765	461300
Shaanxi	800000	846000	845578.82	806562	741872.63	706960	831222	747018	707146	671642	599321	190806
Gansu	517000	463000	435764.99	412370	400925.71	452464	527689	479938	472798	476964	466984	173800
Qinghai	115000	121000	125258.77	126081	127275.26	133149	134315	129094	130818	118046	116365	103493.8
Ningxia	302000	350000	340021.67	319180	278346.07	280380	387865	384378	368201	340969	303795	166000
Xinjiang	348000	429000	472537.79	510191	515398.05	518438	669050	704710	738566	718070	622123	242400

Emission of industrial smoke (powder) layer(Unit:ton)(Data from China Statistical Yearbook, China Environmental Statistical Yearbook,, provincial and municipal												
Region	2005	2006	2007	2008	2009	2010	2011	2012	2013	2014	2015	2016
Beijing	51000	45000	39840.112	35234	36397.718	37816	29405	30844	27182	22710	12987	7874
Tianjin	96000	77000	72149.515	65904	66632.899	61801	65333	59036	62766	112129	73795	57280
Hebei	1273000	1199000	996280.78	903812	756807.07	643548	1223502	1055732	1187198	1450723	1111046	883517
Shanxi	1605000	1488000	1313839.9	996778	866017.31	797383	1008410	948115	897937	1145107	1072962	634351.18
nner Mongoli	1060000	758000	704445.06	637943	484656.19	636287	604534	667689	684054	818754	656736	393900
Liaoning	970000	876000	899837.94	736953	628371.01	565289	591152	626344	572774	957920	836722	508980.07
Jilin	464000	456000	398532.2	324299	343036.61	262672	361279	195510	250928	368032	338231	144424.54
Heilongjiang	578000	568000	550523.12	490368	420350.53	353933	419810	450097	520144	534705	373165	166900
Shanghai	61000	57000	48759.493	48615	44770.312	51498	66446	63732	67174	131433	111370	72800
Jiangsu	781000	704000	609942.13	511538	465181.1	450216	486389	395978	455568	720479	612212	429700
Zhejiang	430000	415000	375122.32	336238	348053.27	304595	302752	233228	296586	358776	311123	163000
Anhui	715000	662000	561630.85	563909	514370.59	471151	410590	351639	351757	585283	477963	264606.61
Fujian	311000	285000	268416.72	245995	227334.48	240042	209852	233989	240583	349196	321763	237900
Jiangxi	580000	560000	509183.95	457811	402601.38	362583	359085	321830	324694	428759	446013	301300
Shandong	858000	741000	645398.23	587927	522575.75	480618	612740	525896	542371	1024473	902953	698570
Henan	1561000	1289000	1052031.9	818937	769660.89	700653	575655	492771	547210	715479	666348	307300
Hubei	604000	595000	485791.33	408083	362442.75	291608	307356	281577	294788	438294	378130	213368.68
Hunan	1222000	1150000	1031426.5	861641	850706.01	629336	355203	298832	316770	452295	412424	209444.19
Guangdong	592000	550000	502414.16	483121	353348.47	357630	263918	267498	296664	395328	299572	234000
Guangxi	1094000	908000	731729.56	649302	713298.89	567863	260006	268524	260028	376019	329394	210000
Hainan	21000	20000	20027.828	16975	16593.849	13100	11054	10660	14029	18854	16105	10174.91
Chongqing	344000	332000	298200.68	254279	216423.11	185733	171224	166142	179842	214774	196416	83800
Sichuan	1018000	803000	524877.96	359300	309282.69	401060	357577	267820	268866	398729	375566	307019.72
Guizhou	396000	338000	336809.95	274615	216526.15	199539	257164	256539	262072	343039	230615	146100
Yunnan	326000	308000	290273.39	273468	225378.58	180915	352817	359517	354770	329955	261367	259040.14
Shaanxi	632000	576000	550194.45	372339	298765.44	300469	396997	385522	468507	537753	454899	182045
Gansu	290000	275000	188984.37	173764	175285.78	190754	187755	156646	174556	260903	207803	116200
Qinghai	150000	141000	125833.27	130784	123166.12	149416	117630	133698	149239	175875	179821	163809.83
Ningxia	192000	185000	170673.97	137173	115728.47	197082	198220	180716	212914	211746	185339	155000
Xinjiang	323000	363000	393473.02	397527	404440.84	433034	440532	606003	663257	675956	462546	287000

Investment completed in industrial pollution control(Unit: 10000 yuan) (Data from China Statistical Yearbook, China Environmental Statistical Yearbook)												
Region	2005	2006	2007	2008	2009	2010	2011	2012	2013	2014	2015	2016
Beijing	108880.2	101397.3	81207	78474.8	34421.3	19339.5	10946	32840	42768	75695	99958	98770
Tianjin	186315.5	149534.2	150527	168270.4	180054	164683.5	152848	125559	148366	220923	240072	103597
Hebei	251519	190689.4	215485	205674.9	132272	108587.8	243399	236290	511769	889518	541596	248465

Shanxi	197885.1	367602.6	457241	529369.8	386710.9	279573.8	279450	323269	555609	311477	278738	300742
nner Mongoli	25678.1	177234.8	167487	219189.2	178258.1	132399.5	310164	189715	626746	775439	438935	406191
Liaoning	369498.8	520470.3	237002	201645	196562.2	147707.9	116032	119447	276908	382184	189950	193853
Jilin	51531.2	39810.3	80308	93864.7	79254.5	63365.5	65624	57269	93731	163707	121203	98402
Heilongjiang	45615.2	58189.4	102110	95079.3	99317.8	49494.4	100891	39287	206988	177572	193396	173809
Shanghai	87516.8	59269	164318	103901.1	68356.9	94106.5	63602	115915	52077	177859	211726	519488
Jiangsu	389480.7	280053.4	537032	397125.5	270554	185994.5	310062	390144	593776	485096	621741	747786
Zhejiang	199470.4	250363.1	213773	148006.5	193573.8	119568.2	178373	283023	576645	675944	586017	601869
Anhui	45443.1	54555.4	113853	115341.1	108282.1	58895.4	92793	127350	413195	176220	179450	415486
Fujian	345431.2	196034.8	138007	155762.8	128691.8	153295.5	142599	237635	383964	423817	446910	226267
Jiangxi	72309.6	68578.7	82688	50665.3	39539.5	63775.4	66235	39478	155192	123466	147833	104485
Shandong	605063.4	596643.1	673420	844159.4	515831.6	456759.3	624466	670633	843493	1416464	945934	1264063
Henan	206815.1	247334.6	338132	246110.4	154242	125120.3	213728	148347	439720	554592	330143	651538
Hubei	148096.5	148872.8	188634	161453.3	281332.3	277416.4	92873	148964	251745	262884	157976	369051
Hunan	141238.9	173309	133641	143905.1	133806.3	137948.6	97039	179561	233655	173424	261425	127037
Guangdong	370383.7	313708.3	462758	403275.9	227464.3	310584	166420	280996	324634	378641	347103	264812
Guangxi	103730.4	86604.2	181940	149751.4	117118.4	92845.1	86230	85644	183218	178909	247152	130433
Hainan	3788.6	21389.2	3889	3774.4	3562.5	4353.5	27534	48279	35094	56152	13161	16138
Chongqing	39121.3	36742.1	100070	97396.2	70746.6	77501.9	49384	38226	78880	50284	59885	37141
Sichuan	200385.6	203008.4	201033	193808.2	96190.6	71626.5	166537	110608	188392	232452	118259	116049
Guizhou	59258.3	100771	45646	102029.3	89474.6	68079.5	131970	124663	195562	184765	107033	56904
Yunnan	67508.1	94089.2	86423	102677.2	94880.1	106271.5	137331	197259	238930	244003	215878	127174
Shaanxi	126946.3	73746.5	97045	106582.2	205999	336535	237248	271266	417562	334478	279915	194913
Gansu	66609.6	136467.9	149087	118435.8	123301.9	146483.2	105338	210984	182144	176244	40526	109742
Qinghai	4670	7773.2	7913	11165.3	29438.8	9747.2	27858	21880	30456	74508	49343	96249
Ningxia	17709.8	39885.5	46272	90631	43471.8	40896	38735	69160	165486	272967	104318	242101
Xinjiang	44008.2	45228.5	66748	88878.3	143497.2	66812.8	106276	79106	220054	316542	158263	146370

Electricity consumption(Unit: 100 million kWh) (Data from China Energy Statistical Yearbook, provincial and municipal statistical yearbooks)												
Region	2005	2006	2007	2008	2009	2010	2011	2012	2013	2014	2015	2016
Beijing	570.5364	611.5719	667.0089	689.7189	739.1465	809.9	821.71	874.28	913.11	937.05	952.72	1020.27
Tianjin	384.8446	433.6501	494.9107	515.8839	550.1556	645.74	695.15	722.48	774.49	794.36	800.6	807.93
Hebei	1501.924	1734.832	2013.6743	2095.0199	2343.8467	2691.52	2984.9	3077.73	3251.19	3314.11	3175.66	3264.52
Shanxi	946.3268	1097.6771	1348.8115	1314.3337	1267.5376	1460	1650.41	1765.79	1832.35	1822.63	1737.21	1797.18
nner Mongoli	667.7197	884.9083	1160.2124	1220.5748	1287.9256	1536.83	1864.07	2016.76	2181.9	2416.74	2542.87	2605.03
Liaoning	1110.5565	1228.2742	1359.5146	1411.9992	1488.1718	1715.26	1861.53	1899.88	2008.46	2038.73	1984.89	2037.4
Jilin	378.2271	412.4577	462.6384	496.4888	515.2545	576.98	630.15	637	653.85	667.81	651.96	667.63
Heilongjiang	555.8518	597.0466	628.9364	669.9031	688.668	747.84	801.87	827.91	845.2	859.42	868.97	896.62
Shanghai	921.9671	990.145	1072.3793	1138.2162	1153.3794	1295.87	1339.62	1353.45	1410.6	1369.03	1405.55	1486.02
Jiangsu	2193.4526	2569.7523	2952.0196	3118.3216	3313.9857	3864.37	4281.62	4580.9	4956.62	5012.54	5114.7	5458.95
Zhejiang	1642.312	1909.2315	2189.3667	2322.8718	2471.4378	2820.93	3116.91	3210.55	3453.05	3506.39	3553.9	3873.19
Anhui	582.1581	662.1832	769.1045	858.8769	952.3056	1077.91	1221.19	1361.1	1528.07	1585.18	1639.79	1794.98
Fujian	756.5852	866.8444	1000.3344	1073.5477	1134.9185	1315.09	1515.86	1579.5	1700.73	1855.79	1851.86	1968.58
Jiangxi	391.9846	446.1968	511.0887	545.8822	609.2236	700.51	835.1	867.67	947.11	1018.52	1087.26	1182.5
Shandong	1911.6124	2272.072	2596.051	2726.9658	2941.0672	3298.46	3635.26	3794.55	4083.12	4223.49	5117.05	5390.75
Henan	1352.7358	1523.5026	1807.9984	1970.7676	2081.3756	2353.96	2659.14	2747.75	2899.18	2919.57	2879.62	2989.15
Hubei	788.906	876.757	989.2331	1058.5302	1135.1268	1330.44	1450.76	1507.85	1629.75	1656.54	1665.16	1763.11
Hunan	674.4261	768.7729	890.5834	904.9541	1010.5702	1171.91	1293.44	1345.22	1423.09	1430.88	1447.63	1495.65
Guangdong	2673.5565	3004.0334	3394.0491	3504.8232	3609.6424	4060.13	4399.02	4619.41	4830.13	5235.23	5310.69	5610.13
Guangxi	510.148	579.4618	681.136	753.3871	856.3511	993.24	1112.21	1153.42	1237.74	1307.99	1334.32	1359.65
Hainan	81.6081	97.6759	113.254	121.7218	133.7675	159.02	185.28	208.08	232.02	251.88	272.36	287.31
Chongqing	347.6778	405.1961	449.216	484.4075	533.7976	626.44	717.03	723.03	813.26	867.24	875.37	924.89
Sichuan	942.5909	1059.4386	1177.5094	1210.1335	1324.61	1549.03	1751.44	1830.7	1948.95	2014.79	1992.4	2101.02
Guizhou	486.9747	581.978	669.0998	679.1764	750.3007	835.38	944.13	1046.72	1126.27	1173.74	1174.21	1241.78
Yunnan	557.25	645.6136	745.5182	829.4436	891.1902	1004.07	1204.07	1315.86	1459.81	1529.38	1438.61	1410.52
Shaanxi	516.4317	580.7287	653.6864	708.0274	740.1138	859.22	982.47	1066.75	1152.22	1226.01	1221.73	1357.06
Gansu	489.4817	536.3349	614.7392	677.7597	705.5127	804.43	923.45	994.56	1073.25	1095.48	1098.72	1065.15
Qinghai	206.5571	244.4149	285.4372	313.2254	337.2368	465.18	560.68	602.22	676.29	723.21	658	637.51
Ningxia	302.8839	377.85	439.7756	439.6154	462.9585	546.77	724.54	741.79	811.18	848.75	878.33	886.91
Xinjiang	310.1361	356.1989	413.3222	479.3708	547.8766	661.96	839.1	1090.8	1539.75	1900.24	2160.34	2316.46

Total imports(Unit: USD 10000)(According to the location of the operating unit) (Data from China Statistical Yearbook)												
Region	2005	2006	2007	2008	2009	2010	2011	2012	2013	2014	2015	2016
Beijing	9464052	12008265	14407337	21419329	16635373	24628534	33055883	34847523	36589825	35318018	26477375	23032612
Tianjin	2589592	3097116	3337568.1	3829784.6	3393851.5	4461522.4	5889423	6732171	7949685	8129542	6311988	5837725
Hebei	514605	569688	852300.9	1441641.2	1393835.1	1950392.7	2503099	2096485	2395096	2416716	1858099	1609984
Shanxi	201716	248747	504699.1	514194	573157.7	787341	931793	802707	779541	729194	626055	672922
nner Mongoli	310263	382032	479149.1	532663.3	445859	539531.5	724393	728882	790201	816277	708114	724431
Liaoning	1757494	2007083	2415025.6	3036435.2	2951944.9	3761343.7	4499350	4613094	4995618	5525307	4523614	4349413
Jilin	406157	491739	644094.5	856049.6	861747.3	1236933.3	1706322	1858033	1909283	2060320	1426364	1425088
Heilongjiang	349658	442060	503947.1	632434.8	614738.6	923462.9	2084969	2315512	2264737	2156569	1297662	1150365
Shanghai	9561922	11393493	13900777	15291017	13591758	18823667	22787477	22985679	23708819	25626598	25332751	25041606
Jiangsu	10495605	12356876	14586201	15424252	13954051	19526027	22699084	21943797	22200052	22172058	20691567	19024332
Zhejiang	3058721	3825105	4858339.5	5683749.9	5471790.9	7306987.3	9302827	8788421	8704246	8171272	7045171	6871216
Anhui	393088	540739	711855.2	881973.7	679123.9	1186048.4	1422661	1253604	1726766	1769193	1557436	1596618
Fujian	1956932	2139789	2450980.8	2782922.5	2633048.3	3729016.1	5068465	5810536	6284648	6395555	5616582	5314821
Jiangxi	162527	244185	400395.4	589127.5	541029.5	820311.4	959275	830104	857998	1070550	928287	1023001
Shandong	3061298	3661548	4736433.8	6521272.7	5956266.3	8493068.8	11017351	11683512	13234141	13222074	9668212	9725976
Henan	263739	316016	441021.1	676044	613103.9	730214.2	1338267	2206236	2396977	2558918	3071920	2840759
Hubei	462607	550157	669601.5	899676.1	727221.9	1149031.5	1405233	1256525	1354387	1639720	1634076	1334943
Hunan	225305	226043	317045.6	413431	465743.2	670040.1	903997	934653	1035411	1088860	1016471	855093

Guangdong	18980614	22525267	26486987	27930433	25213916	33170496	38154076	40996969	45521759	43049746	37932360	35669602
Guangxi	230487	307459	414984	588872.4	587935.6	813583.3	1089821	1401671	1413424	1622143	2315657	2470102
Hainan	151979	147059	214995.3	294131.9	357300.1	632824.7	1021442	1118600	1127895	1144592	1022393	922263
Chongqing	177226	211866	293073.7	379934.5	343245	493813.7	937599	1463600	2189627	3203078	1928001	2209926
Sichuan	320036	439670	577216.5	898116.3	999920.4	1385323.9	1869688	2067453	2262560	2536384	1809566	2135863
Guizhou	54463	57928	80483.4	146543.2	94760	122662.1	190249	167933	140413	137407	227279	95683
Yunnan	210172	283334	402528.8	461250.3	353434.3	582435.3	655632	1099636	963218	1081996	787565	841205
Shaanxi	149998	173068	221209	294800.9	441724.3	589346.6	761225	614677	990240	1343469	1570975	1410966
Gansu	153928	231533	386501.3	449407.9	313004.3	576516.8	656980	532721	555879	331113	214021	277042
Qinghai	9011	11752	22616	26972.3	33491	32276.4	26199	42871	55548	58999	29251	15914
Ningxia	27915	49450	49584.1	62103.6	45954.9	78998.7	68632	57559	66553	113236	77615	76574
Xinjiang	290157	196404	221365.8	291826	301326.8	416145.8	599395	582441	529366	419155	217332	205558

Total import and export(Unit: USD 10000)(According to the location of the operating unit) (Data from China Statistical Yearbook)												
Region	2005	2006	2007	2008	2009	2010	2011	2012	2013	2014	2015	2016
Beijing	12550643	15803663	19299976	27169290	21473305	30172155	38955598	40810732	42899581	41551859	31944057	28234896
Tianjin	5327680	6446194	7144973.3	8040083.6	6383123.4	8210005	10337617	11563427	12850179	13388608	11428280	10265595
Hebei	1607035	1853088	2552341	3842053.3	2962725.2	4206037	5360084	5056306	5491157	5987736	5151375	4667538
Shanxi	554565	662710	1157948.3	1439505.7	856903.2	1257622.5	1474306	1504311	1579098	1623281	1468132	1666141
nner Mongoli	487625	596082	773588.5	891848.3	677406.6	872974.1	1193090	1125898	1199457	1455632	1273115	1164030
Liaoning	4101327	4839024	5947434.5	7243384.9	6293437.8	8071214.8	9603585	10409000	11447819	11399826	9594713	8655690
Jilin	652772	791404	1029800.1	1333212.5	1174240.7	1684518.2	2206093	2456301	2583174	2638079	1887739	1845289
Heilongjiang	956602	1285655	1729659.3	2313059.3	1622951.3	2551541.5	3852268	3759029	3887909	3890093	2101203	1653918
Shanghai	18633674	22752420	28285388	32205531	27771361	36895065	43754862	43658695	44126822	46639984	44924072	43376819
Jiangsu	22792276	28397838	34947179	39227193	33873970	46579896	53958089	54796149	55080227	56355308	54556045	50929641
Zhejiang	10738966	13914161	17684737	21113373	18773086	25353466	30937777	31240136	33578871	35503977	34678383	33657591
Anhui	911939	1224514	1593228.6	2018384.8	1567772.6	2427337.2	3130925	3928454	4551897	4917730	4784453	4441286
Fujian	5441119	6265963	7444738.1	8482106.8	7964959.2	10878329	14352243	15593796	16932090	17740784	16884593	15682619
Jiangxi	406461	619486	944854.1	1361793.1	1277878.2	2161917.7	3146881	3341383	3674663	4273082	4239960	4002841
Shandong	7673587	9521381	12247444	15840751	13905337	18915629	23588608	24554432	26653153	27692940	24060780	23435585
Henan	772492	979457	1278512.7	1747933.7	1347641.5	1783151.4	3262258	5173881	5995687	6497221	7378056	7121310
Hubei	905475	1176219	1486895.4	2070567	1725101.5	2593211	3358693	3196375	3638008	4303962	4555258	3938877
Hunan	600019	735226	968585.3	1254719.4	1014946.6	1465639	1894376	2194873	2517531	3083160	2930180	2624347
Guangdong	42796497	52719910	63418595	68496880	61109405	78489612	91346733	98402046	109158144	107658447	102249568	95529801
Guangxi	518150	666756	925899.7	1323616.8	1425472.6	1773890.6	2335597	2948446	3282750	4054885	5109055	4762743
Hainan	254234	284621	351441.1	452851.7	488163.3	864857.7	1275604	1432210	1498543	1586266	1396697	1134843
Chongqing	429284	546968	743794.4	952139.4	771252.1	1242707.4	2920764	5320358	6869216	9543158	7446685	6275364
Sichuan	790196	1102082	1437812.4	2211364.7	2416865.1	3269386.4	4772417	5914360	6457466	7020297	5118856	4930625
Guizhou	140357	161771	227030	336620.8	230421.2	314680.1	488758	663156	829010	1077133	1222142	569962
Yunnan	474344	622484	879356.7	959691.6	804759.5	1343012	1602877	2101373	2530356	2960742	2449128	1990236
Shaanxi	457687	536029	688733.9	832882.6	840539.2	1210168.1	1464727	1479903	2012806	2736449	3049850	2994722
Gansu	263027	382493	552367	609543.3	386555.4	740295.3	872858	890075	1023611	864062	795202	683298
Qinghai	41331	65172	61207.3	68882.3	58678.5	78896.1	92382	115747	140274	171789	193447	152920
Ningxia	96657	143713	158151.5	187940.4	120247.9	195998.9	228575	221671	321769	543521	373926	325249
Xinjiang	794049	910327	1371582.8	2221736	1394783.1	1713011	2281967	2517006	2756139	2767232	1966940	1763774

Foreign exchange income from international tourism(Unit: Millions of dollars) (Data from China Statistical Yearbook, New China 60 years of statistical data compilation,												
Region	2005	2006	2007	2008	2009	2010	2011	2012	2013	2014	2015	2016
Beijing	3618.91	4026.3	4579.62	4459.13	4356.68	5044.61	5416	5149	4794.68	4608	4605	5070
Tianjin	509.01	625.9	778.71	1001.39	1182.64	1419.51	1755.53	2226.41	2591.28	2992.1	3298.11	3556.87
Hebei	209.17	243.1	309.11	273.95	307.81	350.71	447.65	544.94	585.78	534.19	501.91	552.41
Shanxi	116.22	164.2	221.71	300.65	377.94	464.6	567.19	720.24	822.68	280.73	297.1	317.38
nner Mongoli	352.07	403.7	544.85	577.19	558.31	601.9	670.97	771.96	962.29	1002.96	962.49	1139.03
Liaoning	737.77	934.3	1227.86	1526.18	1856.21	2259.33	2713.14	3263.69	3477.14	1618	1636.5	1823.92
Jilin	119.52	137.2	179.31	211.44	242.94	304.92	385.28	494.77	552.37	583.9	724.14	791.21
Heilongjiang	340.43	492	642.70	869.95	638.68	762.5	917.62	835.48	604.36	563.56	395.33	458.05
Shanghai	3555.88	3903.9	4672.97	4971.72	4744.02	6340.92	5751.18	5493.23	5244.7	5601.85	5860.44	6419.2
Jiangsu	2259.74	2786.64	3469.00	3880.20	4016.01	4783.43	5652.97	6299.72	2379.89	3032.71	3527.29	3803.62
Zhejiang	1716.26	2132.7	2707.90	3024.08	3223.58	3930.2	4541.73	5151.74	5392.93	5753.48	6788.47	3127.59
Anhui	185.58	226.73	344.00	454.45	565.84	708.98	1179.18	1562.67	1660.42	1840.26	2262.87	2542.36
Fujian	1305.29	1471.1	2169.36	2393.53	2599.23	2978.24	3634.44	4225.67	4573.38	4911.8	5561.4	6625.69
Jiangxi	103.95	139.61	195.54	251.70	289.75	346.03	415	484.73	525.08	556.87	567	584.54
Shandong	780.23	1014.05	1351.85	1391.10	1765.3	2155.04	2550.76	2923.65	2731.2	2330.1	2896.48	3063.42
Henan	216.04	273.76	318.01	374.44	433.03	498.77	549.03	611.41	659.98	538.37	623.6	646.5
Hubei	276.36	320	412.64	442.55	510.2	751.16	940.18	1202.97	1218.92	1238.51	1671.9	1872.39
Hunan	390.24	503.36	642.18	617.42	672.7	906.22	1014.34	928.36	822.69	799.99	857.72	1004.57
Guangdong	6388.05	7532.79	8705.52	9174.98	10028.13	12382.61	13906.19	15610.67	16278.07	17106.36	17884.66	18577.13
Guangxi	358.93	423.12	577.08	601.66	643.34	806.15	1051.88	1278.87	1547.3	1572.07	1916.86	2164.27
Hainan	128.46	229.12	301.60	313.88	276.66	322.36	376.15	348.02	337.48	268.63	248.52	349.89
Chongqing	264.36	308.72	382.31	449.78	537.21	703.2	968.06	1168.32	1268.31	1354.44	1468.57	1686.82
Sichuan	315.95	395.23	512.43	153.88	288.56	354.09	593.83	798.15	764.76	857.68	1180.87	1581.68
Guizhou	101.41	115.16	129.18	116.97	110.44	129.58	135.07	168.94	201.43	188.8	231.33	252.71
Yunnan	528.01	658.44	859.58	1007.55	1172.21	1323.65	1608.61	1947.08	2418.18	2420.65	2875.5	3074.77
Shaanxi	446.25	510.87	612.11	660.11	771.07	1015.96	1295.05	1597.47	1676.19	1768.73	2000.22	2338.55
Gansu	58.76	62.93	70.21	16.03	12.54	14.81	17.4	22.35	20.39	10.17	14.18	19.14
Qinghai	11.02	13.25	15.90	10.15	15.42	20.45	26.59	24.32	19.42	24.74	38.76	44.16
Ningxia	2.3	2.43	2.61	3.01	4.43	5.99	6.2	5.45	12.08	18.48	20.84	40.58
Xinjiang	100.09	128	161.90	135.78	136.63	185.42	465.19	550.57	585.02	497.04	555.89	518.73

Investment rate(%)(Data from China Statistical Yearbook)

Region	2005	2006	2007	2008	2009	2010	2011	2012	2013	2014	2015	2016
Beijing	52	50.5	48.7	41	43.2	43.2	41.1	41.4	40.3	39	36.9	39.2
Tianjin	53.2	54.2	57.9	56.2	72.6	75.1	76	76.4	76.9	76.5	66.5	59.2
Hebei	45.8	47.2	49.3	51.7	53.8	54.1	56.7	57.4	57.9	59	58.2	58.1
Shanxi	53.1	54.2	55.9	57.2	66	68.9	64.5	67.9	72.8	72.5	72.6	72.5
nner Mongoli	73	72.3	73.8	68	77	77.3	76.7	84.6	93.4	77.4	78.7	69.6
Liaoning	50	54.1	57.5	65	61.9	62	62.7	62.4	62.6	61	44	43.5
Jilin	49.2	57.9	69.3	79.8	79.6	78.8	73.5	72	69.6	70.6	70.7	68.7
Heilongjiang	35.5	37.7	43	46.4	58.6	54.3	54.7	59.5	65.6	61.8	64.7	60.8
Shanghai	45.7	45.9	45.7	44	45	43.2	40.3	38	38.7	37.2	38	40.2
Jiangsu	50.9	49.3	48.1	49.4	51	51.1	51	50.4	48.4	45.8	43.6	42.8
Zhejiang	48	46.4	45.3	45.4	46.1	46.7	45.6	44.6	45.5	44.4	44	45.3
Anhui	44	45.3	46.4	47.8	48.8	49.9	50.5	51.5	57.6	52.3	51.4	51.2
Fujian	44.8	46.9	50.4	52.3	54.1	54.2	56.2	57.4	58.8	58.9	58.3	57.7
Jiangxi	48.8	50.4	50.3	50.6	54.4	51.4	51.2	50.3	49.9	50.4	49.8	50.3
Shandong	50.1	49.1	48.6	48.2	53.4	54.9	55	55.1	56.6	56.8	56.5	50.9
Henan	47.4	50.8	55.7	58.9	68.3	69.2	71.2	74.5	77.2	78	76.4	73
Hubei	45.1	47.4	46.6	48.7	51.6	52.6	54.7	55.4	56	56.1	55.8	57.5
Hunan	39.5	42.5	43.9	48.3	51.9	54.7	55.5	56.4	57.1	57.9	53.8	53
Guangdong	37.5	36.7	35.9	36.3	37.9	39.2	39.5	40.1	41.9	42.4	41.7	42.9
Guangxi	42.9	46.8	51	52.5	74.7	82.4	85.2	84.9	70.5	68.8	68.2	67.5
Hainan	47.4	47.2	45.6	48.9	55.3	57.4	59.3	70.4	73.9	74.2	62.6	63.6
Chongqing	61.6	61.9	62.3	59.8	58.5	57.7	57.5	55.6	54.6	54.4	53.7	53.8
Sichuan	45	48.1	49.4	53.1	54.4	53.6	52.6	52.3	51.4	50.6	49.3	49.1
Guizhou	51.8	51.5	51.8	52.5	53.7	56	56.6	60.8	65.7	66	67.6	69.6
Yunnan	57.3	59.6	56.2	56.2	60.9	77.2	80.3	83.2	84.9	91.1	92.6	94
Shaanxi	57.5	61.9	60.9	64.3	66.7	67.5	67.8	68.6	68.8	67.7	66	66.5
Gansu	47.4	47.9	48.9	61.7	56.6	56.9	57.2	58.4	60.2	60.7	65.5	67.7
Qinghai	68	66.7	63.4	66.9	73.8	80.5	83.8	90.8	119.9	130.4	139.6	138.6
Ningxia	78.5	74.4	73.6	79.5	96.7	92.5	83.5	89.1	91	112.8	124.3	120.8
Xinjiang	61.7	63.5	59.3	53.6	59.6	62	63	77.2	86	89.3	94.2	89.8

Urbanization rate(Unit: 10000 people) (Data from China Statistical Yearbook)												
Region	2005	2006	2007	2008	2009	2010	2011	2012	2013	2014	2015	2016
Beijing	83.62	84.33	84.5	84.9	85	85.96	86.2	86.2	86.3	86.35	86.5	86.5
Tianjin	75.11	75.73	76.31	77.23	78.01	79.55	80.5	81.55	82.01	82.27	82.64	82.93
Hebei	37.69	38.77	40.25	41.9	43.74	44.5	45.6	46.8	48.12	49.33	51.33	53.32
Shanxi	42.11	43.01	44.03	45.11	45.99	48.05	49.68	51.26	52.56	53.79	55.03	56.21
nner Mongoli	47.2	48.64	50.15	51.71	53.4	55.5	56.62	57.74	58.71	59.51	60.3	61.19
Liaoning	58.7	58.99	59.2	60.05	60.35	62.1	64.05	65.65	66.45	67.05	67.35	67.37
Jilin	52.52	52.97	53.16	53.21	53.32	53.35	53.4	53.7	54.2	54.81	55.31	55.97
Heilongjiang	53.1	53.5	53.9	55.4	55.5	55.66	56.5	56.9	57.4	58.01	58.8	59.2
Shanghai	89.09	88.7	88.7	88.6	88.6	89.3	89.3	89.3	89.6	89.6	87.6	87.9
Jiangsu	50.5	51.9	53.2	54.3	55.6	60.58	61.9	63	64.11	65.21	66.52	67.72
Zhejiang	56.02	56.5	57.2	57.6	57.9	61.62	62.3	63.2	64	64.87	65.8	67
Anhui	35.5	37.1	38.7	40.5	42.1	43.01	44.8	46.5	47.86	49.15	50.5	51.99
Fujian	49.4	50.4	51.4	53	55.1	57.1	58.1	59.6	60.77	61.8	62.6	63.6
Jiangxi	37	38.68	39.8	41.36	43.18	44.06	45.7	47.51	48.87	50.22	51.62	53.1
Shandong	45	46.1	46.75	47.6	48.32	49.7	50.95	52.43	53.75	55.01	57.01	59.02
Henan	30.65	32.47	34.34	36.03	37.7	38.5	40.57	42.43	43.8	45.2	46.85	48.5
Hubei	43.2	43.8	44.3	45.2	46	49.7	51.83	53.5	54.51	55.67	56.85	58.1
Hunan	37	38.71	40.45	42.15	43.2	43.3	45.1	46.65	47.96	49.28	50.89	52.75
Guangdong	60.68	63	63.14	63.37	63.4	66.18	66.5	67.4	67.76	68	68.71	69.2
Guangxi	33.62	34.64	36.24	38.16	39.2	40	41.8	43.53	44.81	46.01	47.06	48.08
Hainan	45.2	46.1	47.2	48	49.13	49.8	50.5	51.6	52.74	53.76	55.12	56.78
Chongqing	45.2	46.7	48.3	49.99	51.59	53.02	55.02	56.98	58.34	59.6	60.94	62.6
Sichuan	33	34.3	35.6	37.4	38.7	40.18	41.83	43.53	44.9	46.3	47.69	49.21
Guizhou	26.87	27.46	28.24	29.11	29.89	33.81	34.96	36.41	37.83	40.01	42.01	44.15
Yunnan	29.5	30.5	31.6	33	34	34.7	36.8	39.31	40.48	41.73	43.33	45.03
Shaanxi	37.23	39.12	40.62	42.1	43.5	45.76	47.3	50.02	51.31	52.57	53.92	55.34
Gansu	30.02	31.09	32.25	33.56	34.89	36.12	37.15	38.75	40.13	41.68	43.19	44.69
Qinghai	39.25	39.26	40.07	40.86	41.9	44.72	46.22	47.44	48.51	49.78	50.3	51.63
Ningxia	42.28	43	44.02	44.98	46.1	47.9	49.82	50.67	52.01	53.61	55.23	56.29
Xinjiang	37.15	37.94	39.15	39.64	39.85	42.79	43.54	44	44.47	46.07	47.23	48.35

Urban permanent population(Unit: 10000 people) (Data from China Statistical Yearbook)												
Region	2005	2006	2007	2008	2009	2010	2011	2012	2013	2014	2015	2016
Beijing	1286.1	1350.2	1416.2	1503.6	1581.1	1686.4	1740.7	1783.7	1825.1	1859	1877.7	1879.6
Tianjin	783.3973	814.0975	850.8565	908.2248	957.9628	1033.3545	1090.775	1152.3015	1207.1872	1248.0359	1278.4408	1295.3666
Hebei	2582.1419	2674.3546	2794.5575	2928.391	3076.6716	3201.33	3301.896	3410.784	3528.6396	3642.5272	3811.2525	3983.004
Shanxi	1412.81	1451.39	1493.75	1538.58	1576.09	1717.43	1785.31	1851.08	1907.92	1962.32	2016.37	2069.63
nner Mongoli	1134.3	1174.7	1218	1264.1	1312.7	1372.9	1405.2	1437.6	1466.3	1490.6	1514.2	1542.1
Liaoning	2477.727	2519.4629	2544.416	2591.1575	2619.7935	2716.875	2807.3115	2881.3785	2917.155	2944.1655	2951.277	2949.4586
Jilin	1206.3	1208.8	1215.9	1224.8	1226.8	1242.1	1309.1	1266.7	1258.4	1247.8	1289	1303.7
Heilongjiang	2028.4	2045.3	2061.1	2119	2123.4	2133.7	2166.2	2181.5	2201.3	2223.5	2241.5	2249.1
Shanghai	1683.801	1742.068	1830.768	1896.926	1958.06	2056.579	2095.871	2125.34	2163.84	2173.696	2115.54	2127.18
Jiangsu	3832.06	3973.29	4108.7	4215.17	4342.51	4767.63	4889.36	4990.09	5090.01	5190.76	5305.83	5416.65
Zhejiang	2795.9582	2865.68	2948.66	3002.112	3054.804	3356.4414	3403.449	3461.464	3518.72	3573.0396	3644.662	3745.3
Anhui	2172.6	2266.81	2367.666	2484.675	2581.151	2562.1057	2673.664	2784.42	2885.958	2989.7945	3102.72	3221.3004
Fujian	1758	1807	1856	1929	2019	2109	2161	2234	2293	2352	2403	2464
Jiangxi	1599.4715	1678.375	1738.6282	1819.8829	1913.8059	1966.0669	2051.2156	2139.8181	2209.9731	2281.0731	2356.779	2438.4924

Shandong	4161.6	4291.449	4379.0725	4482.492	4575.904	4765.236	4910.0515	5077.8455	5231.4875	5384.9289	5613.7747	5870.7194
Henan	2994	3189	3389	3573	3758	4052	4255	4473	4643	4819	5023	5232
Hubei	2466.7	2493.5	2524.7	2581.4	2631.2	2844.51	2984.32	3091.77	3161.03	3237.8	3326.58	3419.19
Hunan	2340.62	2454.9882	2570.5975	2689.17	2767.392	2844.81	2974.796	3097.0935	3209.0036	3319.9936	3451.8687	3598.605
Guangdong	5578.9192	5948.46	6099.324	6269.1941	6422.42	6909.8538	6985.825	7140.356	7212.3744	7292.32	7454.3479	7611.308
Guangxi	1567	1635	1728	1838	1904	1849	1942	2038	2115	2187	2257	2326
Hainan	374.256	385.396	398.84	409.92	424.4832	432.762	442.885	457.692	472.023	485.4528	502.1432	520.6726
Chongqing	1265.95	1311.29	1361.35	1419.09	1474.92	1529.55	1605.96	1678.11	1732.76	1783.01	1838.41	1908.45
Sichuan	2709.96	2801.967	2893.212	3043.612	3167.595	3232.481	3367.315	3515.4828	3640.043	3768.82	3912.4876	4065.7302
Guizhou	1002.251	1013.274	1025.6768	1046.7956	1057.2093	1176.2499	1212.7624	1268.5244	1324.8066	1403.5508	1482.953	1569.5325
Yunnan	1312.9	1367.3	1426.4	1499.2	1554.1	1601.8	1704.2	1831.5	1897.1	1967.1	2054.6	2148.2
Shaanxi	1374	1447	1506	1565	1621	1707	1770	1877	1931	1985	2045	2110
Gansu	764.04	791.8	804.97	820.11	834.18	924.66	952.6	998.8	1036.23	1079.84	1122.75	1166.39
Qinghai	213.21	215.02	221.02	226.49	233.51	251.98	262.62	271.92	280.3	290.4	295.98	306.4
Ningxia	252.0746	259.3626	268.6553	277.823	288.2093	303.5652	318.5574	327.9637	340.2756	354.6503	368.8983	379.8708
Xinjiang	746.85	777.77	820.27	844.65	860.21	933.58	961.67	981.98	1006.93	1058.91	1114.5	1159.47

Highway mileage(Unit: km) (Data from China Statistical Yearbook)												
Region	2005	2006	2007	2008	2009	2010	2011	2012	2013	2014	2015	2016
Beijing	14696	20503	20754	20340	20755	21114	21347	21492	21673	21849	21885	22026
Tianjin	10836	11316	11531	12060	14316	14832	15163	15391	15718	16110	16550	16764
Hebei	75894	143778	147265	149503	152135	154344	156965	163045	174492	179200	184553	188431
Shanxi	69563	112930	119869	124773	127330	131644	134808	137771	139434	140436	140960	142066
nner Mongoli	79029	128762	138610	147288	150756	157994	160995	163763	167515	172167	175374	196061
Liaoning	53521	97786	98101	101144	101117	101545	104026	105562	110973	115430	120365	120613
Jilin	50308	84444	85445	87099	88430	90437	91754	93208	94191	96041	97326	102484
Heilongjiang	67077	139335	140909	150845	151470	151945	155592	159063	160206	162464	163233	164502
Shanghai	8110	10392	11163	11497	11671	11974	12084	12541	12633	12945	13195	13292
Jiangsu	82739	126972	133732	140930	143803	150307	152247	154118	156094	157521	158805	157304
Zhejiang	48600	95310	99812	103652	106952	110177	111776	113550	115426	116367	118015	119053
Anhui	72807	147611	148372	148827	149184	149382	149535	165157	173763	174373	186940	197588
Fujian	58286	86560	86926	88607	89504	91015	92322	94661	99535	101190	104585	106757
Jiangxi	62300	128236	130515	133815	137011	140597	146632	150595	152067	155515	156625	161909
Shandong	80131	204910	212237	220688	226693	229859	233190	244586	252786	259515	263447	265720
Henan	79506	236351	238676	240645	242314	245089	247587	249649	249831	249857	250584	267441
Hubei	91131	181791	183780	188366	197196	206211	212747	218151	226912	236933	252980	260179
Hunan	88200	171848	175415	184568	191405	227998	232190	234040	235392	236250	236886	238273
Guangdong	115337	178387	182005	183155	184960	190144	190724	194943	202915	212094	216023	218085
Guangxi	62003	90318	94202	99273	100491	101782	104889	107906	111384	114900	117993	120547
Hainan	21162	17577	17789	18563	20041	21236	22916	24265	24852	26002	26860	28217
Chongqing	38215	100299	104705	108632	110950	116949	118562	120728	122846	127392	140551	142921
Sichuan	114694	164688	189395	224482	249168	266082	283268	293499	301816	309742	315582	324138
Guizhou	46893	113278	123247	125365	142561	151644	157820	164542	172564	179079	186407	191626
Yunnan	167638	198496	200333	203753	206028	209231	214524	219052	222940	230398	236007	238052
Shaanxi	54492	113303	121297	131038	144109	147461	151986	161411	165249	167145	170069	172471
Gansu	41330	95642	100612	105638	114000	118879	123696	131201	133597	138084	140052	143039
Qinghai	29720	47726	52626	56642	60136	62185	64280	65988	70117	72703	75593	78585
Ningxia	13078	19903	20562	21008	21805	22518	24506	26522	28554	31276	33240	33940
Xinjiang	89531	143736	145219	146652	150683	152843	155150	165909	170155	175468	178263	182085

Total assets of state-owned and state-controlled industrial enterprises(Unit: 100 million yuan) (Data from China Statistical Yearbook)												
Region	2005	2006	2007	2008	2009	2010	2011	2012	2013	2014	2015	2016
Beijing	10105.97	10613.19	12152.07	12218.51	14268.37	16641.07	19370.26	21598.31	22785.56	24400.9	28300.17	31481.11
Tianjin	3267.14	3656.85	4280.33	5422.18	6970.55	7460	8584.99	9516.62	10554.4	11530.97	12141.8	11847.11
Hebei	5054.91	5472.76	6675	8235.07	10210.87	12144.25	13506.43	14637.32	15708.16	16507.43	16484.92	17303.45
Shanxi	4546.47	5463.22	6565.8	8364.84	9745.72	11437.84	14008.51	15476.96	17704.74	19419.96	21080.93	22133.69
nner Mongoli	2788.87	3619.72	4738.9	6129.29	6399.97	7887.52	10263.28	11604.14	12833.43	14449.95	15388.83	15966.88
Liaoning	7274.18	7940.35	9679.89	11899.99	13453.32	14588.59	15462.67	16160.87	17214.92	17791.95	18658.15	18588.12
Jilin	3245.39	3696.63	3711.7	4484.24	4780.21	5623	6521.7	7633.24	8232.19	8797.9	9018.92	9668.02
Heilongjiang	3983.6	4389.05	4874.84	5466.69	6080.25	7114.5	8114.97	8662.87	9095.69	9489.41	9453.65	9007.22
Shanghai	8088.32	8781.05	10030.39	10936.37	12002.67	12770.58	13552.38	14132.78	15337.04	16244.07	17437.33	18841.63
Jiangsu	5519.08	6338.42	7000.51	8297.84	9425.97	11062.36	12934.96	15030.74	16977.67	17763.81	18753.29	19521.06
Zhejiang	3865.38	4319.35	4765.88	5557.97	6135.19	6618.77	7191.77	8138.12	9026.86	9970.37	10617.83	11383.46
Anhui	3282.52	3902.47	4785.59	5998.81	6809.72	8532.68	9798.64	11290.86	12146.48	12933.86	13658.84	14136.94
Fujian	1905.37	2128.63	2636.66	3125.81	3491.23	3845.72	4046.69	4852.61	5906.31	7161.48	7367.82	7841.6
Jiangxi	1944.95	2203.49	2553.61	2859.97	3096.59	3723.89	4239.2	4580.01	5203.7	5199.78	5668.9	5973.82
Shandong	8329.05	10015.43	11020.21	12969.11	14841.52	17179.37	19667.9	21952.47	23668.11	25644.78	28344.5	29156.42
Henan	5396.17	6025.47	7243.07	8300.01	9238.1	10364.68	11531.65	12134.76	12799.42	13622.48	14227.92	14814.67
Hubei	6035.77	6566.74	8407.03	9774.35	12399.78	12074.62	13150.4	13141.5	14862.97	14805	15743.77	17098.93
Hunan	2808.64	3410.97	3952.98	4714.61	4911.2	6266.01	7272.95	7823.38	8380.46	8861.98	9325.62	9937.63
Guangdong	6676.89	8789.27	9702.79	11897.96	12724.56	14797.34	15821.22	16984.9	18225.11	19325.22	21190.57	22869.36
Guangxi	1796.38	1932.13	2629.33	3096.47	3615.47	4368.41	4582.87	5335.87	5897.08	6192.23	6625.75	6958.81
Hainan	429.69	442.33	509.35	434.83	475.76	599.57	597.58	671.13	682.11	805.49	1160.13	1121.66
Chongqing	1991.72	2302.28	2667.23	3067.72	3582.7	4206.02	4776.56	5480.73	6341.35	7197.53	8108.28	8773
Sichuan	4473.03	5109.55	6325.71	8515.93	9515.61	11429.22	13189.13	14797.73	17354.52	19021.19	20092.37	20086.48
Guizhou	2264.1	2566.95	2754.27	3476.32	3771.12	4276.69	5035.78	5634.18	6522.79	7354.07	8062.82	8717.81
Yunnan	2816.97	3265	3856.51	4767.92	5541.4	6471.38	7354.57	8482.38	10227.91	11746.27	12498.44	13590.52
Shaanxi	3739.76	4754.19	5877.15	7681.95	9402.16	11118.22	13181.11	15661.41	16811.48	18875.1	19960.85	21200.59
Gansu	1938.28	2633.23	2889.2	3484.97	4211.17	5224.05	6336.3	7180.68	8051.76	8536.46	8812.7	9005.26
Qinghai	961.73	1156.55	1321.99	1618.28	1937.01	2153.98	2329.96	2637.64	3181.23	3655.16	4032.19	4297.11
Ningxia	747.69	810.56	966.32	1350.33	1693.61	1973.5	2477.17	2663.59	2785.3	3357.41	3605.06	3952.01

Xinjiang	2259.12	2769.14	3488.32	4485.58	4842.07	5594.76	6511.01	7810.18	9072.87	10196.52	10945.75	11688.58

nterprises above designated size(Unit: 100 million yuan) (Data from China Statistical Yearbook, New China 60 years of statistical data compilation, the provincial and mun												
Region	2005	2006	2007	2008	2009	2010	2011	2012	2013	2014	2015	2016
Beijing	12829.79	14244.4	16215.5	16802.42	19540.7	22750.58	25321.75	28613.16	30800.73	33557.05	38609.76	43093.68
Tianjin	6347.92	7129.02	8329.26	10351.21	12617.69	14584.31	17388.98	19986.14	22388.19	23988.63	25242.98	25075.09
Hebei	9473.7	11250.95	13721.58	17261.75	20662.67	24943.75	29687.55	33567.18	37597.12	42555.67	42717.82	44562.88
Shanxi	7045.09	8865.5	10896.32	13452.67	15424.51	18505.94	22186.5	25342.08	28339.9	30574.37	32068.45	33621.95
nner Mongoli	4595.89	5605.92	7512.36	10089.3	11650.94	14691.38	18406.42	21754.23	24376.4	27788.21	29458.05	30900.83
Liaoning	11902.12	14140.89	17034.52	22040.91	25333.81	29076.78	31417.3	34779.77	38665.07	39246.62	38573.04	36106.92
Jilin	4506.88	5449.59	6024.6	7525.18	8525.06	10196.15	11898.88	13896.98	15677.96	16686.6	17993.28	18969.47
Heilongjiang	5174.47	5690.43	6578.79	7826.92	8860.63	10471.17	11918.83	13223.14	14215.77	14995.19	15407.96	14951.92
Shanghai	15905.94	17926.1	20656.75	22750.35	24595.29	27555.88	29454.3	31160.89	33595.79	35512.24	37306.95	39838.24
Jiangsu	25488.86	30500.98	38011.22	48321.94	53600.08	66134.06	76258.16	84550.41	94310.9	101259.53	107061.73	114536.32
Zhejiang	20609.3	24895.59	30581.98	35550.76	39752.79	47282.79	50663.58	55654.17	60436.24	64078.22	66626.71	69468.91
Anhui	5067.1	6234.68	7873.16	10122.18	12171.72	15930.28	19148.71	22797.65	25906.17	28831.52	31359.95	33563.37
Fujian	6841.37	8168.75	10157.2	11694.91	13344.47	16058.7	18582.15	21385.98	24978.49	27978.35	29647.54	32081.3
Jiangxi	3058.34	3671.41	4688.79	6420.95	7018.93	8637.45	10211.32	11967.66	14057.87	16061.44	19217.51	21811.92
Shandong	22131.24	26475.35	31944.91	39224.51	46052.69	53761.28	60818.77	71107.66	81534.78	93330.87	101343.5	105046.32
Henan	9158.03	11026.18	13788	17316.86	19668.61	23467.42	29049.22	35174.81	43431.82	50540.15	55710.97	60454.73
Hubei	8683.39	9694.56	12107.24	15431.43	19221.02	20894.32	23145.87	26877.66	30633.98	32940.84	35399.12	37942.33
Hunan	4611.6	5582.18	6832.01	8856.19	10175.11	13038.95	15473.38	17784.25	20050.53	22025.57	23575.75	25518.07
Guangdong	27076.08	33869.53	39821.97	45750.15	50321.88	62626.9	67371.4	71343.84	79655.27	87590.27	95411.22	105604.17
Guangxi	3009.98	3504.89	4761.72	5981.12	6840.57	8667.45	10185.46	11759.56	13349.56	14225.92	15122.3	16023.46
Hainan	791.28	961.15	1133.35	1207.77	1291.39	1621.38	1747.91	2023.16	2288.03	2444.8	2788.06	2764.18
Chongqing	3091.96	3603.06	4412.77	5551.03	6438.31	8099.01	9321.1	11113.36	13462.11	15652.47	17846.08	20214.63
Sichuan	7908.62	9182.08	11690.21	15589.47	18042.57	22564.76	26113.61	30362.89	36239.56	38359.92	40401.38	41514.58
Guizhou	2734.05	3214.39	3521.19	4566.1	5066.17	5960.13	6990.58	8302.29	10339.98	11747.39	13540.06	14319.98
Yunnan	3964.32	4808.98	5834.12	7185.11	8174.12	9611.09	11053.93	13076.97	15854.9	17458.16	18180.58	19474.18
Shaanxi	5085.87	6130.02	7494.03	9905.91	12119.26	14688.7	17234.61	20591.16	22807.15	26169.19	28227.39	30828.91
Gansu	2483.04	3211.31	3712.44	4497.49	5290.54	6487.35	7665.01	9146.01	10422.52	11348.25	11918.33	12263.36
Qinghai	1145.49	1382.7	1645.94	2092.67	2525.56	3053.61	3386.32	4041.92	4793.88	5414.09	5781.41	6143.77
Ningxia	1113.1	1299.05	1546.93	2061.86	2676.61	3293.16	4044.2	4860.19	5654.6	6976.46	7801.07	8521.18
Xinjiang	2837	3375.02	4336.32	5652.57	6438.07	7911.97	9304.95	11669.17	14331.25	16770.69	18164.16	19538.65

n investment and investment from Hong Kong, Macao and Taiwan(Unit: 100 million yuan) (Data from China Statistical Yearbook, China Industrial Statistical Yearbook, th												
Region	2005	2006	2007	2008	2009	2010	2011	2012	2013	2014	2015	2016
Beijing	2302.55	2621.8	3046.78	3248.21	3890.34	4459.78	5025.54	5582.88	6438.82	7091.93	7644.52	8588.51
Tianjin	2382.3	2828.65	3142.08	3566.69	3914.17	4859.89	6184.58	6766.51	7288.18	7180.32	7303.36	7009.6
Hebei	1549.75	1973.75	2387.92	3000.53	3676.75	4220.6	5117.98	4999.36	5121.83	5245.95	4827.11	4999.98
Shanxi	354.88	453.63	701.71	816.72	958.95	1143.9	1440.92	1755.77	1722.73	1938.07	1914.23	2152.75
nner Mongoli	799.34	720.05	1445.69	1034.66	1365.52	1559.41	1532.41	1804.43	2483.98	2651.38	2779.76	2788.84
Liaoning	2214.1	2713.84	3401.6	4266.98	4876.07	5884.18	6179.6	6853.99	7713.46	7793.65	8148.3	8049.22
Jilin	614.85	795.07	1007.42	1250.35	1443.99	1782.31	2156.77	1703.85	1972.93	2113.2	2115.58	2058.21
Heilongjiang	434.62	608.08	732.49	852.44	1028.36	1157.75	1214.79	1373.33	1457.31	1541.78	1532.35	1653.67
Shanghai	8401.4	9463.55	11159.72	11036.08	11907.44	13761.73	14766.76	15265.08	16189.69	16730.21	16728.52	18037.87
Jiangsu	10409.61	12894.75	16624.16	21168.76	23248.74	28023.91	31666.73	32719.27	35203.7	36685.38	37256.7	38945.68
Zhejiang	5098.38	6472.33	8253.5	9880.74	10387.43	12235.22	13949.73	14623.12	15316.94	15608.07	15302.64	15793.28
Anhui	677.9	890.85	1056.43	1161.06	1474.17	1818.69	2110.2	2257.1	2459.36	2988.16	3266.1	3508.89
Fujian	3719.82	4294.09	5200.28	5805.83	6504.83	7562.52	8572.09	9353.9	10503.06	10833.86	10707.27	11560.53
Jiangxi	434.21	557.11	861.93	1172.3	1438.08	1863.09	1883.16	2043.98	2292.33	2536.8	3000.87	3259.09
Shandong	3844.74	4990.4	6315.81	7401.01	8637.96	9455.5	9736.97	10962.94	11263.37	12789.38	12121.44	13029.04
Henan	618.64	878.38	1076.53	1530.21	1607.14	1933.61	2553.19	3497.36	4303.63	5096.31	5835.75	6845.17
Hubei	1322.8	1566.78	2071.73	2873.53	3886.14	4121.69	4462.54	4734.76	4769.51	5014.31	5170.06	5935.43
Hunan	412.2	491.28	581.49	1165.69	1193.77	1307.52	1115.4	1364.93	1645.93	1788.12	1997.55	2254.99
Guangdong	16004.54	18561.1	22672.27	25094.46	26368.1	33173.08	32802.61	33813.08	36785.64	38530.38	39147.2	40371.71
Guangxi	521.24	618.54	871.2	1094.44	1298.28	1614.39	2117.68	2405.45	2464.92	2576.12	2840.31	2955.39
Hainan	217.08	366.77	438.25	498.94	516.35	609.48	653.25	685.82	755.06	741.01	711.01	720.29
Chongqing	519.13	628.06	827.58	1018.13	1200.07	1535.91	1856.05	2240.46	3276.86	2851.53	3047.8	3585.62
Sichuan	582.87	721.23	1170.1	1367.03	1530	1804.72	2374.03	2922.37	4328.39	3998.06	3692.16	4106.48
Guizhou	91.04	93.02	101.46	152.3	219.45	251.02	278.38	284.84	352.26	363.59	380.15	450.35
Yunnan	218.16	265.68	353.88	401.55	457.94	500.8	561.38	613.11	666.85	702.61	775.27	826.01
Shaanxi	236.76	362.15	499.2	604.62	718.09	900.78	872.04	952.19	1014.85	1574.05	1791.53	1858.7
Gansu	70.81	80.35	124.84	165.79	166.2	220.35	233.25	218.62	265.8	278.54	259.18	305.42
Qinghai	110.91	39.62	40.74	288.24	336.31	390.58	123.7	166.63	200.84	204.8	255.92	235.32
Ningxia	85.68	90.65	101.15	107.31	89.41	237.86	268.33	138.84	149.51	437.42	456.97	480.1
Xinjiang	53.47	61.85	93.32	113.6	129.6	154.2	167.81	206.04	242.26	265.5	278	342.47

Regional financial expenditure(Unit: 100 million yuan) (Data from China Statistical Yearbook)												
Region	2005	2006	2007	2008	2009	2010	2011	2012	2013	2014	2015	2016
Beijing	1058.3114	1296.8389	1649.5023	1959.2857	2319.3658	2717.3174	3245.23	3685.31	4173.66	4524.67	5737.7	6406.77
Tianjin	442.1207	543.1219	674.3262	867.7245	1124.2778	1376.8395	1796.33	2143.21	2549.21	2884.7	3232.35	3699.43
Hebei	979.1635	1180.359	1506.6482	1881.6696	2347.5894	2820.2439	3537.39	4079.44	4409.58	4677.3	5632.19	6049.53
Shanxi	668.7508	915.5698	1049.9228	1315.0175	1561.7047	1931.3641	2363.85	2759.46	3030.13	3085.28	3422.97	3428.86
nner Mongoli	681.8772	812.133	1082.3054	1454.5732	1926.8365	2273.5046	2989.21	3425.99	3686.52	3879.98	4252.96	4512.71
Liaoning	1204.3636	1422.7471	1764.2805	2153.4348	2682.3864	3195.8156	3905.85	4558.59	5197.42	5080.49	4481.61	4577.47
Jilin	631.1212	718.3588	883.7597	1180.1223	1479.2092	1787.2484	2201.74	2471.2	2744.81	2913.25	3217.1	3586.09
Heilongjiang	787.7854	968.5255	1187.2711	1542.3004	1877.738	2253.2694	2794.08	3171.52	3369.18	3434.22	4020.66	4227.34
Shanghai	1646.255	1795.566	2181.678	2593.9161	2989.65	3302.8862	3914.88	4184.02	4528.61	4923.44	6191.56	6918.94
Jiangsu	1673.3965	2013.2502	2553.7217	3247.4927	4017.364	4914.0598	6221.72	7027.67	7798.47	8472.45	9687.58	9981.96

Zhejiang	1265.5345	1471.8593	1806.7928	2208.5756	2653.3486	3207.883	3842.59	4161.88	4730.47	5159.57	6645.98	6974.26
Anhui	713.0633	940.2329	1243.8342	1647.1253	2141.9217	2587.6135	3302.99	3961.01	4349.69	4664.1	5239.01	5522.95
Fujian	593.0663	728.6973	910.6446	1137.7159	1411.8238	1695.0906	2198.18	2607.5	3068.8	3306.7	4001.58	4275.4
Jiangxi	563.9525	696.4361	905.0582	1210.073	1562.3742	1923.2633	2534.6	3019.22	3470.3	3882.7	4412.55	4617.4
Shandong	1466.2271	1833.44	2261.8495	2704.6613	3267.6716	4145.032	5002.07	5904.52	6688.8	7177.31	8250.01	8755.21
Henan	1116.0412	1440.0878	1870.6135	2281.6093	2905.763	3416.1426	4248.82	5006.4	5582.31	6028.69	6799.35	7453.74
Hubei	778.7159	1047.0041	1277.3257	1650.2763	2090.9225	2501.4027	3214.74	3759.79	4371.65	4934.15	6132.84	6422.98
Hunan	873.4181	1064.5177	1357.031	1765.2249	2210.4442	2702.4752	3520.76	4119	4690.89	5017.38	5728.72	6339.16
Guangdong	2289.0691	2553.3399	3159.5703	3778.5681	4334.3727	5421.5432	6712.4	7387.86	8411	9152.64	12827.8	13446.09
Guangxi	611.4806	729.5172	985.9433	1297.11	1621.8218	2007.5907	2545.28	2985.23	3208.67	3479.79	4065.51	4441.7
Hainan	151.2421	174.5366	245.1967	357.9708	486.0624	581.3379	778.8	911.67	1011.17	1099.74	1239.43	1376.48
Chongqing	487.3543	594.2543	768.3886	1016.0112	1292.0928	1709.0353	2570.24	3046.36	3062.28	3304.39	3792	4001.81
Sichuan	1082.1769	1347.3951	1759.1304	2948.8269	3590.7175	4257.9806	4674.92	5450.99	6220.91	6796.61	7497.51	8008.89
Guizhou	520.7261	610.6411	795.399	1053.7922	1372.2654	1631.4792	2249.4	2755.68	3082.66	3542.8	3939.5	4262.36
Yunnan	766.3115	893.5821	1135.2175	1470.2388	1952.3395	2285.7234	2929.6	3572.66	4096.51	4437.98	4712.83	5018.86
Shaanxi	638.9627	824.1805	1053.9665	1428.5208	1841.6388	2218.8283	2930.81	3323.8	3665.07	3962.5	4376.06	4389.37
Gansu	429.3479	528.5946	675.3372	968.4336	1246.2817	1468.581	1791.24	2059.56	2309.62	2541.49	2958.31	3150.03
Qinghai	169.7547	214.6628	282.1993	363.595	486.7457	743.4033	967.47	1159.05	1228.05	1347.43	1515.16	1524.8
Ningxia	160.2509	193.2089	241.8545	324.6064	432.3624	557.5285	705.91	864.36	922.48	1000.45	1138.49	1254.54
Xinjiang	519.0179	678.4723	795.154	1059.3638	1346.9125	1698.9126	2284.49	2720.07	3067.12	3317.79	3804.87	4138.25

Fixed asset investment in state-owned tertiary industry(Unit: 100 million yuan) (Data from China Statistical Yearbook, China Regional Economic Statistical Yearbook)												
Region	2005	2006	2007	2008	2009	2010	2011	2012	2013	2014	2015	2016
Beijing		690.3464	850.4852	849.5983	1345.4	1075.9975	1044.7	1178.2	1450.4	1264.5	1345.2	1131.2
Tianjin		389.3647	513.3965	750.7574	1236.6	1576.0542	1708.4	1909.1	2055.2	1811.9	2290.7	1220.5
Hebei		893.246	997.5801	1027.6838	2036.9	2712.6989	2099.9	2220.1	2604	2836.8	3508.5	3302.4
Shanxi		423.7293	537.3117	760.9508	1482.8	1742.936	1653.8	2067.5	2479.8	2371.3	2486.9	2092.9
nner Mongolia		740.4764	1009.1437	1101.6301	1599.6	1992.198	2153.1	2595.6	3012.3	4196.2	3584.6	4691.4
Liaoning		990.3985	1201.1735	1432.7315	1770.4	2478.2572	2528.3	3099.3	3521.4	3542.6	2529.1	781.8
Jilin		615.3105	761.4615	869.5994	1192.7	1300.9236	1045.8	1453.8	1568.9	1565.7	1795.9	1884.1
Heilongjiang		640.9774	826.3315	1066.0532	1457.4	1784.6004	1681.4	1923.6	2165.3	1955.1	2002.5	2072.1
Shanghai		848.5575	1058.6184	1472.6156	1802.8	1419.3628	1247.6	1274.6	1106.1	1076.2	1275.5	994
Jiangsu		1555.6506	1575.6172	1785.5547	2617.5	3279.0861	3594.5	4369.5	4937.9	6386.7	6639.8	5733
Zhejiang		1436.6925	1386.9216	1587.5387	2060.5	2307.6573	2623	3345.6	3887.6	4314.2	5363.3	4669.4
Anhui		756.5208	1003.6158	1240.0057	1819.8	2058.7891	1897.7	2788.8	3282.4	3879.2	4078.2	4561.1
Fujian		704.249	1027.6343	1274.2982	1702.6	2049.0406	2250.8	2957.5	3226.6	3679.8	4639.8	3668.9
Jiangxi		845.799	836.8781	931.947	1287.2	1576.5292	1528.4	1730.6	1901.2	2535.6	2883	2683.5
Shandong		1231.0865	1213.4038	1571.0628	2147	2472.2279	2456.8	2885	3108.3	3448.9	4171.8	4971.4
Henan		1089.2636	1096.3434	1287.1088	1597	1945.4173	1909.8	2195.8	2604.3	2937.7	3583.7	4075.8
Hubei		821.5804	1058.3186	1297.146	1820.6	2407.7056	2441.7	2805.4	3189	3792.9	4717.6	5574.7
Hunan		785.0825	923.0393	1187.448	2066.2	2502.6592	2545.9	3215.5	3936.1	4612.8	5629	5458.9
Guangdong		1408.5057	1549.813	1849.5283	2655.9	3321.5947	2863.4	2867.4	3242.5	3791.5	4116	3979.5
Guangxi		510.9698	627.2373	788.663	1159.3	1617.0753	1561.8	1797.4	2220.2	2520.5	3181	3240
Hainan		74.6413	98.395	138.6181	234.7	268.2831	344.6	449.2	483.7	548.9	672.3	458.4
Chongqing		489.5784	572.9553	821.9418	1177.3	1630.7661	1712.2	2123.3	2359.3	2750.4	3392	2220.9
Sichuan		1134.3084	1268.7042	1735.0997	3311	3724.0036	3427	4095.1	5281.2	6363.6	7417.5	7339.1
Guizhou		333.2198	389.1468	493.5051	752.4	953.7155	1240.3	1783.4	2517.8	3522.5	4650	2976.4
Yunnan		768.5459	921.0309	992.5466	1440.7	1932.3229	1747	2110	2815.7	3568.4	4839.9	6475.2
Shaanxi		791.4786	978.6063	1262.212	1855	2251.1366	2332.5	3044.9	3855.4	4614.9	5457.5	6763.8
Gansu		322.8611	345.9059	429.686	619.1	831.3741	1060	1122.7	1560.2	1893.5	2191.6	2880.7
Qinghai		119.3495	139.0242	172.1148	257.2	293.1777	420.1	597.3	708.1	985.9	1115.5	1340.9
Ningxia		131.8231	127.4707	181.2439	230.4	274.4836	289.6	335.1	515.3	695	732.5	706.7
Xinjiang		491.3604	449.2263	598.044	856.5	966.3942	1118.7	1529.2	1952.8	2784.3	3381.1	3673.8

Number of health technicians(Unit: 10000 people) (Data from China Statistical Yearbook)												
Region	2005	2006	2007	2008	2009	2010	2011	2012	2013	2014	2015	2016
Beijing	119943	126903	139706	150411	161139	171326	181936	196234	203741	213245	225440	233953
Tianjin	61085	62057	63579	65161	67930	70460	73318	77076	81083	84880	90748	94952
Hebei	229696	234133	243209	247451	268049	292157	301672	314933	333032	351513	372747	393059
Shanxi	146671	149371	145162	159591	186310	193891	191416	199601	203385	209474	213995	225880
nner Mongoli	102587	102336	105790	109727	134988	125831	131603	139876	148202	154483	162327	170406
Liaoning	209346	216457	215491	217904	226425	232079	235623	246808	254692	256284	264419	277494
Jilin	125702	128471	125776	127905	132554	138393	139010	144065	145934	151427	158963	166605
Heilongjiang	150657	151916	158726	161939	175319	192048	195029	201155	207601	212207	215264	221362
Shanghai	103479	109009	120903	127471	132826	137131	140740	147807	157109	164054	170125	178196
Jiangsu	257137	275368	286482	291125	308981	328243	350544	395961	428894	458503	487005	516986
Zhejiang	198148	214622	228425	242908	266254	288481	306922	329565	352466	375902	405620	432641
Anhui	159788	169181	174724	187770	208584	211539	217591	236188	253532	268039	280768	293732
Fujian	100945	106586	94563	103341	130809	142916	158791	176074	197545	206516	212931	219557
Jiangxi	115986	119761	126598	139764	150741	158007	165938	179705	190092	201362	210927	220972
Shandong	323759	336669	340519	375817	414971	448861	481738	530082	596987	603785	618192	641701
Henan	289157	300712	297854	309923	359891	372818	396300	428508	468536	494815	519939	547001
Hubei	215037	217950	227116	233823	246985	255793	268122	288695	309343	335583	367902	384532
Hunan	212483	204011	220578	232084	253547	269219	282511	296857	323082	341404	370788	392547
Guangdong	297334	332829	360656	384134	421325	454799	485585	518414	553728	583009	617975	665257
Guangxi	129151	133924	145579	155620	172910	189554	204011	220761	240892	258599	274659	289872
Hainan	30056	30787	32545	33875	37857	39520	43295	45060	48108	50580	54710	57522
Chongqing	78780	79805	83650	88744	100008	111079	120151	131658	142133	154278	166708	179354
Sichuan	236028	240444	255332	267591	303051	325608	352259	389440	426988	451938	472169	495750
Guizhou	81723	82324	85282	89313	96753	103954	113801	129772	155905	169963	187282	204621
Yunnan	118429	121424	123732	126237	135207	143139	150982	166764	193217	208905	227998	249677

Shaanxi	136550	139065	141687	148328	171840	181438	197173	216293	239054	252611	265381	288607
Gansu	83016	85581	85348	87633	91255	98865	105908	111609	118089	126396	129454	134641
Qinghai	19518	20119	20337	21745	24044	24909	27520	29311	32431	33936	35422	37010
Ningxia	22817	23591	25521	26415	28428	29962	31983	34250	37288	39800	41497	44700
Xinjiang	96266	99839	104671	106853	116028	124055	130604	136691	145851	153417	161841	170987

me personnel equivalent of research and experimental development in various regions(Unit: per person per year) (Data from China Science and Technology Statistical Year												
Region	2005	2006	2007	2008	2009	2010	2011	2012	2013	2014	2015	2016
Beijing	171045	168398	187578	189551	191779	193718.4	217255	235493	242175	245384	245728	253337
Tianjin	33441	37164	44854	48348	52039	58770.7	74293	89609	100219	113335	124321	119384
Hebei	41703	43740	45334	46155	56509	62304.6	73025	78533	89546	100946	106975	111384
Shanxi	27438	38767	36864	43986	47772	46279.1	47355	47029	49035	48955	42873	44147
nner Mongoli	13504	14751	15373	18264	21676	24765.4	27604	31819	37280	36435	38248	39480
Liaoning	66104	69048	77157	76673	80925	84653.8	80977	87180	94885	99586	85366	87839
Jilin	25642	28456	32509	31731	39393	45313.1	44815	49961	48008	49774	49276	48252
Heilongjiang	44203	45068	48205	50717	54159	61854.3	66599	65118	62660	62648	56598	54942
Shanghai	67048	80201	90145	95129	132859	134952.3	148500	153361	165755	168173	171798	183932
Jiangsu	128028	138876	160482	195333	273273	315831	342765	401920	466159	498801	520303	543438
Zhejiang	80120	102761	129393	159589	185069	223484.4	253687	278110	311042	338398	364710	376553
Anhui	28405	29875	36163	49465	59697	64168.7	81087	103047	119342	129319	133558	135829
Fujian	35716	40238	47593	59270	63269	76737.4	96884	114492	122544	135866	126572	132155
Jiangxi	22064	25797	27123	28241	33055	34822.9	37517	38152	43512	43469	46548	50620
Shandong	91142	96637	116470	160420	164620	190329.2	228608	254013	279331	286352	297845	301480
Henan	51181	59692	64879	71494	92571	101467.4	118041	128323	152252	161444	158858	166279
Hubei	61226	62100	67403	72751	91161	97923.7	113920	122748	133061	140741	135481	136608
Hunan	38044	39752	44942	50253	63843	72636.6	85783	100032	103414	107432	114869	119345
Guangdong	119359	147233	199464	238684	283650	344691.8	410805	492327	501718	506862	501696	515649
Guangxi	17947	18940	20141	23243	29856	33987.1	40135	41268	40664	41208	38269	39903
Hainan	1225	1209	1262	1726	4210	4893.1	5397	6787	6962	7514	7713	7840
Chongqing	24619	26826	31563	34421	35005	37078	40698	46122	52612	58354	61520	68055
Sichuan	66382	68584	78849	86736	85921	83800.3	82485	98010	109708	119676	116842	124614
Guizhou	9779	10737	11365	11458	13093	15087.4	15886	18732	23888	23969	23537	24124
Yunnan	14798	16027	17819	19754	21110	22551.5	25092	27817	28483	30523	39535	41116
Shaanxi	53656	59458	65072	64752	68040	73217.8	73501	82428	93494	97138	92618	94755
Gansu	16795	16696	18769	20118	21158	21661	21332	24290	25047	27122	25859	25759
Qinghai	2590	2610	2915	2501	4603	4858.4	5006	5181	4767	4731	4008	4166
Ningxia	4046	4412	5565	5153	6920	6377.7	7358	8073	8234	9500	9247	9004
Xinjiang	6986	7408	8863	8810	12655	14381.7	15451	15671	15822	15662	16949	16945

Total employment（Unit: 10000 people）（Data from the New China 60 years of statistical compilation, the statistical yearbook of provinces and cities）												
Region	2005	2006	2007	2008	2009	2010	2011	2012	2013	2014	2015	2016
Beijing	878	919.7	942.7	980.9	998.3	1031.6	1069.7	1107.3	1141	1156.7	1186.1	1220.1
Tianjin	542.52	562.92	613.93	647.32	677.13	728.7	763.16	803.14	847.46	877.21	896.8	902.42
Hebei	3568.97	3609.99	3664.97	3725.66	3792.49	3865.14	3962.42	4085.74	4183.93	4202.66	4212.5	4223.95
Shanxi	1500.2	1561.2	1595.7	1614.1	1630.6	1685.9	1738.89	1790.17	1844.2	1862.29	1872.76	1908.21
nner Mongoli	1041.1	1051.2	1081.5	1103.3	1142.5	1184.7	1249.3	1304.9	1408.2	1485.4	1463.7	1474
Liaoning	2120.3	2128.1	2180.7	2198.2	2277.1	2317.5	2364.9	2423.8	2518.9	2562.2	2409.9	2301.2
Jilin	1238.9	1250.5	1266.1	1281.4	1297.3	1311.6	1337.78	1355.9	1415.43	1447.17	1480.6	1501.73
Heilongjiang	1748.9	1784.1	1827.6	1852.4	1877	1932	1977.8	2027.8	2060.4	2079.7	2034.5	2077.3
Shanghai	969.24	1005.24	1024.33	1053.24	1064.42	1090.76	1104.33	1115.5	1368.91	1365.63	1361.51	1365.24
Jiangsu	4578.75	4628.95	4677.88	4700.96	4726.54	4754.68	4758.23	4759.53	4759.89	4760.83	4758.5	4756.22
Zhejiang	3100.76	3172.38	3405.01	3486.53	3591.98	3636.02	3674.11	3691.24	3708.73	3714.15	3733.65	3760
Anhui	3669.7	3741	3818	3916	3988	4050	4120.9	4206.8	4275.9	4311	4342.1	4361.6
Fujian	1868.5	1949.58	2015.33	2079.78	2168.86	2241.59	2459.99	2568.93	2555.86	2648.51	2768.41	2797.03
Jiangxi	2276.7	2321.1	2369.6	2404.5	2445.2	2498.8	2532.6	2556	2588.7	2603.3	2615.8	2637.6
Shandong	5840.7	5960	6081.4	6187.6	6294.2	6401.9	6485.6	6554.3	6580.4	6606.5	6632.5	6649.7
Henan	5662	5719	5773	5835	5949	6042	6198	6288	6387	6520	6636	6726
Hubei	3537	3564	3584	3607	3622	3645	3672	3687	3692	3687.5	3658	3633
Hunan	3801.48	3842.17	3883.41	3910.06	3935.21	3982.73	4005.03	4019.31	4036.45	4044.13	3980.3	3920.41
Guangdong	5022.97	5177.02	5341.5	5471.72	5688.62	5870.48	5960.74	5965.95	6117.68	6183.23	6219.31	6279.22
Guangxi	2703	2760	2769	2799	2848	2903	2936	2768	2782	2796	2820	2841
Hainan	379.55	389.03	397.46	408.36	424.56	439.65	459.22	483.9	514.56	543.1	555.77	558.14
Chongqing	1456.3	1454.77	1468.87	1492.43	1513	1539.95	1585.16	1633.14	1683.51	1696.94	1707.37	1717.52
Sichuan	4702	4715	4731.1	4740	4756.62	4772.5261	4785.47	4798.3	4817.31	4833	4847.01	4860
Guizhou	2220	2235	1872.64	1867.2	1841.92	1770.9	1792.8	1825.82	1864.21	1909.69	1946.65	1983.72
Yunnan	2461.3	2517.6	2573.8	2638.37	2684.8	2765.9	2857.24	2881.9	2912.36	2962.25	2942.5	2998.89
Shaanxi	1976	1986	2013	2039	2060	2074	2059	2061	2058	2067	2071	2073
Gansu	1391.36	1401.36	1414.76	1446.34	1488.63	1499.56	1500.26	1491.59	1504.97	1519.86	1535.69	1548.74
Qinghai	291.04	294.19	298.56	301	303.26	307.65	309.18	310.89	314.21	317.3	321.41	324.28
Ningxia	299.6	308.1	309.5	303.9	328.5	326	339.6	344.5	351.3	357.2	362.2	369.2
Xinjiang	791.62	811.75	830.42	847.58	866.15	894.65	953.34	1010.44	1096.59	1135.24	1195.06	1263.11

Number of elementary school degrees(Unit: people) (Data from China Financial Yearbook)												
Region	2005	2006	2007	2008	2009	2010	2011	2012	2013	2014	2015	2016
Beijing	28028	1887	1850	1898	1878	1952619	1742	1627	1692	1772	32650	1631
Tianjin	28890	1984	1789	1794	1759	2205954	1837	1885	1894	1962	35688	1902
Hebei	254380	18523	17772	17335	16223	17719711	14771	13858	13798	14382	274871	14776
Shanxi	124513	7946	7743	7429	7052	7804836	6518	6039	6385	6185	110913	5620
nner Mongoli	83456	6324	6249	5945	5577	6278818	5082	4794	4918	5046	89268	4312
Liaoning	145201	9816	9818	9538	8485	9364681	7869	7069	6401	6759	131839	6838

Jilin	104191	6935	6576	5988	6028	6604755	6163	5340	5046	5171	100760	5106
Heilongjiang	138865	10011	8995	8835	8081	9211366	7505	7476	6674	6968	134603	6952
Shanghai	36037	2251	2326	2299	2166	3121808	2633	2403	2654	2519	46582	2528
Jiangsu	270481	19809	18943	18053	17778	19033077	15096	14701	14527	14189	270803	14012
Zhejiang	218873	14588	14599	14405	14001	15687367	12682	11640	11329	12175	239672	12079
Anhui	254019	17313	16923	16690	15505	16518988	13720	13071	13022	12497	245444	13091
Fujian	159882	11445	11328	11161	10092	10994768	9413	9231	8746	8745	160778	9407
Jiangxi	209474	14951	13306	13056	11817	13399357	10562	10233	9105	10003	195947	10875
Shandong	331391	24377	22941	22367	21705	23912234	19602	19465	18912	17650	345693	18766
Henan	327316	22770	21676	19928	19769	22669174	19191	17865	18142	18146	350004	17933
Hubei	227281	15311	14433	14376	13274	13092079	11150	10585	10067	11160	203950	10885
Hunan	262644	19157	17783	17181	16228	17600932	14722	15225	14311	14146	259381	13315
Guangdong	372059	25865	23909	23203	22200	23788746	19618	18921	18284	18495	352377	18686
Guangxi	224461	14696	14229	14055	13612	14579571	11836	12085	11385	10547	210679	10350
Hainan	31312	2045	1996	1867	1859	1958821	1563	1518	1319	1420	27457	1570
Chongqing	147268	10345	9846	9424	9303	9707595	7869	7865	7595	7385	139391	7581
Sichuan	439004	31263	29681	28278	27099	27846551	22847	20810	22277	21926	399659	21287
Guizhou	200332	14169	14127	13495	13391	13546008	10837	9841	8982	8758	172357	9233
Yunnan	255810	17830	18081	18386	17080	20042984	15759	14876	14753	14943	255239	14306
Shaanxi	145308	9837	9414	9264	8917	8740957	7339	6841	6615	6663	126830	6847
Gansu	118587	7867	7765	7901	7697	8313301	6885	6796	6747	6573	115600	6813
Qinghai	22575	1754	1784	1877	1874	1984288	1699	1613	1532	1514	29610	1602
Ningxia	24015	1719	1679	1673	1558	1868716	1546	1644	1427	1515	26334	1394
Xinjiang	85226	6187	6149	6039	5875	6563203	5147	5362	5205	4799	98351	5455

Number of high school and technical secondary school graduates(Unit: people) (Data from China Financial Yearbook)												
Region	2005	2006	2007	2008	2009	2010	2011	2012	2013	2014	2015	2016
Beijing	49266	3188	3207	3319	3290	4161674	3645	3659	3321	3684	63565	3258
Tianjin	27873	2118	2227	2404	2431	2672387	2503	2450	2483	2712	47631	2821
Hebei	101848	6801	6678	7030	6556	9131670	8296	8059	8452	8032	169590	8625
Shanxi	54343	4279	4241	4292	4441	5618618	5412	5766	4866	5410	103975	5907
nner Mongoli	45675	3093	3128	2886	3041	3740299	3490	3369	3283	3132	59976	3395
Liaoning	73033	5511	5427	5329	5247	6469305	5785	5127	5535	5769	102177	5787
Jilin	51242	4071	4017	4219	3747	4630133	3980	4069	3942	4031	67412	3653
Heilongjiang	70264	5030	5170	5206	5005	5756969	4638	5013	5324	4784	85478	4972
Shanghai	56630	4144	4223	4101	4106	4823221	4168	4046	3868	3951	75072	4104
Jiangsu	138515	10071	9866	10331	9808	12703757	10873	11062	11346	10802	210556	12036
Zhejiang	74175	5568	5490	5461	5727	7384253	5833	6733	6428	5788	114841	6890
Anhui	69520	5087	5028	5310	5268	6449846	5755	5733	6191	6948	122498	6226
Fujian	53429	3810	4132	3925	4191	5119371	3969	4565	4443	4294	86095	4570
Jiangxi	56016	4163	5261	5382	5937	5488361	5982	6114	7546	6040	102460	6011
Shandong	144346	10767	10747	11468	10402	13322584	11427	11530	12514	14062	232188	13025
Henan	131090	9245	10219	10565	10389	12423541	11359	10457	10877	11546	227753	12638
Hubei	96636	7692	7866	7882	7841	9503078	8111	8844	10032	8894	154787	8563
Hunan	108558	7633	8258	8165	7843	10133885	8826	8746	9080	8969	195582	11213
Guangdong	176299	12144	13151	13684	14766	18267539	16788	17162	16733	17540	335850	18179
Guangxi	56149	4643	4293	4163	4270	5078715	4737	4302	4538	5518	92179	5616
Hainan	14558	965	996	1029	949	1288237	1111	1167	1345	1378	23040	1253
Chongqing	35557	2681	2707	2564	2821	3814455	3647	3421	3653	4141	75556	4287
Sichuan	78538	6605	7071	7012	7192	9045928	7623	8289	8290	8696	163857	8958
Guizhou	29927	2032	2324	2354	2180	2617659	1982	2720	2818	2773	51158	2967
Yunnan	33857	2579	2640	2571	2681	3813068	3258	4401	3497	3569	78036	4060
Shaanxi	65714	4960	5199	5023	4636	5887718	4967	5552	5331	5882	87981	5701
Gansu	35882	2608	2790	2550	2607	3244504	2996	3109	2820	2612	60103	3310
Qinghai	7161	503	514	483	467	586713	554	497	511	533	9219	540
Ningxia	8534	620	637	656	667	792711	685	631	778	717	14059	844
Xinjiang	29764	1972	2057	2029	2052	2545639	2522	2229	2147	2548	45655	2618

Regional fiscal revenue(Unit: 10000 yuan） (Data from China Financial Yearbook)												
Region	2005	2006	2007	2008	2009	2010	2011	2012	2013	2014	2015	2016
Beijing	9192098	11171514	14926380	18373238	20268089	23539301	30062800	33149300	36611100	40271600	47238600	50812600
Tianjin	3318507	4170479	5404390	6756186	8219916	10688093	14551300	17600200	20790700	23903500	26671100	27235000
Hebei	5157017	6205340	7891198	9475858	10671231	13318547	17377700	20842800	22956200	24466200	26491800	28498700
Shanxi	3683437	5833752	5978870	7480047	8058279	9696652	12134300	15163800	17016200	18206400	16423500	15570000
nner Mongoli	2774553	3433774	4923615	6506764	8508588	10699776	13566700	15527500	17209800	18436700	19644800	20164300
Liaoning	6752768	8176718	10826948	13560812	15912197	20048352	26431500	31053800	33438100	31927800	21273900	22004900
Jilin	2071520	2452045	3206892	4227961	4870943	6024092	8501000	10412500	11569600	12033800	12293500	12637800
Heilongjiang	3182056	3868440	4404689	5782773	6416627	7555788	9975500	11631700	12774000	13013100	11658800	11484100
Shanghai	14173976	15760742	20744792	23587464	25402975	28735840	34298300	37437100	41095100	45855500	55195000	64061300
Jiangsu	13226753	16566820	22377276	27314074	32287800	40798595	51489100	58606900	65684600	72331400	80285900	81212300
Zhejiang	10665964	12982044	16494981	19333890	21425131	26084655	31508000	34412300	37969200	41220200	48099400	53019800
Anhui	3340170	4280265	5436973	7246197	8639175	11493952	14635600	17927200	20750800	22184400	24543000	26727900
Fujian	4326003	5411707	6994577	8334032	9324282	11514923	15015100	17761700	21194500	23622100	25442400	26548300
Jiangxi	2529236	3055214	3898510	4886476	5813012	7780922	10534300	13719900	16212400	18818300	21657400	21514700
Shandong	10731250	13562526	16753980	19570541	21986324	27493842	34559300	40594300	45599500	50268300	55293300	58601800
Henan	5376514	6791715	8620804	10089009	11260638	13813178	17217600	20403300	24154500	27392600	30160500	31534700
Hubei	3755217	4760823	5903552	7108492	8148653	10112314	15269100	18230500	21912200	25669000	30055300	31020600
Hunan	3952651	4779274	6065508	7227122	8476178	10816901	15170700	17821600	20308800	22627900	25154300	26978800
Guangdong	18072044	21794608	27858007	33103235	36498110	45170445	55148400	62291800	70814700	80650800	93667800	103903500
Guangxi	2830359	3425788	4188265	5184245	6209888	7719918	9477200	11660600	13176000	14222800	15151600	15562700
Hainan	686802	818139	1082935	1448584	1782420	2709915	3401200	4094400	4810100	5553100	6277000	6375100

Chongqing	2568072	3177165	4427000	5775738	6551701	9520745	14883300	17034900	16932400	19220200	21548300	22279100
Sichuan	4796635	6075850	8508606	10416603	11745927	15616727	20447900	24212700	27841000	30610700	33554400	33888500
Guizhou	1824963	2268157	2851375	3478416	4164761	5337309	7730800	10140500	12064100	13666700	15033800	15613400
Yunnan	3126490	3799702	4867146	6140518	6982525	8711875	11111600	13381500	16113000	16980600	18081500	18122900
Shaanxi	2753183	3624805	4752398	5914750	7352704	9582065	15001800	16006900	17483300	18904000	20599500	18339900
Gansu	1235026	1412152	1909107	2649650	2865898	3535833	4501200	5204000	6072700	6726700	7438600	7869700
Qinghai	338222	422437	567083	715692	877381	1102153	1518100	1864200	2238600	2516800	2671300	2385100
Ningxia	477216	613570	800312	950090	1115755	1535507	2199800	2639600	3083400	3398600	3734500	3876600
Xinjiang	1803184	2194628	2858600	3610616	3887848	5005759	7204300	9089700	11284900	12823400	13308500	12989500

Balance of RMB loans from financial institutions(Unit: 100 million yuan) (Data from provincial and municipal statistical yearbooks)												
Region	2005	2006	2007	2008	2009	2010	2011	2012	2013	2014	2015	2016
Beijing	13834.4559	15632.7	17812.5	19985	25421.8	29563.8	33367	36441.3	40506.7	45458.7	50559.5	56618.9
Tianjin	4457.63	5182.76	6241.07	7383.29	10645.32	13111.57	15242.17	17392.06	19453.31	21715.99	24500.91	27367.97
Hebei	6415.23	7411.88	8397.82	9453.3	13123.8	15755.74	18143.99	21317.96	24423.22	28052.29	32608.47	37745.85
Shanxi	4228.9987	4788.5141	5394.468	5960.3272	7814.739	9634.3196	11169.354	13106.206	14887.531	16432.745	18458.665	20228.578
nner Mongoli	2588.5704	3205.1943	3767.736	4527.8595	6292.5233	7919.4745	9727.7025	11284.199	12944.169	14947.074	17140.674	19361.014
Liaoning	7958.0512	9117.2178	10403.882	11794.6	15549.6	18689.8	22831.7	26306.5	29722	33023.5	36282.8	38685.6
Jilin	3332.93	3870.33	4306.01	4835.89	6234.66	7205.94	8126.17	9155.6	10696.52	12587.26	15203.11	17141.06
Heilongjiang	3658.5	3971.9	4256.4	4532.7	5988.3	7230.5	8548.7	9906.7	11359.4	13391.7	16214.9	17725
Shanghai	14380.39	15982.28	18595.8	21236.21	29684.1	34154.17	37196.79	40982.48	44357.88	47915.81	53387.21	59982.25
Jiangsu	15396.59	18485.02	22092.1	26160.72	35296.73	42121.04	47868.3	54412.3	61836.53	69572.67	78866.34	91107.6
Zhejiang	16557.67	20153.94	24144.42	28958.36	37997.98	45288.07	51276.64	56982.64	62597.56	68566.32	74070.2	79926.05
Anhui	4313.55	5132.02	6042.51	6948.7	9289.4	11452.29	13729.83	16294.28	19088.8	22088.3	25489.05	30180.73
Fujian	5068.68	6447.72	8065.67	9585.92	12360.32	15231.36	18165.19	21209.82	24487.53	28417.7	32132.96	36356.06
Jiangxi	3019.0027	3460.8033	4026.7439	4544.8353	6346.9937	7757.1237	9175.1587	10924.547	12953.47	15466.109	18347.996	21721.766
Shandong	13381.7463	15709.6014	17545.147	20053.91	25961.323	30722.636	35178.999	40021.492	44761.264	50058.644	55436.999	61726.88
Henan	7434.53	8567.33	9545.48	10368.05	13437.43	15871.32	17506.24	20301.72	23511.41	27228.27	31432.62	36501.17
Hubei	5649.67	6430.44	7496.46	8465.63	11659.37	14136.58	15662.54	18004.54	20796.86	24239.96	28338.9	34530.72
Hunan	4509.0863	5173.8675	6037.3983	6989.4225	9369.8079	11303.761	13462.505	15648.586	18141.132	20783.097	24221.877	27532.3
Guangdong	20965.55	23617.49	27497.88	30964.62	39683.65	47191.56	53411.83	59967.26	68491.93	77889.5	89289.27	103649.79
Guangxi	3104.6	3636.9	4331.03	5110.06	7360.43	8979.87	10646.43	12355.52	14081.01	16070.95	18119.3	20640.54
Hainan	874.3246	993.7819	1086.8887	1219.6773	1728.8123	2266.5488	2793.7865	3381.5972	3978.1513	4684.315	5685.14	6569.47
Chongqing	3719.52	4388.28	5131.69	6320.81	8766.06	10888.15	13001.39	15131.22	17381.55	20011.498	22393.93	24785.19
Sichuan	6743	7833.32	9200.93	11163.39	15680.33	19129.79	22514.23	26163.25	30298.85	34750.72	38703.99	43543.015
Guizhou	2303.93	2696.11	3128.63	3569.27	4656.5	5747.53	6841.92	8274.78	10104.3	12368.298	15051.94	17857.801
Yunnan	3987.5767	4803.5098	5671.6646	6594.3329	8779.6277	10568.777	12114.59	13848.1	15782.46	17978.74	20842.86	23056.28
Shaanxi	3983.192	4463.2109	5121.163	6056.8198	8276.6417	9971.5567	11796.578	13865.61	16219.84	18837.2	21760.61	23921.75
Gansu	1923.4608	2112.0802	2403.6323	2731.8936	3649.6154	4433.05	5468.81	6829.42	8430.08	10681.63	13292.18	15650.47
Qinghai	638.5881	723.179	873.1493	1025.631	1399.0177	1822.65	2231.52	2791.68	3398.17	4171.73	4988.01	5579.76
Ningxia	833.88	983.37	1184.57	1402.56	1917.4	2398.7	2860.58	3339.58	3910.15	4578.49	5117.82	5667.89
Xinjiang	2272.08	2412.69	2685	2826.53	3782.92	4973.16	6270.21	7914	9840.46	11671.39	13041	14552.71

Chinese per capita GDP(Ubit: yuan) (Data from China Financial Yearbook)												
Region	2005	2006	2007	2008	2009	2010	2011	2012	2013	2014	2015	2016
Beijing	14368	16738	20505	24121	26222	30876	36403	40007	43852	47203	50251	53980
Tianjin	14368	16738	20505	24121	26222	30876	36403	40007	43852	47203	50251	53980
Hebei	14368	16738	20505	24121	26222	30876	36403	40007	43852	47203	50251	53980
Shanxi	14368	16738	20505	24121	26222	30876	36403	40007	43852	47203	50251	53980
nner Mongoli	14368	16738	20505	24121	26222	30876	36403	40007	43852	47203	50251	53980
Liaoning	14368	16738	20505	24121	26222	30876	36403	40007	43852	47203	50251	53980
Jilin	14368	16738	20505	24121	26222	30876	36403	40007	43852	47203	50251	53980
Heilongjiang	14368	16738	20505	24121	26222	30876	36403	40007	43852	47203	50251	53980
Shanghai	14368	16738	20505	24121	26222	30876	36403	40007	43852	47203	50251	53980
Jiangsu	14368	16738	20505	24121	26222	30876	36403	40007	43852	47203	50251	53980
Zhejiang	14368	16738	20505	24121	26222	30876	36403	40007	43852	47203	50251	53980
Anhui	14368	16738	20505	24121	26222	30876	36403	40007	43852	47203	50251	53980
Fujian	14368	16738	20505	24121	26222	30876	36403	40007	43852	47203	50251	53980
Jiangxi	14368	16738	20505	24121	26222	30876	36403	40007	43852	47203	50251	53980
Shandong	14368	16738	20505	24121	26222	30876	36403	40007	43852	47203	50251	53980
Henan	14368	16738	20505	24121	26222	30876	36403	40007	43852	47203	50251	53980
Hubei	14368	16738	20505	24121	26222	30876	36403	40007	43852	47203	50251	53980
Hunan	14368	16738	20505	24121	26222	30876	36403	40007	43852	47203	50251	53980
Guangdong	14368	16738	20505	24121	26222	30876	36403	40007	43852	47203	50251	53980
Guangxi	14368	16738	20505	24121	26222	30876	36403	40007	43852	47203	50251	53980
Hainan	14368	16738	20505	24121	26222	30876	36403	40007	43852	47203	50251	53980
Chongqing	14368	16738	20505	24121	26222	30876	36403	40007	43852	47203	50251	53980
Sichuan	14368	16738	20505	24121	26222	30876	36403	40007	43852	47203	50251	53980
Guizhou	14368	16738	20505	24121	26222	30876	36403	40007	43852	47203	50251	53980
Yunnan	14368	16738	20505	24121	26222	30876	36403	40007	43852	47203	50251	53980
Shaanxi	14368	16738	20505	24121	26222	30876	36403	40007	43852	47203	50251	53980
Gansu	14368	16738	20505	24121	26222	30876	36403	40007	43852	47203	50251	53980
Qinghai	14368	16738	20505	24121	26222	30876	36403	40007	43852	47203	50251	53980
Ningxia	14368	16738	20505	24121	26222	30876	36403	40007	43852	47203	50251	53980
Xinjiang	14368	16738	20505	24121	26222	30876	36403	40007	43852	47203	50251	53980

Note: The above data can be found on the China Knowledge Network China Economic and Social Big Data Research Platform, which brings together the China Statistical Yearbook, China Science and Technology Statistical Yearbook, China Population and Employment Statistical Yearbook, China Financial Yearbook and many other yearbooks in previous years. The data needed for this paper basically come from this platform, which is located at the following URL: https://data.cnki.net/yearbook?type=type&code=a.
