# Supplementary material for: An empirical analysis of the impact of Chinese government investment on high-quality economic development——A study based on spatial Dubin model
Source: PLoS One. 2023 Mar 15;18(3):e0283073. doi: 10.1371/journal.pone.0283073 (PMC10016692; doi:10.1371/journal.pone.0283073)
Supplement: S2 File — This file is mainly a description of the underlying data sources used in this paper. (PDF) [file pone.0283073.s002.pdf]

Description of data

The basic data used in this paper are basically derived from public information such as China Statistical Yearbook, China Industrial Statistical Yearbook, China Fixed Assets Statistical Yearbook, China Tertiary Industry Statistical Yearbook, New China 60 Years Statistical Data Compilation, and the statistical yearbooks of various provinces and cities in the past years, etc. A few missing data were estimated by interpolation, regression analysis and other methods. Public information we can find from the China Knowledge Network China's economic and social big data research platform at https://data.cnki.net/. In addition, the China Statistical Yearbook can be found on the official website of the National Bureau of Statistics of China at http://www.stats.gov.cn/tjsj/ndsj/. Provincial and municipal statistical yearbooks can be found on the official websites of provincial and municipal statistical bureaus, with the exception of Xinjiang, the links to other provinces, cities and autonomous regions are as follows.

China Statistical Yearbook: https://data.cnki.net/yearBook/single?id=N2022110021

China Industrial Statistical Yearbook: https://data.cnki.net/yearBook/single?id=N2022010304

China Science and Technology Statistical Yearbook: https://data.cnki.net/yearBook/single?id=N2022010277

China Population and Employment Statistics Yearbook: https://data.cnki.net/yearBook/single?id=N2022040097

China Environmental Statistical Yearbook: https://data.cnki.net/yearBook/single?id=N2022030234

China Energy Statistics Yearbook: https://data.cnki.net/yearBook/single?id=N2022030234

New China 60 years of statistical data compilation: https://data.cnki.net/yearBook/single?id=N2010042091

China Trade and Foreign Economic Statistics Yearbook: https://data.cnki.net/yearBook/single?id=N2022010261

China Labor Statistics Yearbook: https://data.cnki.net/yearBook/single?id=N2022020102

China Real Estate Statistical Yearbook: https://data.cnki.net/yearBook/single?id=N2022010276

China Construction Industry Statistical Yearbook: https://data.cnki.net/yearBook/single?id=N2021120002

China Financial Yearbook: https://data.cnki.net/yearBook/single?id=N2022040012

China Regional Economic Statistics Yearbook: https://data.cnki.net/yearBook/single?id=N2015070200

In addition, the China Statistical Yearbook can be accessed on the official website of the National Bureau of Statistics of China at http://www.stats.gov.cn/tjsj/ndsj/. Provincial and municipal statistical yearbooks can be found on the official websites of provincial and municipal statistical bureaus, with the exception of Xinjiang, the links to other provinces, cities and autonomous regions are as follows:

Beijing Statistical Yearbook: http://tjj.beijing.gov.cn/tjsj_31433/;

Tianjin Statistical Yearbook: https://stats.tj.gov.cn/tjsj_52032/tjnj/

Hebei Economic Yearbook: http://www.hetj.gov.cn/hetj/tjsj/jjnj/

Shanxi Statistical Yearbook: http://tjj.shanxi.gov.cn/tjsj/

Inner Mongolia Statistical Yearbook:
http://tj.nmg.gov.cn/datashow/pubmgr/publishmanage.htm?m=queryPubData&procode=0003

Liaoning Statistical Yearbook: https://tjj.ln.gov.cn/tjj/tjxx/xxcx/index.shtml

Jilin Statistical Yearbook: http://tjj.jl.gov.cn/tjsj/tjnj/

Heilongjiang Statistical Yearbook: http://tjj.hlj.gov.cn/tjj/c106782/common_zfxxgk.shtml

Shanghai Statistical Yearbook: https://tjj.sh.gov.cn/tjnj/index.html

Jiangsu Statistical Yearbook: http://tj.jiangsu.gov.cn/col/col87172/index.html

Zhejiang Statistical Yearbook: http://tjj.zj.gov.cn/col/col1525563/index.html

Anhui Statistical Yearbook: http://tjj.ah.gov.cn/ssah/qwfbjd/tjnj/index.html

Fujian Statistical Yearbook: https://tjj.fujian.gov.cn/xxgk/ndsj/

Jiangxi Statistical Yearbook: http://tjj.jiangxi.gov.cn/col/col38595/index.html

Shandong Statistical Yearbook: http://tjj.shandong.gov.cn/col/col6279/index.html

Henan Statistical Yearbook: https://tjj.henan.gov.cn/tjfw/tjcbw/tjnj/

Hubei Statistical Yearbook: http://tjj.hubei.gov.cn/tjsj/sjkscx/tjnj/qstjnj/

Hunan Statistical Yearbook: http://tjj.hunan.gov.cn/hntj/tjsj/tjnj/index.html

Guangdong Statistical Yearbook: http://stats.gd.gov.cn/gdtjnj/

Guangxi Statistical Yearbook: http://tjj.gxzf.gov.cn/tjsj/tjnj/

Hainan Statistical Yearbook: http://stats.hainan.gov.cn/tjj/tjsu/ndsj/

Chongqing Statistical Yearbook: http://tjj.cq.gov.cn/zwgk_233/tjnj/

Sichuan Statistical Yearbook: http://tjj.sc.gov.cn/scstjj/c105855/nj.shtml

Guizhou Statistical Yearbook:
http://stjj.guizhou.gov.cn/tjsj_35719/sjcx_35720/gztjnj_40112/tjnj2018/

Yunnan Statistical Yearbook: http://stats.yn.gov.cn/tjsj/tjnj/index.html

Shaanxi Statistical Yearbook: http://tjj.shaanxi.gov.cn/tjsj/ndsj/tjnj/

Gansu Statistical Yearbook: http://tjj.gansu.gov.cn/tjj/c109464/info_disp.shtml

Qinghai Statistical Yearbook: http://tjj.qinghai.gov.cn/tjData/qhtjnj/

Ningxia Statistical Yearbook:
http://nxdata.com.cn/publish.htm?m=getMorePublish&bc=A01&cn=G01
